# The Effect of Including Benchmark Prevalence Data of Common Imaging Findings in Spine Image Reports on Health Care Utilization Among Adults Undergoing Spine Imaging: A Stepped-Wedge Randomized Clinical Trial

**DOI:** 10.1001/jamanetworkopen.2020.15713

**Published:** 2020-09-04

**Authors:** Jeffrey G. Jarvik, Eric N. Meier, Kathryn T. James, Laura S. Gold, Katherine W. Tan, Larry G. Kessler, Pradeep Suri, David F. Kallmes, Daniel C. Cherkin, Richard A. Deyo, Karen J. Sherman, Safwan S. Halabi, Bryan A. Comstock, Patrick H. Luetmer, Andrew L. Avins, Sean D. Rundell, Brent Griffith, Janna L. Friedly, Danielle C. Lavallee, Kari A. Stephens, Judith A. Turner, Brian W. Bresnahan, Patrick J. Heagerty

**Affiliations:** 1 Department of Radiology, University of Washington, Seattle; 2 Department of Neurological Surgery, University of Washington, Seattle; 3 Department of Health Services, University of Washington, Seattle; 4 Comparative Effectiveness, Cost, and Outcomes Research Center, University of Washington, Seattle; 5 Department of Biostatistics, University of Washington, Seattle; 6 Center for Biomedical Statistics, University of Washington, Seattle; 7 Flatiron Health, New York, New York; 8 Rehabilitation Care Services, VA Puget Sound Health Care System, Seattle, Washington; 9 Department of Rehabilitation Medicine, University of Washington, Seattle; 10 Department of Radiology, Mayo Clinic, Rochester, Minnesota; 11 Kaiser Permanente Washington, Seattle; 12 Departments of Family Medicine and Internal Medicine, Oregon Health and Science University, Portland; 13 Department of Radiology, Henry Ford Hospital, Detroit, Michigan; 14 Department of Radiology, Stanford University School of Medicine, Palo Alto, California; 15 Division of Research, Kaiser Permanente Northern California, Oakland, California; 16 Surgical Outcomes Research Center, University of Washington, Seattle; 17 Department of Psychiatry and Behavioral Sciences, University of Washington, Seattle

## Abstract

**Importance:**

Lumbar spine imaging frequently reveals findings that may seem alarming but are likely unrelated to pain. Prior work has suggested that inserting data on the prevalence of imaging findings among asymptomatic individuals into spine imaging reports may reduce unnecessary subsequent interventions.

**Objective:**

To evaluate the impact of including benchmark prevalence data in routine spinal imaging reports on subsequent spine-related health care utilization and opioid prescriptions.

**Design, Setting, and Participants:**

This stepped-wedge, pragmatic randomized clinical trial included 250 401 adult participants receiving care from 98 primary care clinics at 4 large health systems in the United States. Participants had imaging of their backs between October 2013 and September 2016 without having had spine imaging in the prior year. Data analysis was conducted from November 2018 to October 2019.

**Interventions:**

Either standard lumbar spine imaging reports (control group) or reports containing age-appropriate prevalence data for common imaging findings in individuals without back pain (intervention group).

**Main Outcomes and Measures:**

Health care utilization was measured in spine-related relative value units (RVUs) within 365 days of index imaging. The number of subsequent opioid prescriptions written by a primary care clinician was a secondary outcome, and prespecified subgroup analyses examined results by imaging modality.

**Results:**

We enrolled 250 401 participants (of whom 238 886 [95.4%] met eligibility for this analysis, with 137 373 [57.5%] women and 105 497 [44.2%] aged >60 years) from 3278 primary care clinicians. A total of 117 455 patients (49.2%) were randomized to the control group, and 121 431 patients (50.8%) were randomized to the intervention group. There was no significant difference in cumulative spine-related RVUs comparing intervention and control conditions through 365 days. The adjusted median (interquartile range) RVU for the control group was 3.56 (2.71-5.12) compared with 3.53 (2.68-5.08) for the intervention group (difference, −0.7%; 95% CI, −2.9% to 1.5%; *P* = .54). Rates of subsequent RVUs did not differ between groups by specific clinical findings in the report but did differ by type of index imaging (eg, computed tomography: difference, −29.3%; 95% CI, −42.1% to −13.5%; magnetic resonance imaging: difference, −3.4%; 95% CI, −8.3% to 1.8%). We observed a small but significant decrease in the likelihood of opioid prescribing from a study clinician within 1 year of the intervention (odds ratio, 0.95; 95% CI, 0.91 to 1.00; *P* = .04).

**Conclusions and Relevance:**

In this study, inserting benchmark prevalence information in lumbar spine imaging reports did not decrease subsequent spine-related RVUs but did reduce subsequent opioid prescriptions. The intervention text is simple, inexpensive, and easily implemented.

**Trial Registration:**

ClinicalTrials.gov Identifier: NCT02015455

## Key Points

**Question:**

What is the impact of including benchmark prevalence data of common findings in reports of spinal imaging ordered by primary care clinicians?

**Findings:**

In this randomized clinical trial that included 250 401 adults, no overall decrease in subsequent spine-related health care utilization after the intervention was observed. However, there was a significant decrease in opioid prescriptions at 1 year in the intervention group compared with the control group.

**Meaning:**

The findings of this study suggest that including epidemiological benchmarks on spinal imaging reports has little impact on subsequent spine-related utilization overall but may reduce subsequent opioid prescriptions.

## Teaser

This randomized clinical trial evaluates the impact of including benchmark prevalence data in routine spinal imaging reports on subsequent spine-related health care utilization and opioid prescriptions.

## Introduction

Spine imaging often reveals incidental findings among individuals without back pain,^1,2^ which can lead to unnecessary and possibly harmful tests and treatments.^3,4^ Roland and van Tulder^5^ proposed adding statements to plain film reports describing the prevalence of degenerative findings in people without back pain. In small observational studies, we and others^6,7^ have found that primary care patients undergoing lumbar spine imaging were less likely to receive certain subsequent diagnostic and therapeutic interventions if imaging reports contained information describing the prevalence of common imaging findings among individuals without back pain. These results suggest that benchmark information may reassure both patients and physicians, resulting in fewer downstream interventions. Since beginning our trial, others have published research suggesting that contextualizing imaging information can affect both health care professionals and patients.^7,8,9^

We now report the results of a large, prospective randomized clinical trial of this intervention, the Lumbar Imaging with Reporting of Epidemiology (LIRE) trial. Our primary hypothesis was that patients of primary care professionals (PCPs) who received lumbar spine imaging reports with age-appropriate and imaging modality–appropriate benchmark prevalence data would have less spine-related health care utilization, as measured by our primary outcome, spine-related relative value units (RVUs) (eAppendix 1 in Supplement 1).^10,11^ RVUs are based on *Current Procedural Terminology* (*CPT*)^12^ and provide a common metric for comparing health care utilization resulting from physician services.^10^ We also report the impact of the intervention on the prespecified secondary outcome of subsequent opioid prescriptions and prespecified subgroup analyses examining initial (index) imaging type and index report findings.

## Methods

### Study Design

We conducted a multicenter, stepped-wedge, cluster randomized clinical trial assigning primary care clinics at 4 large health systems to when they would begin receiving lumbar spine imaging reports containing age-appropriate and modality-appropriate epidemiological benchmarks for common imaging findings. We previously published our study protocol, and it is available in Supplement 2.^13^ We designed LIRE to be highly pragmatic (eAppendix 2 in Supplement 1)^14^ to measure effects in routine care settings. We chose clinic-level cluster randomization because of the strong concern regarding contamination from intervention PCPs to control PCPs. We chose a stepped-wedge randomization because of the appeal of all clusters receiving the intervention by the end of the trial, facilitating implementation and the ability to perform both within-cluster (ie, before and after) and between-cluster comparisons.

Each health care system’s institutional review board or ethics committee reviewed the project, and all institutional review boards classified our study as minimal risk, granting waivers of both informed consent and Health Insurance Portability and Accountability Act authorization. This study followed the Consolidated Standards of Reporting Trials (CONSORT) reporting guideline.

### Participants

We enrolled clinics and their patients at 4 integrated health care systems: Kaiser Permanente Northern California; Henry Ford Health System in Michigan; Kaiser Permanente Washington; and Mayo Clinic Health System in Minnesota and Wisconsin. These systems have comprehensive electronic medical record (EMR) systems to capture health care utilization data.

#### Clinic, PCP, and Patient Eligibility Criteria

At each system, we identified adult primary care clinics and their physicians in family medicine, general internal medicine, and associated mid-level clinicians. We defined a LIRE clinician as a PCP whose main practice was at 1 clinic providing primary care^13^ and who ordered at least 1 qualifying imaging examination during the study period. We enrolled patients aged 18 years and older whose PCP from an eligible clinic ordered an imaging test of the lumbar spine between October 1, 2013, and September 30, 2016. We included all patients receiving eligible imaging studies at participating clinics who had not had lumbar spine imaging within the prior 12 months. We excluded only those patients who had opted out of research studies.

#### Patient Identification

We identified eligible patients and PCPs using the electronic ordering systems. When a PCP ordered an eligible examination, the system automatically determined whether the patient, PCP, and clinic were eligible.

### Randomization

We used a stepped-wedge randomization scheme, randomly assigning clinics in each system to begin receiving the intervention at 1 of 5 dates at 6-month intervals from April 2014 through April 2016. We classified clinics in tertiles by their number of PCPs. The data coordinating center randomly selected clinics using urn-based randomization (without replacement) stratified by system and clinic size stratified by tertile (small, medium, and large). Clinic sizes were represented equally in each randomization wave. Because of the stepped-wedge temporal randomization scheme, we labeled clinics *control* if inclusion of the intervention text had not started and *intervention* after starting inclusion of the intervention text. Masking of the participating clinics was not feasible because of the nature of the intervention. Except for the biostatistician who received and cleaned the data, all investigators at the data coordinating center remained masked to clinic and participant assignment until the final stages of data analysis.

### Procedures

The intervention text consisted of age-specific and modality-specific epidemiological benchmarks indicating the prevalence of common findings from imaging in people without back pain (eAppendix 3 in Supplement 1).^5,6,15^ Using an automated approach through either the radiology information system or the EMR, we inserted the intervention text into lumbar spine imaging reports at intervention clinics. PCPs in control clinics received usual imaging reports.

#### Data and Collection Methods

We collected all data passively from the EMR and electronic administrative data systems. We performed 2 types of data queries from each system. To verify that the systems deployed the intervention appropriately, we queried systems 2 to 4 weeks after the start of each randomization wave for all patients who received an eligible lumbar imaging study. Text matching verified that the reports contained the correct intervention text. One year after the first randomization wave and then every 6 months thereafter, we performed an additional query that included safety and outcome variables. Systems submitted both types of queries as limited data sets (deidentified except for dates of service) to the data coordinating center, providing unique study identifiers for each patient.

We collected diagnosis and utilization data for patients 12 months before index imaging to characterize the cohort at the patient level. Each health system provided prescription data using national drug code or a similar classification from their pharmacy databases. This National Institutes of Health–sponsored trial required the collection of race and ethnicity data, which we obtained through the EMR.

### Outcomes

Our primary outcome was the cumulative spine-related RVUs 365 days after index imaging. Spine-related RVUs are a composite measure of back pain interventions that combine the overall intensity of resource utilization for back pain care in a single metric.^12^ Our summary spine-related RVU incorporated procedures (*CPT* codes), diagnoses (*International Classification of Diseases, Ninth Revision, Clinical Modification* [*ICD-9-CM*]^16^ and *International Statistical Classification of Diseases, Tenth Revision, Clinical Modification* [*ICD-10-CM*]^17^ codes), PCP visits, and inpatient hospitalizations and was based on a validated algorithm.^18^ eAppendix 1 in Supplement 1 provides examples of spine-related procedures and associated RVUs. To obtain a spine-related summary RVU from *CPT* and *ICD-9-CM* and *ICD-10-CM* codes, we used an existing validated algorithm when possible and used a modified version for codes not accounted for by the algorithm.^18,19^ We aggregated relevant *CPT* codes through 1 year after the index imaging test to obtain total spine-related RVUs. The data coordinating center performed all calculations for assessing spine-related RVUs.

We listed the following prespecified secondary outcomes in our published protocol: (1) an indicator of opioid prescribing after the index imaging; (2) cumulative spine-related total RVUs 2 years after index imaging; (3) subsequent advanced imaging (ie, number of magnetic resonance imaging [MRI] or computed tomography [CT] studies) within 90 days and 12 months after index imaging study; (4) spine injections and spine surgeries; and (5) other back-related medical costs during 2 years. In our original study protocol, the opioid outcome was the number of patients with a subsequent opioid prescription written by a study PCP. However, following discussions among the study team, we concluded that the total morphine equivalent dose (MED) prescribed per patient would be a better metric and thus included the number of MEDs prescribed per patient as the opioid outcome in our protocol paper. However, we were unable to obtain the necessary data for this detailed calculation. Instead, we analyzed whether patients had received an opioid prescription from a LIRE PCP within 1 year of index imaging; this was the outcome in our pilot project.^6^ We also report whether an opioid prescription was received within 90 days of index imaging, an outcome not prespecified on ClinicalTrials.gov.

#### Extracting Imaging Results

We used machine learning natural language processing to extract imaging findings from radiology text reports.^20^ We identified common imaging findings that are likely less clinically important (eg, disc bulge, disc space narrowing) vs likely more important (eg, moderate to severe spinal canal stenosis, nerve root compression (eAppendix 4 in Supplement 1).^1,21^

### Statistical Analysis

To evaluate the impact of the intervention, we used multilevel linear mixed-effects models or generalized linear mixed models that cluster on clinic and then PCP within clinic, coupled with the use of robust standard errors for all primary and secondary outcome measures (Supplement 2). In secondary analyses, we used generalized estimating equations, adopting simple exchangeable correlation models at the clinic level to determine whether conclusions were sensitive to model specification (eAppendix 5 in Supplement 1). All analyses used the intention-to-treat principle.^13^

We used a log transformation of RVU [log(RVU + 1)] in primary outcome models to address right skew of the utilization data. A constant (ie, 1) was added to RVU prior to transformation so that participants with 0 RVUs could be included in the analyses. We conducted sensitivity analyses that varied the constant added to RVU before the transformation (eAppendix 5 in Supplement 1). We also constructed a model examining subsequent RVUs in a subgroup of patients from clinics in which patients were less likely to have sought outside care who had utilization within their system through at least 12 months to address the issue of care received out of system.

We used a similar analytic approach for opioid prescriptions as we used for spine-related RVUs but adapted generalized linear models to use logistic regression for this binary outcome. Realizing the potential importance of confounding due to secular trends in opioid prescribing, we conducted additional post hoc opioid analyses exploring sensitivity to alternative modeling of time. We also performed analyses on an outcome that incorporated opioid prescriptions from both LIRE and non-LIRE PCPs.

We had 2 prespecified subgroup analyses.^13^ We used findings extracted from the reports to determine whether the findings in the imaging report influenced the effects of the intervention. We also examined whether the intervention effect was modified by modality of index imaging. We tested these hypotheses as interaction terms using the Wald test.

A post hoc subgroup analysis distinguished between those patients who were and were not prior opioid users because the intervention might have been more likely to prevent opioid prescriptions from being written for opioid-naive patients. We defined prior use as at least 1 opioid prescription written within 120 days before the index imaging date, and we included an interaction term of prior opioid prescription status with intervention status in those models.^22^ We used model results and patient covariates to calculate predicted RVUs and predicted probability of opioid prescription for each participant under both the control and intervention conditions and aggregated the results to report median adjusted RVUs and adjusted opioid prescriptions by intervention status and subgroups.

We used SAS software version 9.4 (SAS Institute) for all analyses. Statistical significance was set at *P* < .05, and all tests were 2-tailed.

#### Power for Primary Outcome

We calculated statistical power for the primary outcome, spine-related RVUs.^13^ The study had 89% power to detect reductions of 5.0% or greater.

#### Data Safety Monitoring

Two external safety officers monitored emergency department visits within 90 days and deaths within 6 months of index imaging. The safety officers used absolute relative risk ratio monitoring thresholds of 1.15 and 1.10 for comparing 90-day emergency department visit and death rates by intervention group, with adjustment for patient-specific characteristics (ie, age, sex, Charlson comorbidity index^23^), health care system, image modality, time, season, and clinic size.^24^

## Results

**Figure 1.**
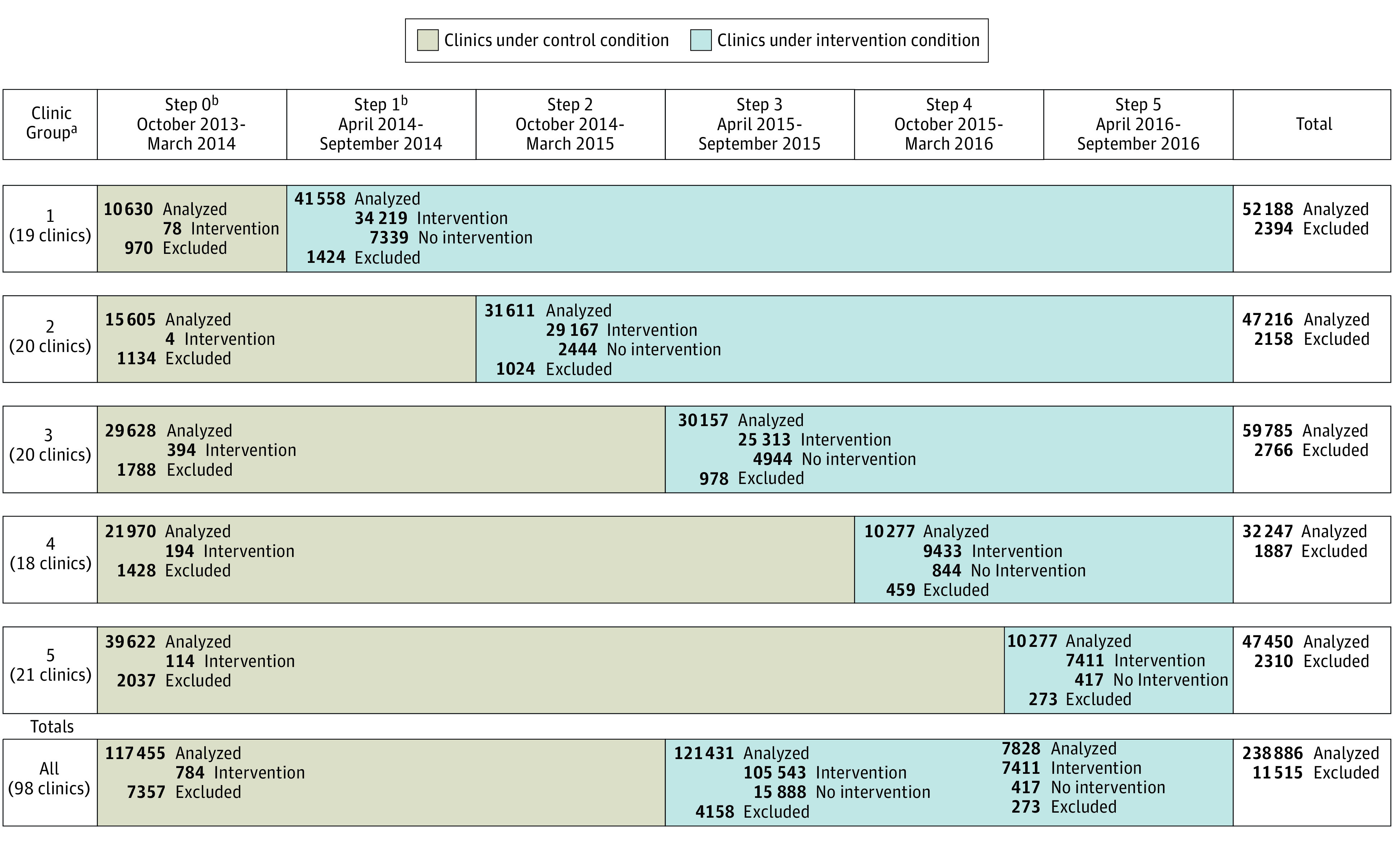
CONSORT Stepped-Wedge Allocation of Trial Subjects For clinics under the control condition, intervention indicates the intervention text was mistakenly included in the image report. For clinics under the intervention condition, intervention indicates that the intervention text was successfully included in the image report and no intervention indicates that the intervention text was not included. ^a^Two small clinics randomized to groups 2 and 5 were dropped before the first data submission because of clinic closure and are not included in the clinic counts. ^b^By pretrial design, for 1 clinic, step 0 extended through May 2014, and step 1 began June 1, 2014.

We randomly allocated intervention start dates to 98 clinics with 3278 PCPs and 250 401 patients. A total of 11 515 patients were excluded for the following reasons: prior lumbar spine image within 12 months (11 149 [96.8%]), imaging report finalization date more than 4 days after image completion date (354 [3.1%]), image completion date prior to report finalization date (3 [<0.1%]), and unable to link to utilization data (9 [0.1%]). This resulted in a final sample of 238 886 patients (95.4%; 137 373 [57.5%] women; 105 497 [44.2%] aged >60 years) with 3257 PCPs (99.4%). Three health systems were of comparable size and enrolled 41 882 patients (17.5%) from 936 PCPs (28.7%) while the fourth health system enrolled 197 004 patients (82.5%) from 2321 PCPs (71.3%) (Figure 1). We did not observe any substantial differences in the baseline characteristics between the control and intervention groups (Table).

**Table.** Baseline Characteristics

Characteristic	No. (%)	
	Control (n = 117 455)	Intervention (n = 121 431)
Site		
A	6950 (5.9)	7388 (6.1)
B	96 275 (82.0)	100 729 (83.0)
C	7846 (6.7)	7736 (6.4)
D	6384 (5.4)	5588 (4.6)
Age, y		
18-39	21 237 (18.1)	22 105 (18.2)
40-60	45 032 (38.3)	44 995 (37.1)
≥61	51 186 (43.6)	54 331 (44.7)
Sex^a^		
Women	67 915 (57.8)	69 458 (57.2)
Men	49 534 (42.2)	51 965 (42.8)
Race		
American Indian or Alaska Native	806 (0.7)	880 (0.7)
Asian	13 311 (11.3)	13 197 (10.9)
Black or African American	11 919 (10.1)	11 649 (9.6)
Native Hawaiian or other Pacific Islander	905 (0.8)	709 (0.6)
White	76 431 (65.1)	79 142 (65.2)
Multiracial or other	459 (0.4)	546 (0.4)
Unknown or not reported	13 624 (11.6)	15 308 (12.6)
Ethnicity		
Hispanic or Latino	17 754 (15.1)	18 475 (15.2)
Not Hispanic or Latino	19 867 (16.9)	19 276 (15.9)
Not available^b^	79 834 (68.0)	83 680 (68.9)
Modality		
RG	93 465 (79.6)	98 970 (81.5)
CT	494 (0.4)	449 (0.4)
MR	23 496 (20)	22 012 (18.1)
Charlson Comorbidity Index		
0	75 106 (63.9)	77 973 (64.2)
1	20 675 (17.6)	21 193 (17.5)
2	11 451 (9.7)	11 760 (9.7)
≥3	10 223 (8.7)	10 505 (8.7)
Finding status		
None	27 770 (23.6)	27 776 (22.9)
LIRE finding without clinically important finding	72 127 (61.4)	77 065 (63.5)
Clinically important finding	17 558 (14.9)	16 590 (13.7)
≥1 Opioid prescriptions prior to index	32 225 (27.4)	29 306 (24.1)
Primary insurance at index		
Medicare	44 362 (37.8)	46 479 (38.3)
Medicaid or state-subsidized	5546 (4.7)	6510 (5.4)
Commercial	65 375 (55.7)	66 368 (54.7)
VA	117 (0.1)	131 (0.1)
Self-pay	731 (0.6)	570 (0.5)
Unknown or not reported	1324 (1.1)	1373 (1.1)
Socioeconomic index, mean (SD)^c^	57 (6)	57 (7)
Health care professional type		
MD	105 359 (89.7)	108 165 (89.1)
DO	8131 (6.9)	9157 (7.5)
Extender, eg, NP, PA	3965 (3.4)	4109 (3.4)
Health care professional specialty		
Family medicine	56 795 (48.4)	60 277 (49.6)
Internal medicine	59 684 (50.8)	60 158 (49.5)
Other	976 (0.8)	996 (0.8)
Female health care professional	62 840 (53.5)	62 680 (51.6)
Health care professional age, mean (SD), y^d^	49 (9)	49 (9)

Abbreviations: CT, computed tomography; DO, doctor of osteopathy; MD, medical doctor; LIRE, Lumbar Imaging with Reporting of Epidemiology; MR, magnetic resonance; NP, nurse practitioner; PA, physician’s assistant; RG, radiograph; VA, Veterans Administration.

^a^ Does not include 14 patients (<0.1%) with other or unknown gender.

^b^ Due to the manner in which race and ethnicity are collected at 1 health system (ie, sometimes the concepts are conflated and sometimes Hispanic ethnicity is captured by a single checkbox), it is not possible to reliably distinguish between “not Hispanic” and “did not answer.”

^c^ Does not include 6810 patients (2.7%) with unknown socioeconomic index. Sites mapped participant addresses to Federal Information Processing System codes at the block-group level using geocoding software. These codes were mapped to socioeconomic indices derived from data available from the 2010 Census Summary File 1 and the American Community Survey, 2007 to 2011, 5-year estimate data.

^d^ Does not include 424 patients (0.1%) for whom provider age was unknown.

**Figure 2.**
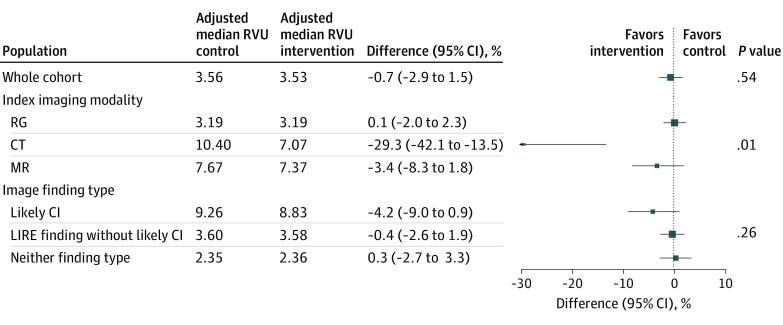
Model Results for Spine-Related Relative Value Units (RVUs) at 1 Year All models adjust for health system, clinic size, age range (ie, 18-39, 40-60, and ≥61 years), sex, imaging modality, Charlson Comorbidity Index category (ie, 0, 1, 2, and ≥3), and health system specific time trends. Models include hierarchical random effects for clinic (intercept and treatment) and primary care professional (intercept only). *P* values for subgroup models (ie, index imaging type and image finding type) are for Wald tests for effect modification. CI indicates clinically important, CT, computed tomography; RG, radiograph; and MR, magnetic resonance.

Our primary outcome, 12-month spine-related RVU, was not significantly different for the intervention group compared with the control group (adjusted median [interquartile range], 3.53 [2.68-5.08] vs 3.56 [2.71-5.12]; difference, −0.7%; 95% CI, −2.9% to 1.5%; *P* = .54) (Figure 2). Injections and surgery accounted for a higher proportion of subsequent spine-related RVUs for patients who had magnetic resonance imaging or computed tomography for their index examination compared with radiographs, while physical therapy and imaging were proportionally higher for patients who had radiographs as the index imaging test (eAppendix 7 in Supplement 1).

**Figure 3.**
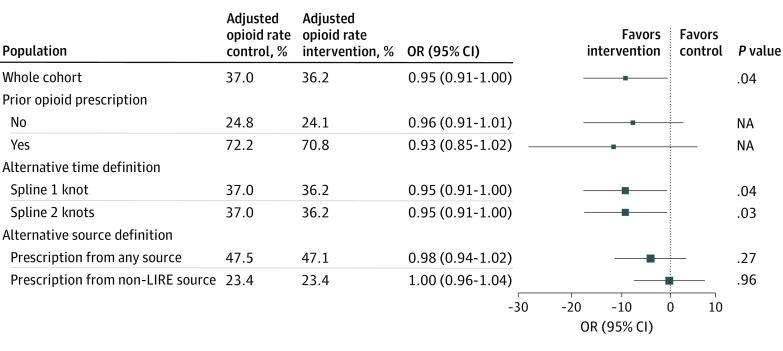
Model Results for Opioid Prescriptions Within 12 months All models adjust for health system, clinic size, age range (ie, 18-39, 40-60, and ≥61 years), sex, imaging modality, Charlson Comorbidity Index category (ie, 0, 1, 2, and ≥3), prior opioid use, and health system specific time trends. Models include hierarchical random effects for clinic (intercept and treatment) and primary care professional (intercept only). Prior opioid prescription is defined as having 1 or more prescriptions in the 120 days prior to index imaging. A Lumbar Imaging with Reporting of Epidemiology (LIRE) source is any health care professional who ordered an index lumbar spine image for 1 or more participants in the LIRE trial. It need not be the same individual who ordered the patient’s index image. A *non-LIRE source *is any other health care professional. *Any source *includes both LIRE and non-LIRE clinicians. NA indicates not applicable.

**Figure 4.**
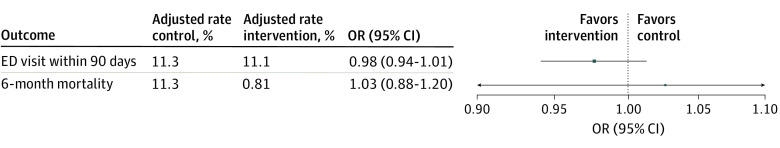
Safety Outcomes All models adjust for health system, clinic size, age range (ie, 18-39, 40-60, and ≥61 years), sex, imaging modality, Charlson Comorbidity Index category (ie, 0, 1, 2, and ≥3), seasonality, and health system specific time trends. The emergency department (ED) visit model includes hierarchical random effects for clinic (intercept and treatment) and primary care professional (intercept only). The mortality model uses general estimating equations with clustering on clinic.

Our prespecified secondary outcome, opioid prescriptions by a LIRE PCP within 1 year of index imaging, demonstrated a small but statistically significant reduction in the odds of receiving at least 1 prescription for an opioid for patients in the intervention group compared with patients in the control group (adjusted opioid proportion, 36.2% vs 37.0%; odds ratio, 0.95; 95% CI, 0.91-1.00; *P* = .04) (Figure 3). Sensitivity analyses with alternative modeling of time yielded similar results (Figure 3). Comparison of opioid prescribing between control and intervention groups within 90 days following index imaging showed a similar small reduction in the odds of receiving an opioid prescription for the intervention group compared with the control group (adjusted opioid proportion, 28.9% vs 29.8%; odds ratio, 0.95; 95% CI, 0.90-0.99; *P* = .02) (eAppendix 6 in Supplement 1). Safety monitoring demonstrated no evidence of increased deaths or emergency department visits in the intervention vs control group within 6 months of the index test (adjusted emergency department visit rate, 11.1% vs 11.3%; OR, 0.98; 95% CI, 0.94-1.01) (Figure 4).

The prespecified subgroup analysis of whether the intervention differentially affected spine-related RVUs by imaging modality revealed that the small number of patients (943 [0.4%]) of patients who had computed tomography as the index imaging had markedly lower subsequent median RVUs if exposed to the intervention (mean difference, −29.3%; 95% CI,−42.1% to −13.5%). The nearly 20% of patients (45 508 [19.1%]) who had magnetic resonance imaging had lower subsequent RVUs in the intervention group (difference, −3.4%; 95% CI, −8.3% to 1.8%), although this was not statistically significant (Figure 2). The second prespecified subgroup analysis that examined whether image finding type differentially affected spine-related RVUs revealed no differences in subsequent median RVUs in the intervention compared with the control group (Figure 2).

In a post hoc subgroup analysis, the adjusted proportion of control patients without a prior opioid prescription who received an opioid prescription from a LIRE PCP within 1 year following index imaging was 25% compared with 72% for control patients with a prior opioid prescription. However, there was no intervention effect modification by prior opioid prescription status (test for effect modification, *P* = .58) (Figure 3). When we included prescriptions from non-LIRE PCPs who were not exposed to the intervention in the 1-year opioid outcome, the intervention effect was attenuated (adjusted opioid proportion, 47.1% intervention vs 47.5% control; OR, 0.98; 95% CI, 0.94-1.02; *P* = .27) (Figure 3).

## Discussion

The LIRE intervention did not reduce subsequent spine-related RVUs for the population as a whole. However, patients in the intervention group were less likely (OR, 0.95; 95% CI, 0.90-0.99; *P* = .02) to receive a subsequent opioid prescription compared with patients not receiving the intervention. The intervention also reduced subsequent spine-related RVUs for the small proportion of patients with CT as the index imaging.

Pragmatic trials must be simple to implement and the populations relatively unselected. Thus, a negative primary result is not unusual.^25,26,27^ This suggests the likely importance of heterogeneous intervention effects, prespecified subgroup analyses, and prespecified secondary outcomes.

An explanation for the differential effect by imaging modality is that patients undergoing CT for their index imaging were more likely to receive back pain interventions than patients receiving other modalities, and thus, the intervention was more effective at reducing subsequent interventions in patients who were most likely to receive those interventions in the first place (eAppendix 7 in Supplement 1).

Our finding of no greater subsequent emergency department visits and deaths in the intervention group provides reassurance that the intervention did not cause deleterious undertreatment. Given the climate of overdiagnosis and overtreatment of back pain in the United States, undertreatment may be less likely to occur in the United States than elsewhere. Our intervention provided an opportunity to increase the knowledge of patients and health care professionals. Because we did not detect any harm of the intervention and we did detect a possible benefit, including the intervention should safely allow patients and health care professionals to make better informed decisions.

Finally, our primary null result may have been different if we had studied different health systems. For example, if we had enrolled clinics with higher baseline utilization of tests for back pain patients, we may have found a positive result.

### Limitations

This study has limitations. Opioid prescribing decreased in the United States during our study.^28^ Although we made multiple efforts to account for this potential confounding in our modeling, residual confounding may exist.

Because we did not collect patient-reported outcomes, we cannot comment on outcomes such as functional status, pain, or psychosocial functioning. The decision not to collect patient-reported data was deliberate, based on the recognition that it could jeopardize the feasibility of this large pragmatic trial of more than 250 000 patients.

We also did not capture patient care not included in the EMRs. However, we found similar results to those of our primary analysis when we examined subsequent RVUs from patients less likely to seek outside care (eAppendix 5 in Supplement 1). Previous studies have shown high degrees of accuracy when EMR data were validated by manual medical record reviews.^29^

All of our participating health systems were integrated delivery systems and nonprofit. There is evidence that nonprofit hospitals may be less responsive to the type of intervention that we tested than for-profit hospitals.^30^ However, this conservative bias emphasizes the robustness of the positive impact that we observed with respect to opioid prescribing. Our findings may also not be generalizable to systems having greater restrictions on advanced imaging. We do not know the indication for imaging, including whether the patient had a red flag, so we cannot comment on the appropriateness.

## Conclusions

In this study, adding benchmark prevalence information for spine imaging findings did not reduce subsequent spine-related RVUs, but it slightly reduced the likelihood of subsequent opioid prescribing, an important prespecified secondary outcome. Reporting benchmark information is a fundamental change to the imaging reporting paradigm that may be relevant for other conditions and could easily be applied to other diagnostic tests (eg, other imaging tests, genetic testing). Finally, unmeasured benefits of the intervention may result from patients and health care professionals having a better understanding of the clinical meaning of imaging findings.

## Notes

Supplement 1. **eAppendix 1.** Outcome Sources and Definitions **eAppendix 2.** PRECIS-2 Diagram **eAppendix 3.** Intervention Text **eAppendix 4.** Imaging Findings **eAppendix 5.** Sensitivity Analyses for Spine-Related Relative Value Units (RVUs) Outcome **eAppendix 6.** Opioid Prescription Within 90 Days **eAppendix 7.** Patient Characteristics and Outcomes by Index Modality **eReferences.**

Supplement 2. **Trial Protocol and Statistical Analysis Plan**

Supplement 3. **Data Sharing Statement**
