# Supplementary material for: The Effect of Including Benchmark Prevalence Data of Common Imaging Findings in Spine Image Reports on Health Care Utilization Among Adults Undergoing Spine Imaging: A Stepped-Wedge Randomized Clinical Trial
Source: JAMA Netw Open. 2020 Sep 4;3(9):e2015713. doi: 10.1001/jamanetworkopen.2020.15713 (PMC7489827; doi:10.1001/jamanetworkopen.2020.15713)
Supplement: Supplement 1. — eAppendix 1. Outcome Sources and Definitions eAppendix 2. PRECIS-2 Diagram eAppendix 3. Intervention Text eAppendix 4. Imaging Findings eAppendix 5. Sensitivity Analyses for Spine-Related Relative Value Units (RVUs) Outcome eAppendix 6. Opioid Prescription Within 90 Days eAppendix 7. Patient Characteristics and Outcomes by Index Modality eReferences. [file jamanetwopen-e2015713-s001.pdf]

Supplementary Online Content

Jarvik JG, Meier EN, James KT, et al. The effect of including benchmark prevalence data of common imaging findings in spine image reports on health care utilization among adults undergoing spine imaging: a stepped-wedge randomized clinical trial. *JAMA Netw Open*. 2020;3(9):e2015713. doi:10.1001/jamanetworkopen.2020.15713

eAppendix 1. Outcome Sources and Definitions
eAppendix 2. PRECIS-2 Diagram
eAppendix 3. Intervention Text
eAppendix 4. Imaging Findings
eAppendix 5. Sensitivity Analyses for Spine-Related Relative Value Units (RVUs) Outcome
eAppendix 6. Opioid Prescription Within 90 Days
eAppendix 7. Patient Characteristics and Outcomes by Index Modality
eReferences.

This supplementary material has been provided by the authors to give readers additional information about their work.

© 2020 Jarvik JG et al. *JAMA Network Open.*

eAppendix 1. Outcome Sources and Definitions

eTable 1. Outcomes data from EMR

Domain	Specific Element
Hospitalizations (all inpatient stays)	• Duration (days)<br>• Diagnosis related group (DRG)<br>• Associated Current Procedural Terminology (CPT) and International Classification of Diseases-9 or 10 (ICD-9 or 10) codes
Outpatient visits	• Clinic type<br>• Associated ICD-9 or 10 codes
Pharmacy data (prescribed medications)	• National Drug Code<br>• Drug name, dose, quantity, days' supply
Procedures (inpatient and outpatient, including subsequent imaging)	• Procedure type and indication<br>• Associated CPT and ICD-9 or 10 codes
Safety Data	• ER visits (within 90 days of index image)<br>• Death (within 6 months of index image)

Calculating spine-related relative value units (RVUs)

We calculated a patient's summary measure of spine-related RVUs by summing the spine-related RVUs of inpatient and outpatient encounters in the year following index imaging. Each spine-related procedure in an encounter contributed to the encounter RVUs. We included both the work and practice RVU components in our measure.

We then had at least two physicians with longstanding clinical expertise in spine care (JGJ, JRF, PS, RAD) assign all procedures in the LIRE database to one of three categories: **definitely spine-related**, **possibly spine-related**, or **not spine-related**. We resolved disagreements between the physicians through consensus. An example of a **definitely spine-related** procedure is a surgical fusion of the lumbar spine. An example of a **possibly spine-related** procedure is a physical therapy visit. We used diagnosis codes associated with an encounter to determine whether a **possibly spine-related** procedure in that encounter should be included in the spine-related RVU. We categorized all International Classification of Diseases (ICD) codes in the LIRE database as spine-related or not. If we assigned a spine-related diagnosis code to an encounter, then all **possibly spine-related** procedures in the encounter contributed to the encounter's spine-related RVU. Similarly, if a **definitely spine-related** procedure was associated with an encounter, all **possibly spine-related** procedures in the encounter contributed to spine-related RVU.

We further categorized all **definitely spine-related** and **possibly spine-related** procedures by procedure type: manual (e.g. physical therapy), evaluation and management, injection, imaging, or surgery. If we determined that a procedure in an encounter was a spine-related surgery, the RVU associated with all procedures in the encounter, regardless of spine-relatedness categorization, contributed to the spine-related RVU for the encounter. Our guiding principle was that all procedures during the encounter were likely to be related to the spine surgery performed.

eFigure 1 illustrates the spine-related RVU inclusion algorithm for an encounter. eTable 2 presents RVU values for example spine-related procedures.

© 2020 Jarvik JG et al. *JAMA Network Open.*

eFigure 1. Assigning spine-related RVUs

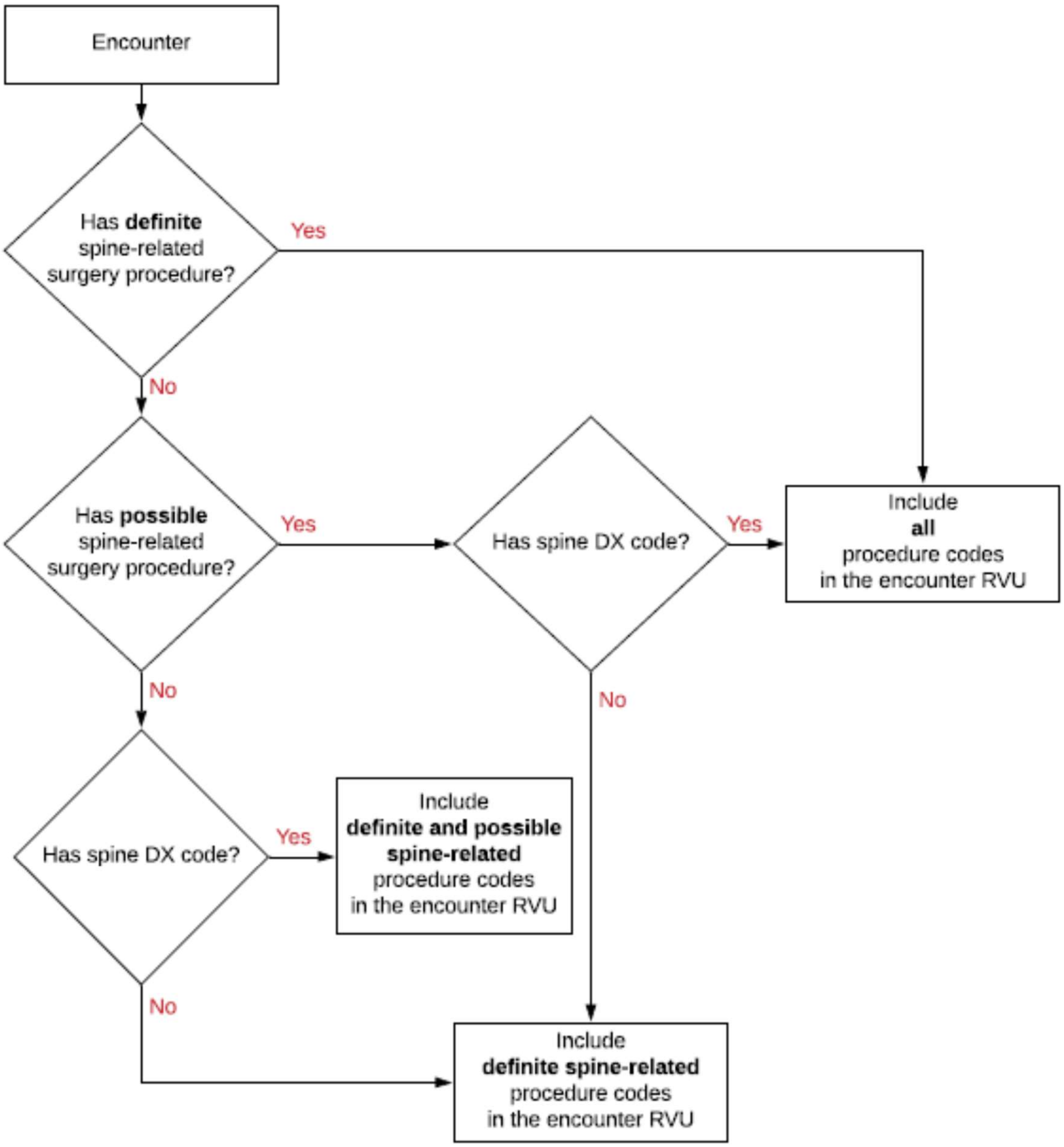

All International Classification of Diseases (ICD) diagnosis codes were categorized as spine-related or not. An encounter with one or more diagnosis codes categorized as spine-related is defined as having a “spine DX code”. All procedures were categorized as definitely spine-related, possibly spine-related, or not spine-related. Procedures were also categorized as manual (e.g. physical therapy), evaluation and management, injection, imaging, or surgery.

© 2020 Jarvik JG et al. *JAMA Network Open.*

eTable 2. Examples of spine-related CPT codes and associated RVUs		
**CPT Code**	**Description**	**RVU[a]**
72100	X-ray exam of lower spine – 2 or 3 views	0.99
97001	Physical Therapy Evaluation	2.18
99214	Detailed office visit	3.03
99284	Emergency department visit – high intensity	3.32
64483	Epidural injection for lumbar spinal stenosis	6.26
72131	CT lumbar spine without contrast	5.09
72148	MRI Lumbar Spine without contrast	6.37
63047	Removal of spinal lamina (laminectomy)	32.13
22804	Fusion of the spine	70.65
a. Relative value units (RVUs) include work and practice components. RVUs may change over time. The median value over the study period was assigned to each CPT code to avoid any potential time-related bias.		

Opioid prescription outcomes

The 1-year opioid outcome is a binary indicator of whether one or more outpatient opioid prescriptions were written for the patient by a *LIRE provider* within 1 year of index imaging. A *LIRE provider* is defined as any provider who ordered one or more index images for the trial. The provider need not be the provider who ordered the patient's index image.

All unique drug names in the LIRE database were categorized as being an opioid or not. eTable 3 shows the drug names that were included in the opioid list.

eTable 3. Drug names included in opioid definition
Buprenorphine-containing products
Butorphanol
Codeine-containing products
Fentanyl
Hydrocodone-containing products
Hydromorphone
Meperidine
Methadone
Morphine
Nalbuphine
Oxycodone-containing products
Oxymorphone
Pentazocine-containing products
Propoxyphene
Sufentanil
Tapentadol
Tramadol-containing products
Note: The medications above have at least one non-intravenous (IV) dosage form available, so could be used in outpatient setting.

© 2020 Jarvik JG et al. *JAMA Network Open.*

eAppendix 2. PRECIS-2 Diagram

eFigure 2. Pragmatic explanatory continuum indicator summary (PRECIS-2*)[1]

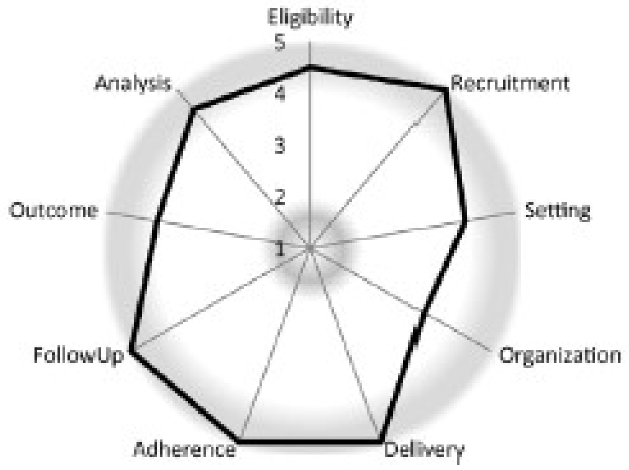

* Independent researchers with experience conducting clinical trials assigned the LIRE PRECIS rating**. [2]**

© 2020 Jarvik JG et al. *JAMA Network Open.*

eAppendix 3. Intervention Text

**eTable 4. Examples of intervention text**
Modality: X-ray Age: 40-60<br>The following findings are so common in normal, pain-free volunteers that while we report their presence, they must be interpreted with caution and in the context of the clinical situation. Among people between the age of 40 and 60 years who do not have back pain, a plain film, x-ray will find that about:<br><br>8 in 10 have disk degeneration<br>6 in 10 have disk height loss<br><br>Note that even 3 in 10 means that the finding is quite common in people without back pain.
Modality: MRI Age: 61+<br>The following findings are so common in normal, pain-free volunteers that while we report their presence, they must be interpreted with caution and in the context of the clinical situation. Among people over the age of 60 who do not have back pain, an MRI will find that about:<br><br>9 in 10 have disk degeneration<br>9 in 10 have disk signal loss (desiccation)<br>8 in 10 have disk height loss<br>8 in 10 have a bulging disk<br>4 in 10 have an annular fissure<br>4 in 10 have a disk protrusion<br>4 in 10 have facet degeneration<br>3 in 10 have spondylolisthesis<br><br>Note that even 3 in 10 means that the finding is quite common in people without back pain.

eTable 5. Intervention implementation details

**Feature**	**System A**	**System B**	**System C**	**System D**
Informatics system used to insert intervention text	EMR	RIS	EMR	EMR
Intervention text location	Pop-up alert	End of report	End of report	End of report
Intervention text insertion timing	When report viewed by referring provider	At time of dictation	After report finalization by radiologist	After report finalization by radiologist
Intervention text could be modified/removed by radiologist	No	Yes	No	No

Abbreviations: EMR – electronic medical record; RIS – radiology information system

eAppendix 4. Imaging Findings

**eTable 6. Imaging findings likely clinically important vs. not likely clinically important**	
**Likely clinically important[a]**	**Not likely clinically important[a]**
• Moderate or severe stenosis[b]	• Annular Fissure
• Disc extrusion	• Disc height loss
• Nerve root displacement or compression	• Mild stenosis (central, lateral recess or foraminal)
• Endplate edema (Type 1 endplate change)	• Nerve root contact without displacement/compression
• Grade 2 or higher listhesis	• Grade 1 listhesis
	• Disc desiccation
	• Disc bulge
	• Disc protrusion
	• Facet degeneration (any severity)

a. Note that none of the health systems used structured reporting and findings were derived using natural language processing (NLP) techniques.
b. Central, lateral recess or foraminal.

**eTable 7. RVU at one year by image finding type**		
**Image finding type**	**Median (IQR)**	**Mean (SD)**
Likely clinically important	10.1 (3.2, 23.8)	22.9 (42.2)
LIRE finding w/o likely clinically important finding	3.3 (0, 12.6)	11.7 (28.0)
Neither finding type	2.1 (0, 8.4)	8.0 (20.7)

Abbreviations: RVU – relative value unit; IQR – interquartile range; SD – standard deviation

© 2020 Jarvik JG et al. *JAMA Network Open.*

eAppendix 5. Sensitivity Analyses for Spine-Related Relative Value Units (RVUs) Outcome

eFigure 3. Spine-related RVUs at 1 year modeled with general estimating equations (GEE)

Population	Adjusted Median RVU Control	Adjusted Median RVU Intervention	% Diff (95% CI)	
Whole cohort	3.38	3.34	−0.9 (−3.0, 1.3)	p=0.42
Index imaging modality				*p=0.02*
XR	3.20	3.19	0.0 (−2.1, 2.1)	
CT	10.37	7.22	−27.7 (−41.0, −11.4)	
MR	7.86	7.53	−3.8 (−8.8, 1.4)	
Image finding type				*p=0.24*
Likely clinically important (CI)	9.31	8.85	−4.5 (−9.4, 0.6)	
LIRE finding w/o likely CI	3.53	3.51	−0.5 (−2.7, 1.7)	
Neither finding type	2.29	2.29	0.2 (−2.8, 3.3)	

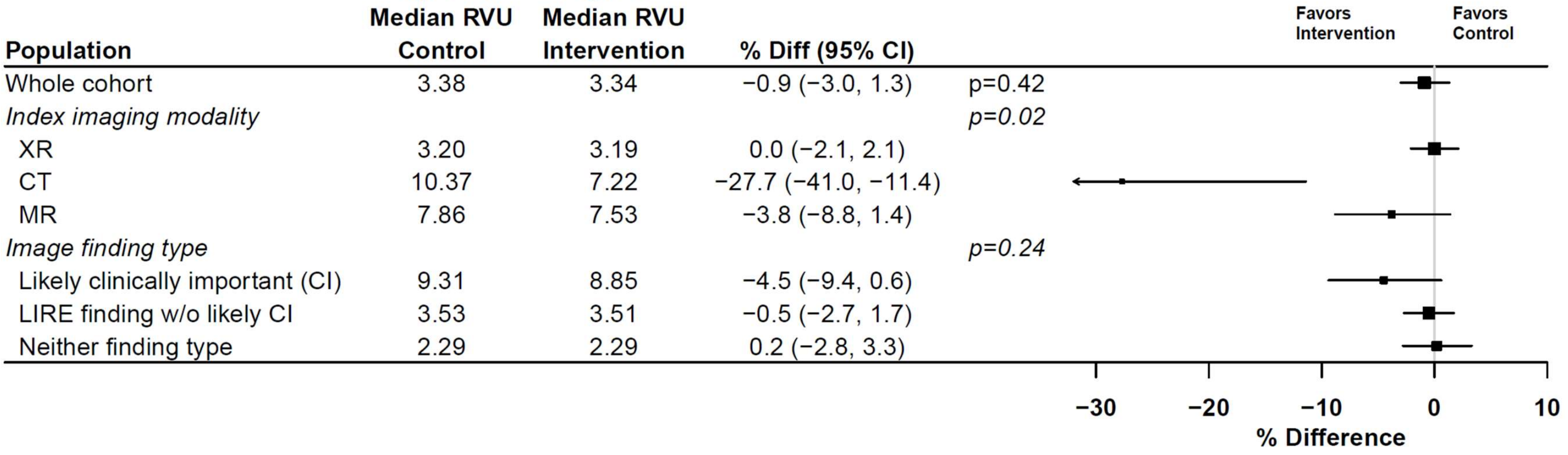

All models adjust for health system, clinic size, age range (18-39, 40-60, 61+), gender, imaging modality, Charlson Comorbidity Index category (0, 1, 2, 3+), and health system specific time trends and include hierarchical random effects for clinic (intercept and treatment) and provider (intercept only). P values for subgroup models (index imaging type and image finding type) are for Wald tests for effect modification.

Abbreviations: RVU – relative value unit; 95% CI – 95% confidence interval; XR – x-ray; CT – computed tomography; MR – magnetic resonance

© 2020 Jarvik JG et al. *JAMA Network Open.*

eFigure 4. Spine-related RVUs at 1 year modeled as log (RVU + 0.5)

Population	Adjusted Median RVU Control	Adjusted Median RVU Intervention	% Diff (95% CI)	
Whole cohort	2.90	2.87	−0.9 (−3.5, 1.7)	p=0.49
Index imaging modality				*p=0.009*
XR	2.58	2.57	0.0 (−2.6, 2.6)	
CT	9.50	6.12	−33.8 (−47.6, −16.5)	
MR	6.84	6.55	−3.9 (−9.6, 2.1)	
Image finding type				*p=0.27*
Likely clinically important (CI)	8.45	8.01	−5.0 (−10.6, 0.9)	
LIRE finding w/o likely CI	2.95	2.93	−0.5 (−3.2, 2.2)	
Neither finding type	1.85	1.86	0.2 (−3.4, 3.9)	

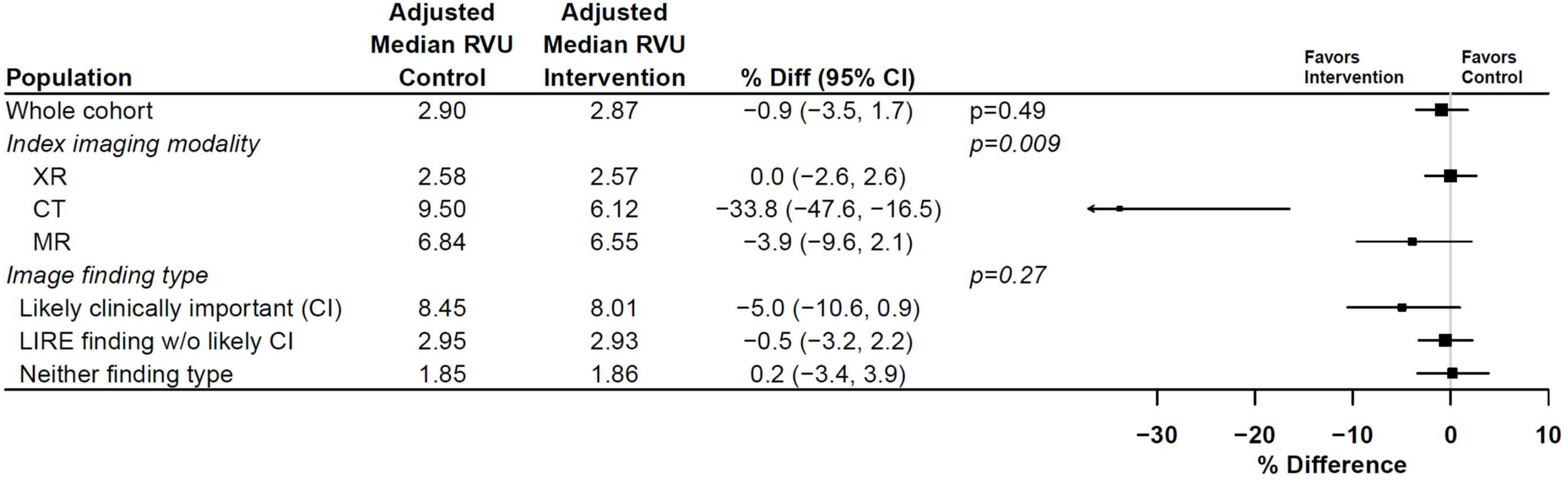

All models adjust for health system, clinic size, age range (18-39, 40-60, 61+), gender, imaging modality, Charlson Comorbidity Index category (0, 1, 2, 3+), and health system specific time trends and include hierarchical random effects for clinic (intercept and treatment) and provider (intercept only). P values for subgroup models (index imaging type and image finding type) are for Wald tests for effect modification.

Abbreviations: RVU – relative value unit; 95% CI – 95% confidence interval; XR – x-ray; CT – computed tomography; MR – magnetic resonance

© 2020 Jarvik JG et al. *JAMA Network Open.*

eFigure 5. Spine-related RVUs at 1 year for subgroup known to have full year of health system coverage

Population	Adjusted Median RVU Control	Adjusted Median RVU Intervention	% Diff (95% CI)	
Whole cohort	3.54	3.50	−0.9 (−3.5, 1.8)	p=0.53
Index imaging modality				*p=0.004*
XR	3.18	3.19	0.1 (−2.3, 2.7)	
CT	10.61	6.98	−31.3 (−44.5, −15.0)	
MR	7.65	7.27	−4.4 (−9.9, 1.5)	
Image finding type				*p=0.21*
Likely clinically important (CI)	9.23	8.73	−4.8 (−10.1, 0.8)	
LIRE finding w/o likely CI	3.57	3.55	−0.5 (−3.2, 2.2)	
Neither finding type	2.36	2.38	0.6 (−2.6, 4.0)	

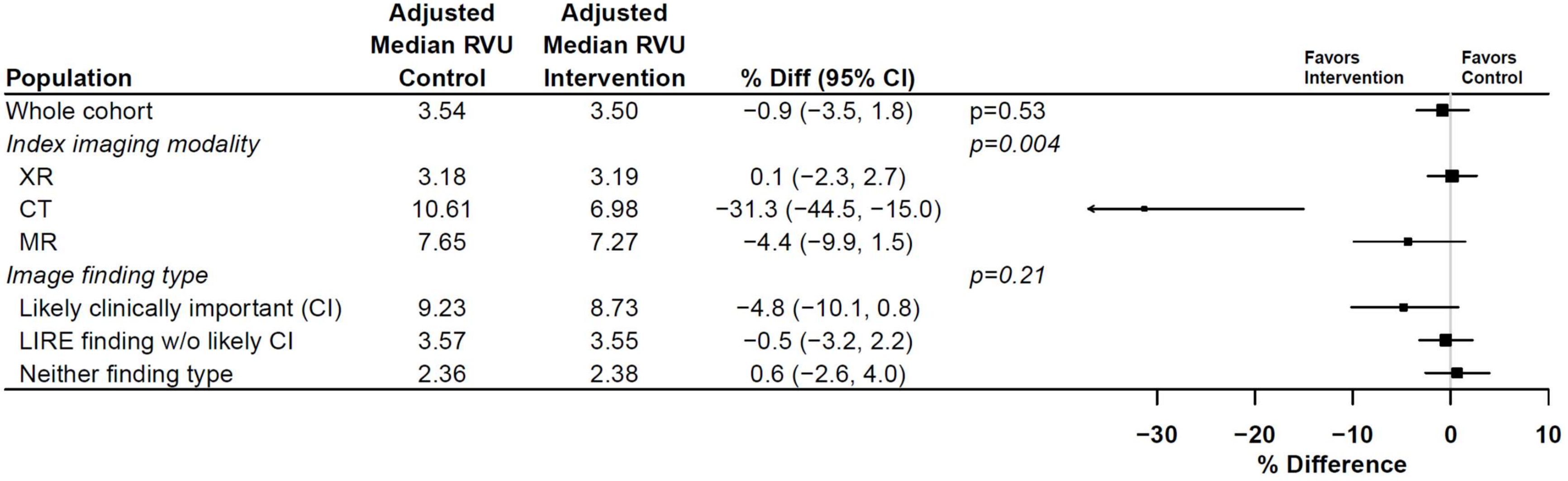

All models adjust for health system, clinic size, age range (18-39, 40-60, 61+), gender, imaging modality, Charlson Comorbidity Index category (0, 1, 2, 3+), and health system specific time trends and include hierarchical random effects for clinic (intercept and treatment) and provider (intercept only). P values for subgroup models (index imaging type and image finding type) are for Wald tests for effect modification.

Abbreviations: RVU – relative value unit; 95% CI – 95% confidence interval; XR – x-ray; CT – computed tomography; MR – magnetic resonance

© 2020 Jarvik JG et al. *JAMA Network Open.*

eFigure 6. Mean Spine-related RVUs at 1 year

Population	Adjusted Mean RVU Control	Adjusted Mean RVU Intervention	Diff (95% CI)	
Whole cohort	12.45	12.40	−0.06 (−0.50, 0.39)	p=0.80
Index imaging modality				*p=0.11*
XR	10.51	10.64	0.13 (−0.30, 0.57)	
CT	24.57	20.15	−4.42 (−9.50, 0.66)	
MR	20.37	19.62	−0.75 (−1.96, 0.46)	
Image finding type				*p=0.68*
Likely clinically important (CI)	23.14	22.57	−0.57 (−1.97, 0.83)	
LIRE finding w/o likely CI	11.69	11.69	−0.00 (−0.46, 0.45)	
Neither finding type	7.94	8.02	0.08 (−0.39, 0.55)	

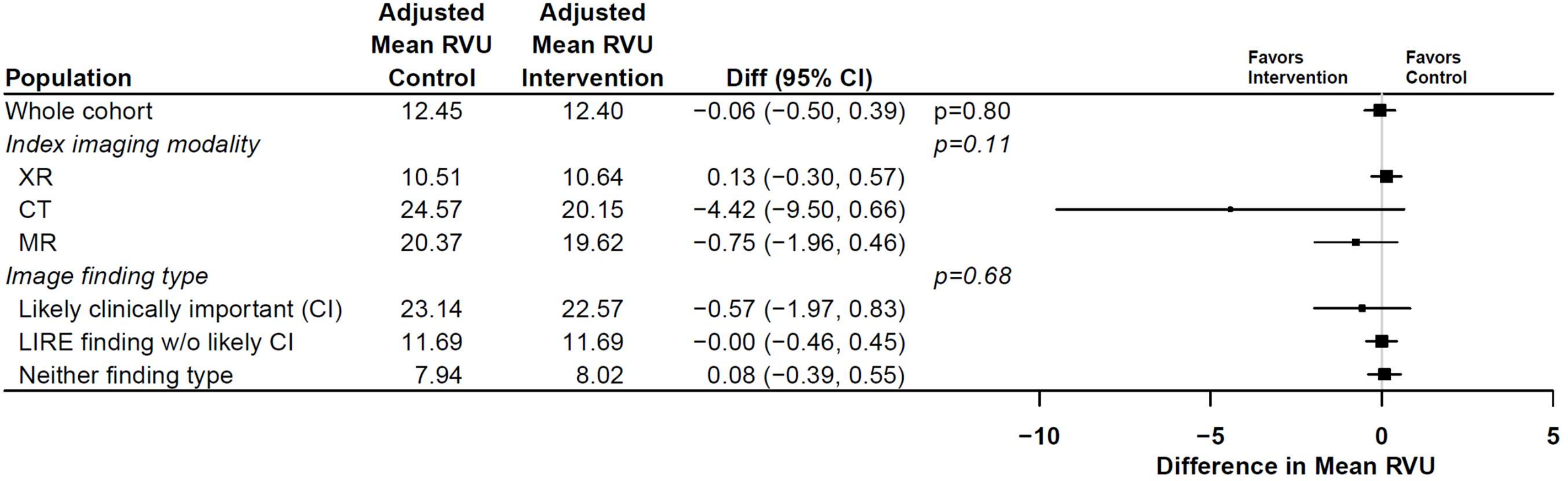

All models adjust for health system, clinic size, age range (18-39, 40-60, 61+), gender, imaging modality, Charlson Comorbidity Index category (0, 1, 2, 3+), and health system specific time trends and include hierarchical random effects for clinic (intercept and treatment) and provider (intercept only). P values for subgroup models (index imaging type and image finding type) are for Wald tests for effect modification.

Abbreviations: RVU – relative value unit; 95% CI – 95% confidence interval; XR – x-ray; CT – computed tomography; MR – magnetic resonance

© 2020 Jarvik JG et al. *JAMA Network Open.*

eAppendix 6. Opioid Prescription Within 90 Days

eFigure 7. Opioid prescription within 90 days

Population	Adjusted Opioid Rate Control	Adjusted Opioid Rate Intervention	Odds Ratio (95% CI)	
Whole cohort	29.8%	28.9%	0.95 (0.90, 0.99)	p=0.02
Prior opioid Rx				
No	18.1%	17.5%	0.96 (0.89, 1.03)	
Yes	63.5%	61.8%	0.93 (0.85, 1.01)	
Alternative Time Definition				
Spline 1 knot	29.9%	28.9%	0.95 (0.90, 0.99)	p=0.02
Spine 2 knots	29.8%	28.9%	0.95 (0.90, 0.99)	p=0.02
Alternative Provider Definition				
Rx from any provider	34.9%	34.2%	0.97 (0.92,1.01)	p=0.12
Rx from non−LIRE provider	9.9%	10.0%	1.00 (0.96, 1.05)	p=0.84

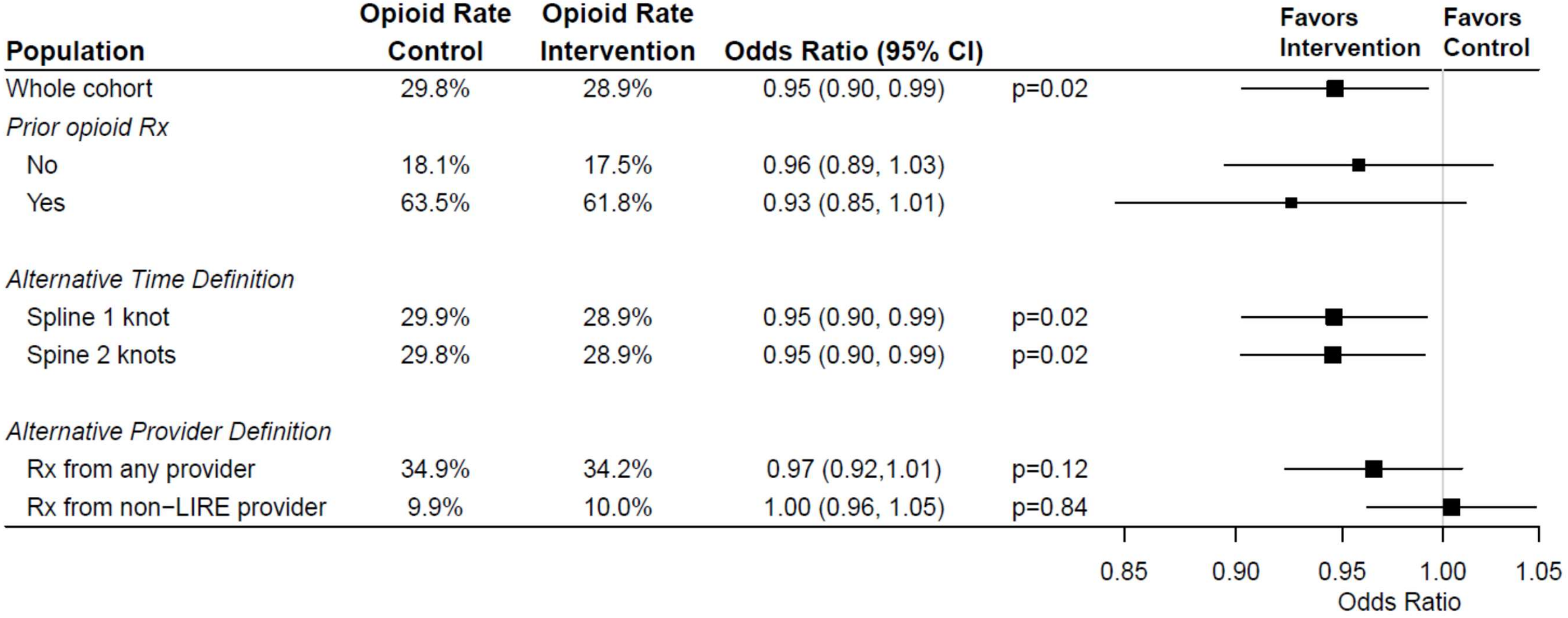

All models adjust for health system, clinic size, age range (18-39, 40-60, 61+), gender, imaging modality, Charlson Comorbidity Index category (0, 1, 2, 3+), prior opioid use, and health system specific time trends and include hierarchical random effects for clinic (intercept and treatment) and provider (intercept only). Prior opioid use is defined as having one or more prescriptions in the 120 days prior to index imaging. A *LIRE provider* is any provider who ordered an index lumbar spine image for one or more participants in the LIRE trial. It need not be the same provider who ordered the patient's index image. A *non-LIRE provider* is any other provider. *Any provider* includes both LIRE and non-LIRE providers.

Abbreviations: 95% CI – 95% confidence interval; Rx – prescription

© 2020 Jarvik JG et al. *JAMA Network Open.*

eAppendix 7. Patient Characteristics and Outcomes by Index Modality

eTable 8. Baseline characteristics by index imaging modality

Characteristic	XR (N = 192,435)	CT (N = 943)	MR (N = 45,508)
Site, No. (%)			
A	12,129 (6)	70 (7)	2,139 (5)
B	158,090 (82)	544 (58)	38,370 (84)
C	13,266 (7)	158 (17)	2,148 (5)
D	8,950 (5)	171 (18)	2,851 (6)
Age in years, No. (%)			
18-39	35,636 (19)	81 (9)	7,625 (17)
40-60	70,640 (37)	229 (24)	19,158 (42)
>60	86,159 (45)	633 (67)	18,725 (41)
Gender[a], No. (%)			
Female	112,118 (58)	477 (51)	24,778 (54)
Male	80,306 (42)	466 (49)	20,727 (46)
Charlson Comorbidity Index, No. (%)			
0	123,715 (64)	393 (42)	28,971 (64)
1	33,445 (17)	183 (19)	8,240 (18)
2	18,461 (10)	144 (15)	4,606 (10)
3+	16,814 (9)	223 (24)	3,691 (8)
Finding status, No. (%)			
None	53,969 (28)	67 (7)	1,510 (3)
LIRE finding without likely clinically important finding	137,910 (72)	215 (23)	11,067 (24)
Likely clinically important finding	556 (0)	661 (70)	32,931 (72)
One or more opioid prescriptions prior to index, No. (%)	43,737 (23)	467 (50)	17,327 (38)
Primary insurance at index, No. (%)			
Medicare	74,128 (39)	609 (65)	16,104 (35)
Medicaid/state-subsidized	9,819 (5)	46 (5)	2,191 (5)
Commercial	104,949 (55)	275 (29)	26,519 (58)
VA	190 (0)	--	58 (0)
Self-pay	1,108 (1)	7 (1)	186 (0)
Unknown or Not Reported	2,241 (1)	6 (1)	450 (1)
Socioeconomic index[b], mean (SD)	56 (6)	56 (6)	58 (7)
Provider type, No. (%)			
MD	171,362 (89)	835 (89)	41,327 (91)
DO	14,231 (7)	67 (7)	2,990 (7)
Extender (NP, PA, etc.)	6,842 (4)	41 (4)	1,191 (3)
Provider specialty, No. (%)			
Family medicine	96,867 (50)	430 (46)	19,775 (43)
Internal medicine	94,047 (49)	509 (54)	25,286 (56)
Other	1,521 (1)	4 (0)	447 (1)
Provider gender female, No. (%)	102,809 (53)	430 (46)	22,281 (49)
Provider age in years[c], mean (SD)	49 (9)	56 (6)	58 (7)

a. Does not include 14 patients with other or unknown gender.

b. Does not include 6,810 (3%) patients with unknown socioeconomic index.

c. Does not include 424 patients for whom provider age is unknown.

Abbreviations: No – number; XR – x-ray; CT – computed tomography; MR – magnetic resonance; VA – Veteran's Administration; SD – standard deviation; MD – medical doctor; DO – doctor of osteopathy; NP – nurse practitioner; PA – physician's assistant

© 2020 Jarvik JG et al. *JAMA Network Open.*

eTable 9. Outcomes by index imaging modality

Outcome	XR (N = 192,435)	CT (N = 943)	MR (N = 45,508)
RVU at 1 year, mean (SD)			
Manual (e.g. PT)	2.0 (5.6)	3.1 (7.4)	2.5 (6.3)
Evaluation & management	3.6 (7.2)	7.8 (9.4)	6.4 (8.5)
Injections	0.9 (3.6)	3.0 (7.3)	2.9 (6.2)
Imaging	2.2 (5.0)	3.3 (6.6)	2.3 (5.6)
Surgery	1.8 (17.8)	5.3 (25.8)	5.8 (28.9)
Total	10.6 (26.3)	22.5 (39.3)	20.0 (39.0)
Opioid prescription within 90 days, No. (%)			
LIRE provider[a]	55,466 (29)	412 (44)	14,317 (31)
Any provider[b]	64,478 (34)	484 (51)	17,613 (39)

a. LIRE providers are providers who ordered an index image for one or more LIRE Trial patients.

b. Any provider includes both LIRE and non-LIRE providers.

Abbreviations: No – number; XR – x-ray; CT – computed tomography; MR – magnetic resonance; SD – standard deviation; PT– physical therapy

© 2020 Jarvik JG et al. *JAMA Network Open.*

eReferences

1. Loudon K, Treweek S, Sullivan F, Donnan P, Thorpe KE, Zwarenstein M. T**he PRECIS-2 tool: Designing trials that are fit for purpose**. *BMJ*. 2015;350:h2147. doi:10.1136/bmj.h2147

2. Johnson KE, Neta G, Dember LM, Coronado GD, Suls J, Chambers DA, Rundell S, Smith DH, Liu B, Taplin S *et al*: **Use of PRECIS ratings in the National Institutes of Health (NIH) Health Care Systems Research Collaboratory**. *Trials* 2016, **17**:32.

© 2020 Jarvik JG et al. *JAMA Network Open.*
