# Supplementary material for: The Effect of Including Benchmark Prevalence Data of Common Imaging Findings in Spine Image Reports on Health Care Utilization Among Adults Undergoing Spine Imaging: A Stepped-Wedge Randomized Clinical Trial
Source: JAMA Netw Open. 2020 Sep 4;3(9):e2015713. doi: 10.1001/jamanetworkopen.2020.15713 (PMC7489827; doi:10.1001/jamanetworkopen.2020.15713)
Supplement: Supplement 2. — Trial Protocol and Statistical Analysis Plan [file jamanetwopen-e2015713-s002.pdf]

Lumbar Imaging Reporting with Epidemiology (LIRE)

Study Protocol

Version 3.0

Table of Contents

1.0 Project Summary ……………………………………………………………………………4

1.1 Project Organization

Schema 1: Overall Organization Chart

Schema 2: Site Organization Chart

Personnel Directory

1.2 Interaction with Collaboratory Coordinating Center

2.0 Background and Rationale……………………………………………………………………13

Figure 1: MRI of spine

Figure 2: Epidemiologic Statement Included In Lumbar Spine MR Imaging Reports

Table 1: Outcomes Of Patients Whose Imaging Did And Did Not Include A Statement Containing Epidemiological Benchmarks

3.0 Specific Aims…………………………………………………………………………………16

4.0 Study Details…………………………………………………………………………………17

4.1 Eligibility Criteria

4.2 Consent procedure

4.3 Inclusion and Exclusion Criteria

5.0 Research Design and Methods……………………………………………………………18

5.1 Clinic/Practitioner/Patient Identification

5.2 Randomization

Figure 3: Proposed Randomization Schedule

5.3 Clinic/Patient Enrollment

Table 2: Lumbar Examinations to be Included in Pragmatic Trial

5.4 Data Collection

5.5 Aims for UH2 Phase

Table 3: Age-specific Rates of Lumbar Spine Imaging Findings

Figure 4: Comparison of Parallel, Crossover and Stepped Wedge Designs

5.6 Working Groups

Table 4: Milestones for UH2 Planning Year Needed to Transition to UH3 Implementation

5.7 Aims for UH3 Phase

Table 5: Sample of RVUs and CMS-based payment amounts for lumbar imaging

Table 6: Milestones for UH3 (Implementation Phase)- Years 2-5

Table 7: Timeline for the UH3 Phase (Years 2-5)

6.0 Statistical Considerations……31

7.0 Human Subjects ……31
7.1 Human Subjects Involvement and Characteristics
7.2 Research Data
7.3 Potential risks
7.4 Adequacy of Protection Against Risks
7.5 Potential Benefits of the Proposed Research to the Subjects
7.6 Importance o the Knowledge to be Gained

8.0 Data and Safety Monitoring……33

9.0 References……34

10.0 Appendices

Appendix A: Article Extraction form (Working Group 1)
Appendix B: Literature Search and Articles used in Intervention text (Working Group 1)
Appendix C: Pilot Implementation Site Checklist (Working Group 2)
Appendix D: Literature Search for RVU-based Assessment (Working Group 3)

1. Project Summary

Low back pain, an Institute of Medicine priority condition for comparative effectiveness research, is of major public health importance. It is one of the most common reasons for physician visits and an important cause of functional limitation and disability. Imaging is frequently performed as part of the diagnostic evaluation and is an important contributor to the cost of back pain care, which totaled more than $86 billion in 2005. It is well known that, even without back pain, magnetic resonance (MR) imaging of the lumbar spine frequently reveals findings such as disc desiccation or bulging. Patients and their providers may attribute greater importance to these findings, which are often age-related, than they should, because they do not have an appropriate frame of reference in which to interpret the findings. These “incidental” findings may initiate a cascade of events leading possibly even to surgery, without improving patient outcomes.

The overall goal of the Lumbar Image Reporting with Epidemiology (LIRE) trial, is to perform a large, pragmatic, randomized controlled trial to determine the effectiveness of a simple, inexpensive and easy to deploy intervention – of inserting epidemiological benchmarks into lumbar spine imaging reports – at reducing subsequent tests and treatments. The long-term public health significance is that our intervention has the potential to substantially reduce unnecessary and expensive care not only for back pain, but also for a wide range of other conditions, since it could easily be applied to other diagnostic tests (e.g. other imaging tests, laboratory tests, genetic testing). If our study is positive, adding epidemiologic benchmarks to diagnostic test reporting could become the dominant paradigm for communicating all diagnostic information.

We propose an efficient, novel, cluster randomized design referred to as a “stepped wedge” design, permitting longitudinal comparisons while controlling for temporal trends. We plan to passively collect primary outcome measures of healthcare utilization both pre- and post-intervention, using robust electronic medical records at the participating sites. We hypothesize that for patients of primary care providers, inserting epidemiological benchmarks in lumbar spine imaging reports will reduce subsequent diagnostic and therapeutic interventions, including MR and CT, opioid prescriptions, spinal injections and surgery. The rationale is that the epidemiologic data may provide a context for both physicians and patients to better interpret imaging findings.

The University of Washington will serve as the over data coordination center (DCC) for the project that will take place at four performance sites: Group Health Cooperative, Kaiser Permanente of Northern California, Henry Ford Health Systems, and Mayo Clinic Health Systems. The role of the DCC is to coordinate study efforts, thus overseeing the technical implementation of the intervention across the sites. The DCC will oversee the transfer and storage of study data, provide biostatistical and analysis expertise, as well as lead manuscript writing efforts. Each performance site is tasked with the implementation of the randomized intervention at the primary care clinics within their system, as well as the technical abstraction (and transfer) of electronic medical record (EMR) and administrative data from their system to the DCC. See Schemas 1 and 2 below for organizational overview.

1.1 Project Organization:

Schema 1: Overall Organization Chart

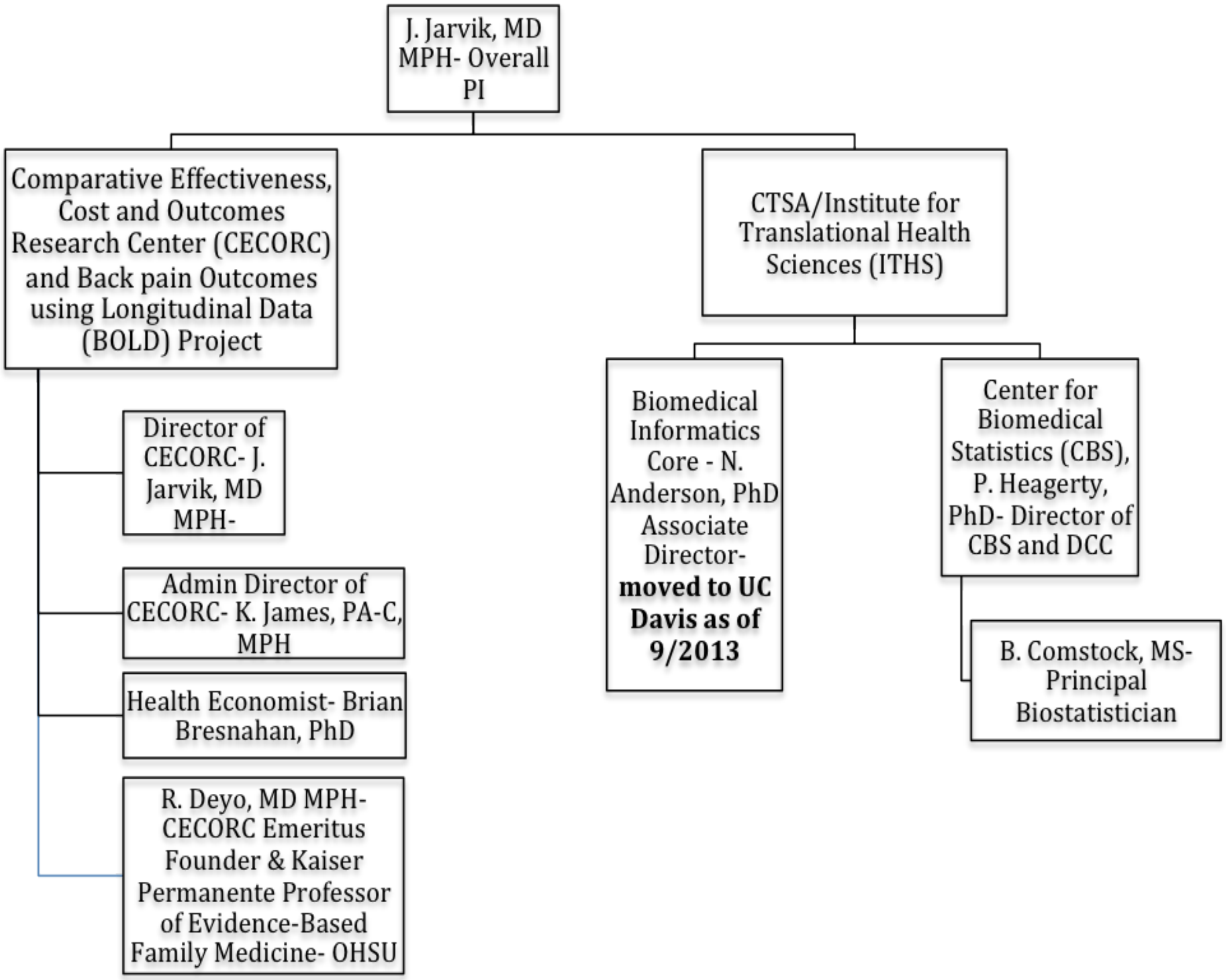

Schema 2: Site Organization Chart

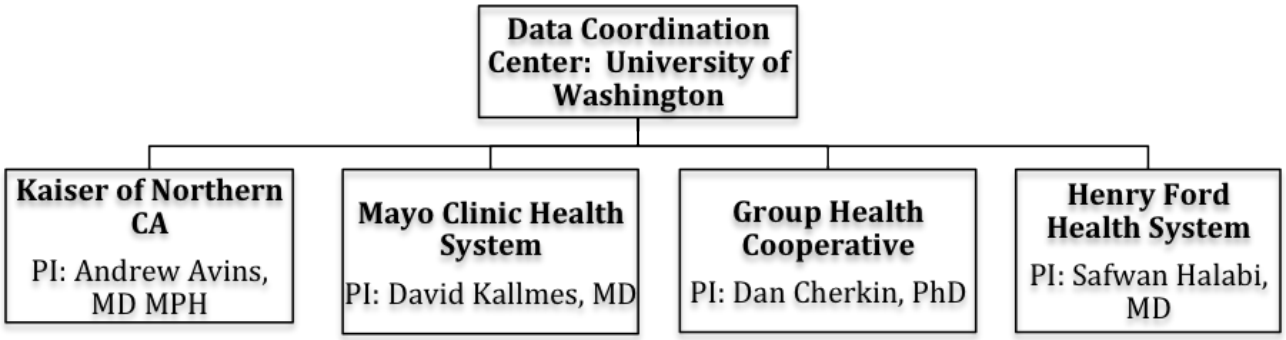

Personnel Directory

University of Washington
Data Coordination Center

Jeffrey G. Jarvik MD, MPH
Principal Investigator
Professor, Radiology and Neurological Surgery
Adjunct Professor, Health Services
Director, Comparative Effectiveness, Cost and Outcomes Research Center (CECORC)
University of Washington
Box 359455
4333 Brooklyn Ave NE
Seattle, WA 98195
Tel: (206) 616-2941
Fax: (206) 616-3135
Email: jarvikj@uw.edu

Patrick Heagerty PhD
Co-Investigator
Professor, Biostatistics
Director, Center for Biomedical Statistics
University of Washington
Box 357232
Seattle, WA 98104
Tel: (206) 616-2720
Fax: (206) 543-3286
Email: heagerty@uw.edu

Judith Turner PhD
Co-Investigator
Professor, Psychiatry and Behavioral Sciences, Rehabilitation Medicine
Adjunct Professor, Anesthesiology and Pain Medicine
University of Washington
Box 356560
Seattle, WA 98195
Tel: (206) 543-3997
Fax: (206) 685-1139
Email: jturner@uw.edu

Brian Bresnahan PhD
Health Economist
Research Assistant Professor, Radiology
University of Washington
Box 359736

325 Ninth Ave.
Seattle, WA 98104
Tel: (206) 744-1805
Fax: (206) 744-9962
Email: bres@uw.edu

Bryan Comstock, MS
Biostatistician
Operations Director, Center for Biomedical Statistics
University of Washington
Box 359461
Seattle, WA 98195
Tel: (206) 543-1882
Fax: (206) 543-5881
Email: bac4@uw.edu

Kathryn James PA-C, MPH
Overall Project Director
Administrative & Operations Director, Comparative Effectiveness, Cost & Outcomes Research Center (CECORC)
Box 359455
4333 Brooklyn Ave NE
Seattle, WA 98195
Tel: (206) 221-7444
Fax: (206) 616-3135
Email: jarvikj@uw.edu

UW Grant Contact: My-Hanh Tong - mhtt@uw.edu

Oregon Health Sciences University
Subcontracted Site for Consultant

Richard A. Deyo MD, MPH
Co-Investigator
Kaiser Permanente Professor of Evidence-Based Family Medicine
Director, KL2 Multidisciplinary Clinical Research Career Development Program
Director, OCTRI Community and Practice-based Research Program
Departments of Family Medicine and Internal Medicine
Oregon Health and Science University
3181 SW Sam Jackson Park Rd.
Portland, Oregon 97239-3098
Tel: (503) 494-1694
Fax: (503) 494-2746
Email: deyor@ohsu.edu

OHSU Grant Contact: Connie Yu- yuco@ohsu.edu

University of California- Davis
Subcontracted Site for Consultant

Nicolas Anderson, PhD
Co-Investigator
Nick Anderson, Ph.D.
Robert D. Cardiff Professor of Informatics
Director of Informatics Research
Department of Pathology and Laboratory Medicine
University of California, Davis
Tel: (916) 703 6976

UC Davis Grant Contract: Kate Marie- kate.marie@ucdmc.ucdavis.edu

Group Health Cooperative
Performance Site

Dan Cherkin, PhD
Site Principal Investigator
Group Health Research Institute
1730 Minor Ave, Ste 1600
Seattle, WA 98101
Email: cherkin.d@ghc.org

Heidi Berthoud MPH
Project Manager
Group Health Research Institute
1730 Minor Ave, Ste 1600
Seattle, WA 98101
Email: berthoud.h@ghc.org

GHC Grant Contact: David Hawkes- hawkes.d@ghc.org

Henry Ford Health System
Performance Site

Safwan Halabi MD
Site Principal Investigator
Associate Professor, Radiology
Director, Imaging Informatics
2799 West Grand Boulevard
Detroit, MI 48202
Email: safwanh@rad.hfhs.edu

David Nerenz PhD
Site Co-Investigator
Director, Outcomes Research
Neuroscience Institute
Department of Neurosurgery
Henry Ford Hospital
2799 West Grand Boulevard, K-11, W-1136
Detroit, MI 48202-2689
Tel: (313) 916-5454
Fax: (313) 874-7137
Email: dnerenz1@hfhs.org

Brooke Wessman
Project Manager
Tel: (313) 916-0829
Email: brookew@rad.hfhs.org

HF Grant Contact: Kim Sadlocha -ksadloc1@hfhs.org

Kaiser Permanente of Northern California
Performance Site

Andrew Avins MD, MPH
Site Principal Investigator
Clinical Professor, Medicine
Adjunct Professor, Epidemiology and Biostatistics
Kaiser Permanente Division of Research
2000 Broadway
Oakland, CA 94612- 2304
Office: (510) 891-3557
Fax: (510) 891-3606
Cell: (415) 302-5986
Email: andrew.avins@ucsf.edu

Luisa M. Hamilton
Project Manager, KPNC Division of Research
2000 Broadway
Oakland, CA 94612 -2304
Tel: (510) 891-3712
Fax: (510) 891-3802
Email: luisa.M.Hamilton@kp.org

KPNC Grant Contact: Anna Delaney-Heath- delaney@kp.org

Mayo Clinic Health System
Performance Site

David F. Kallmes MD
Site Principal Investigator
Professor, Radiology
Adjunct Professor, Neurological Surgery
200 First St. SW
Rochester, MN 55905
Tel: 507-266-3350
Email: kallmes.david@mayo.edu
Administrative Contact:
Kimberly Collins: Collins.kimberly@mayo.edu

Patrick Leutmer MD
Site Co-Investigator
Assistant Professor, Radiology
200 First St. SW
Rochester, MN 55905
Tel: 507-284-2097
Email: leutmer.patrick@mayo.edu

Jyotishman Pathak PhD
Site IT Consultant
Associate Professor, Biomedical Statistics and Informatics
200 First St. SW
Rochester, MN 55905
Tel: 507-284-5541
Fax: 507-284-0460
Email: pathak.jyotishman@mayo.edu
Administrative Contact:
Stacy Tapp: tapp.stacy2@mayo.edu

Beth Connelly
Project Manager,
Associate Clinical Research Coordinator, Department of Radiology
Tel: 507-538-3928
Pager: 507-293-4510
Email: connelly.beth@mayo.edu

Kristina Schmidtknecht
Site IRB Contact
Protocol Development Coordinator, Department of Radiology
Tel: 507 266-2082
Fax: 507-284-8249
Pager: 507 538-2477
Email: schmidtknecht.kristina@mayo.edu

Mayo Grant Contact: Tracey Anderson- anderson.tracey@mayo.edu

Sponsor Information:
National Institutes of Health (NIH)
Funding Award: 1UH2AT007766-01

Health Care Systems Research Collaboratory:
UH2/UH3 mechanism with UH2 as a planning year and UH3 a separate award for Yr 2-5 awarded after competitive review based on progress against UH2 milestones.
NIH press release regarding award: http://nccam.nih.gov/news/2012/092512
Collaboratory Website: www.nihcollaboratory.org

Timeline:
Budget Period: 09/30/2012 - 12/31/2013
Project Period: 01/01/2014 - 12/31/2017

Participating Institutions:

Data Coordinating Center (DCC) and Prime Awardee:
University of Washington- Seattle, WA
Principal Investigator: Jeffrey G. Jarvik, MD, MPH

Performance Sites:
Group Health Cooperative (GHC) and Group Health Research Institute: Site PI: Dan Cherkin, PhD
Henry Ford Health System (HFHS) Site PI: Safwan Halabi, MD
Kaiser Permanente of Northern California (KPNC) Site PI: Andy Avins, MD, MPH
Mayo Clinic Health System (MCHS) Site PI: David Kallmes, MD

Subcontracted Sites:
Oregon Health Sciences University (OHSU): Rick Deyo, MD, MPH
University of California- Davis (UCD): Nicholas Anderson, PhD (subcontract in UH2 phase only, contractor in UH3)

1.2 LIRE Interaction with Collaboratory Coordinating Center at Duke Clinical Research Institute:

The Collaboratory Coordinating Center has several “Cores” aimed at organizing topic-specific working groups across the seven demonstration projects. LIRE is contributing to the Collaboratory by assigning key team members to these Cores in the following ways:

- Electronic health records- Anderson, Jarvik, Comstock, and James
- Provider Health Systems Interactions- Jarvik and James
- Regulatory/Ethics- James
- Biostatistics/Study design- Heagerty and Comstock
- Stakeholder engagement- Jarvik and James
- Pheontype and Data Standards- Anderson

2. Background and Rationale

Summary of rationale: A common problem with many diagnostic tests is the discovery of incidental findings unrelated to patient symptoms or complaints. Such findings can lead to wasteful subsequent testing and intervention, sometimes with avoidable complications. Our overall goal is to test a strategy for mitigating these "cascade effects" of incidental findings. We focus on the example of lumbar spine imaging, where incidental findings are extremely common. We propose a pragmatic randomized trial of the strategy of inserting epidemiological evidence into routine spine imaging reports.

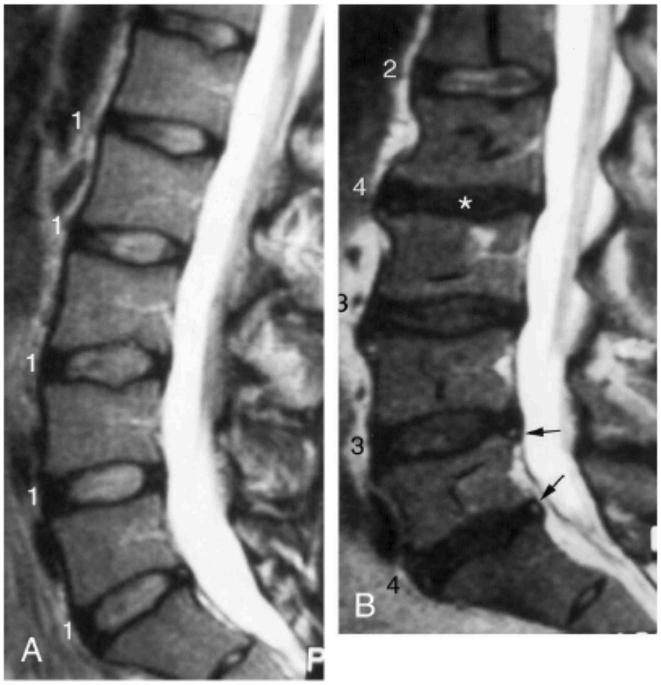

Figure 1. **A**, Normal disc hydration and height. Sagittal T2-weighted image demonstrates normal disc signal and height (rated as 1 [normal] on a 1–4 scale). **B**, Sagittal T2-weighted image demonstrates mild (2), moderate (3), and severe (4) desiccation. Also note height loss at L2–L3 (*) and anular tears seen as linear high signals at L4–L5 and L5–S1 (arrows).

If the study is positive, the method is likely to be generalizable to many other conditions and to other kinds of testing (eg, laboratory tests). So while back pain, especially the back pain that primary care providers see and treat, is incredibly important, our project can also be viewed as a "proof of concept" study that could open the doors to many similar interventions. Moreover, the potential cost/effectiveness of this intervention, if successful, is enormous. The cost of the intervention itself is minimal, yet substantial clinical and financial benefits could result. Few medical interventions can make that claim.

Back pain is one of the most important causes of functional limitation and disability worldwide and is an Institute of Medicine priority condition.(1,2) It is one of the most common reasons for physician visits.(3) The American College of Physicians (ACP) instituted a program in 2011 called High-Value, Cost-Conscious Health Care (HVCCHC).(4) The purpose of the program is "… to help physicians and patients understand the benefits, harms, and costs of an intervention and whether it provides good value, and to slow the unsustainable rate of health care costs while preserving high-value, high-quality care." The importance of back pain is highlighted by the first recommendations of the program being focused on the appropriate use of spine imaging. (5) In April 2012, the ACP in combination with the ABIM Foundation released their 5 top "Things that Patients and Physicians Should Question." Number two on the list was "Don't obtain imaging studies in patients with non-specific low back pain" (6).

Luo and colleagues estimate that the 1998 direct costs of low back pain in the U.S. were over $26 billion. More recently, Martin et al, estimated that the 2005 direct costs were over $86 billion. (7) Diagnostic imaging is a critical step in the work-up of back pain. It can quickly lead to a precise and actionable diagnosis, such as severe central spinal stenosis with cauda equina compression that may require rapid surgical consultation. But imaging examinations of the lumbar spine frequently reveal numerous findings, including disk desiccation, height loss, or bulging, with questionable relevance to patient symptoms.

Figure 1, from our study, Longitudinal Assessment of Imaging and Disability of the Back (LAIDBack) in 2001 demonstrates a lumbar spine MR without degenerative changes (Figure 1A) and a subject with extensive degenerative changes (Figure 1B). Neither of these subjects had low back pain. (8) These findings are common in asymptomatic adults, with prevalences in this group as high as 90%. (8-10) Moreover, multiple studies have failed to demonstrate clinical benefit with the use of early MR imaging for low back pain (LBP) compared with radiographs alone or no imaging at all; furthermore, the imaging results may negatively affect patients' sense of well-being (7,13,14). But diagnostic imaging of the lumbar spine can also lead to a cascade of subsequent tests and treatments that may have little beneficial impact on a patient's outcome and may even be deleterious. (11, 12) Because incidental findings are nearly ubiquitous with spine imaging, it is important to have a good understanding of the prevalence of various findings in asymptomatic patients.

While spine imaging may be one of the most common examples of incidental findings on diagnostic testing resulting in a cascade of subsequent tests and treatments, this situation is by no means limited to spinal diagnosis. Lung cancer screening with CT was recently shown to be beneficial in a high-risk population, but one of the concerns with such screening are the frequent benign nodules that are discovered. (13) Adrenal nodules seen on body CTs (14), thyroid nodules seen on neck and chest CTs (14), sinus mucosal thickening seen on head MR (15) and CTs (16) could all lead to subsequent diagnostic and therapeutic interventions if their prevalence in patients without disease was not well-understood.

While spine specialists are well aware of these prevalence data, non-specialists such as family practitioners and general internists may not know that a finding such as an annular fissure is seen in about one-third of asymptomatic patients and if present, is likely not related to a patient's pain.

Several years ago our group at the University of Washington implemented into our clinical practice, the recommendation of Roland and van Tulder (17) to include epidemiologic information in the radiology report to help physicians interpret findings frequently seen on lumbar spine imaging (Figure 2). By providing a context for these common findings, we hoped to mitigate concern and dampen any subsequent cascade of inappropriate testing and treatment.

FIGURE 2: EPIDEMIOLOGIC STATEMENT INCLUDED IN LUMBAR SPINE MR IMAGING REPORTS

Comment: The following findings are so common in people without low back pain that while we report their presence, they must be interpreted with caution and in the context of the clinical situation. (Reference –Jarvik et al, Spine 2001)

Findings: (prevalence in patients without low back pain), Disk degeneration (decreased T2 signal, height loss, bulge) (91%), Disk T2—signal loss (83%), Disk height loss (56%), Disk bulge (64%), Disk protrusion (32%), Annular tear (38%)

Multiple randomized controlled trials have shown that the early use of imaging for LBP is not associated with improved outcomes and may be harmful to the patient (11, 18-23). The American College of Physicians recently re-issued guidelines for imaging patients with LBP emphasizing not only the inefficiencies of early imaging but also the potential harms (24). Furthermore, as rates of MR imaging of the lumbar spine have increased, so too have treatments; including narcotics prescriptions, lumbosacral injections, and spinal surgery, often without benefit (25-32). Not only do these treatments result in increased expenditures (7, 32, 33), but, more importantly, they pose serious risks to the health of the patient. Narcotics are associated with multiple side effects, including respiratory depression, cognitive

impairment, constipation, and even death, as well as the development of tolerance and dependency (34, 35). Complications from spinal surgeries, especially more invasive fusions, include wound complications, major medical complications, and death (32).

In 2012 our group published a pilot study demonstrating insertion of such epidemiological evidence was associated with reduced narcotic prescriptions and a non-significant reductions in subsequent MR, CT and physical therapy as well. Taken together, these findings suggest primary care providers were more reserved in their management of patients whose MR report included the epidemiological evidence statement. (Table 1: from McCoullough et al, 2012) (36) Additionally, if patients learned about the statement, as some undoubtedly did since patients at the study site have direct access to their medical records, knowledge that their spine findings are common in patients without back pain might alleviate anxiety, which is known to have an important influence on pain. (37)

Table 1: **Outcomes of Patients Whose Imaging Did and Did Not Include a Statement Containing Epidemiological Benchmarks** (from McCoullough et al, 2012) (36)

Outcomes of Statement and Nonstatement Groups

Outcome	Statement Group ($n = 71$)	Nonstatement Group ($n = 166$)	Odds Ratio*	P Value
Cross-sectional reimaging	1 (1)	12 (7)	0.22 (0.03, 1.67)	.14
Narcotics prescription	5 (7)	37 (22)	0.29 (0.11, 0.77)	.01
Physical therapy	17 (24)	60 (36)	0.55 (0.29, 1.03)	.06
Steroid injection	11 (15)	22 (13)	1.37 (0.61, 3.05)	.44
Surgical consultation	20 (28)	58 (35)	0.86 (0.45, 1.66)	.67
Surgery	4 (6)	11 (7)	1.09 (0.32, 3.72)	.89

Note.—Unless otherwise indicated, data are numbers of patients, with percentages in parentheses.

* Odds ratio represents comparison of statement and nonstatement groups, while controlling for severity of MR imaging findings. Data in parentheses are 95% confidence intervals.

The relatively new field of clinical genomics is on the verge of a virtual explosion of genetic tests that will be inexpensive and readily available. (38, 39) However, genetic testing faces the same challenges of communicating risk information that more traditional diagnostic testing has faced for decades. (39) Lessons learned from diagnostic imaging may be applied to genomic testing and vice-versa.

Because our intervention is simple, inexpensive and can be automated, it is easy to implement on a large scale, making it nearly ideal to study in the context of a large, pragmatic trial in multiple health systems. We decided to confine our participating sites to large health systems that have sophisticated electronic medical records allowing us to passively collect our outcomes through electronic queries.

Our method of random assignment is also relatively novel. We propose to use a stepped wedge cluster design, where the order in which clinics receive the intervention is determined at random and by the end of the random allocation, all clinics will have received the intervention. (40, 41)

Finally, given the rapid spread and adoption of IT clinical tools, like the EMR and templates for radiology readings, finding ways to capitalize on the technology itself to positively influence the process of care will make the mammoth nationwide clinical IT investment much more compelling. This project is truly

emblematic of the kinds of innovative thinking that needs to be applied to the clinical IT world to derive the maximum benefit of the tools meant to deliver better and more efficient care.

3. Specific Aims

This study is a pragmatic cluster randomized controlled trial, randomly assigning primary care clinics at four sites, to receive either standard lumbar spine imaging reports or reports containing epidemiological benchmarks for common imaging findings. Our primary outcome will be a metric of back-related intervention intensity, measured passively using the electronic medical record (EMR). The primary analysis will focus on clinic-level changes by using aggregate patient-level data.

Aim 1: To determine whether inserting a description of age-specific prevalence of imaging findings among asymptomatic subjects into lumbar spine imaging reports decreases back-related interventions imaging, injections, surgeries, etc.) over the subsequent year.

Aim 1a: To determine if inserting epidemiological evidence reduces Relative Value Units (RVUs) attributable to spine interventions (imaging, injections, specialist referrals, surgeries, etc.).

Hypothesis 1a: After primary care clinics are randomly assigned to receive the modified report, they will have a lower average overall RVU (technical and professional) per imaged-patient attributable to spine interventions than when clinics are not receiving the modified reports. Spine interventions reflect visits, tests, and procedures and are patient centered, having both direct and indirect impacts on patients.

Aim 1b: To determine if inserting epidemiological data decreases opioid prescriptions.

Hypothesis 1b: Time periods during which clinics are randomly assigned to receive the modified imaging reports will have a lower rate of subsequent opioid prescriptions than time periods during which clinics do not receive modified reports.

Aim 1c: To determine if inserting epidemiological evidence decreases subsequent cross-sectional imaging magnetic resonance (MR) and computed tomography (CT).

Hypothesis 1c: Time periods during which randomly assigned clinics receive modified imaging reports will have a lower rate of subsequent cross-sectional imaging than time periods for which clinics do not.

Aim 1d: To explore whether adding epidemiological evidence decreases overall costs of care for low back pain based on CMS reimbursement.

Hypothesis 1d: Clinics that are randomly assigned to receive the modified imaging reports will have lower back pain-related estimated payer costs than clinics whose patients do not receive modified reports. Costs are another outcome that are highly relevant to both patients and health systems.

Aim 2: To determine whether inserting age-specific prevalence of imaging findings in asymptomatic subjects has a differential effect on subsequent back-related interventions if inserted into lumbar spine MR and CT imaging reports compared with plain films.

Hypothesis 2: Inserting epidemiological information into plain film reports will result in a greater decrease in subsequent back-related interventions than similar information put into MR and CT reports. Given that plain films are generally obtained earlier in the course of back pain and more frequently than MR and CT, the potential impact of inserting epidemiological information into plain film reports is large.

Aim 3: To determine if specific imaging findings influence subsequent interventions.

Hypothesis 3: Inserting the statement will result in a greater decrease in subsequent interventions for patients without clinically important findings compared with patients who have clinically important imaging findings. Our work and others have shown that certain imaging findings are likely to be clinically more important than others (e.g. nerve root compression, moderate to severe central stenosis, disc extrusions). We expect that patients without these more important findings will have a greater reduction in subsequent interventions.

4. Study Details

4.1 Eligibility Criteria

Because this is a pragmatic trial, we have minimized eligibility restrictions, making the inclusion criteria as broad as possible. Clinics will be the primary unit of randomization and analysis, while the intervention will be applied at the individual patient level. Thus two sets of eligibility criteria are necessary: clinic and patient.

The criteria for *clinic eligibility* are that the health care providers are a distinct, readily identifiable group that has at least a subgroup of primary care providers who do not practice at another clinic that will also be part of the trial. This requirement of being based primarily at one site is to minimize cross-contamination (having the use of epidemiological benchmarks at one site influence another site not receiving the benchmarks).

The criteria for *patient eligibility* are that they have had an imaging study of the lumbar spine requested by a primary care provider. We will include all conventional lumbar spine imaging (plain films, CT, MR) ordered by primary caregivers.

4.2 Consent procedure

Because the intervention will be administered at the clinic level, consent of either individual patients or primary caregivers is neither feasible nor warranted. Moreover, the intervention is relatively benign (the insertion of additional epidemiological information into the radiology report) and poses minimal risk to caregivers and patients. The performance sites are enthusiastic about incorporating the epidemiological benchmarks into their reports and may well eventually adopt them regardless of the project, our study simply allows for systematic study of the effects of a well-controlled implementation of the insertion of the benchmark information. The randomization scheme defines when each clinic begins including the epidemiological information into the reports, with all sites eventually receiving the intervention of interest.

4.3 Inclusion and Exclusion Criteria

We will define a clinic as a primary care clinic if a majority of the practitioners at that clinic are providing

primary care. We will include general internal medicine and family practice physicians as primary caregivers as well as mid-level providers working with physicians such as nurse practitioners and physician assistants.

We will include all adult patients of eligible caregivers who have had a lumbar spine imaging study plain film, CT or MR) ordered by their primary care practitioner.

5. Research Design and Methods

5.1 Clinic/Practitioner/Patient Identification
The site PI will identify eligible clinics within their health system, working closely with their administrative and information technology (IT) staff to assure complete inclusion of primary care clinics. The site PIs will then categorize practitioners within each clinic by specialty, designating general internists, family practitioners and obstetrician/gynecologists as primary care practitioners. Mid-level providers (e.g. nurse practitioners and physician assistants) working as primary caregivers will also be classified as primary care practitioners. The health information system will be used to automatically identify when a practitioner from a particular clinic orders a lumbar spine imaging study.

5.2 Randomization
At each site we will identify the settings where primary care is delivered and designate an appropriate unit that will constitute a functional "clinic" for randomization and analysis. We will randomly assign all predetermined clinics at each site to receive the intervention at one of five fixed time-points, rolling interventions out every six months beginning at the start of the second quarter of Year 2. Using cutoffs determined in the UH2 project phase, we will sort clinics by number of primary care providers into tertiles (e.g. small, medium, large clinics). From each tertile we will randomly select clinics using urn-based randomization (without replacement) stratified by site and clinic size such that clinics of small, medium, and large size are equally represented in each randomization wave. For more details regarding the Analysis plan, please refer to the UH3 transition request proposal and accompanying Appendix 10, Analysis plan that reflects modifications made to the original plan we outlined for UH2.

Figure 3: Proposed Randomization Schedule

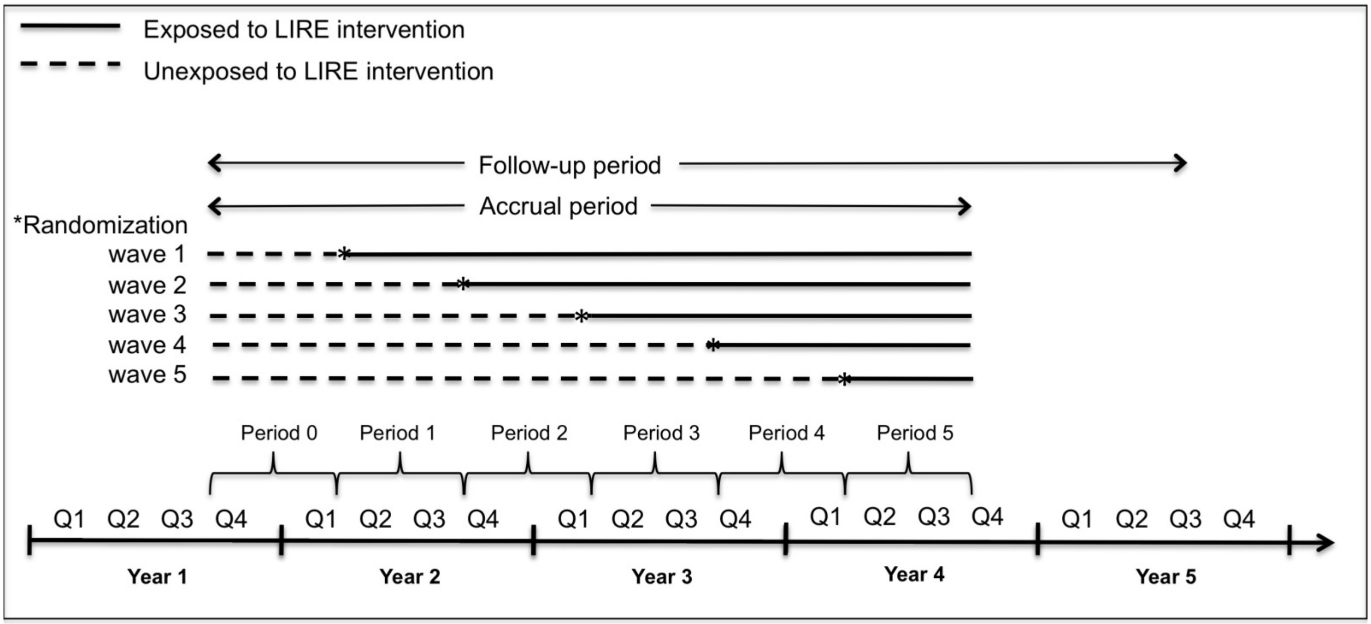

5.3 Clinic/Patient Enrollment

Using the site administrative data systems, we will identify all primary care providers (PCP) at a given clinic. When an identified PCP from a randomized clinic submits a request for a lumbar spine imaging study, the report will automatically be flagged. The PCP's name will be cross-referenced with the randomization assignment and those PCPs who work in clinics assigned to receive the intervention will have the epidemiological benchmark information automatically inserted into their imaging reports. Those PCPs who work in clinics not yet scheduled to receive the benchmark information will get the usual imaging report issued by their radiologists. Since the intervention will be applied at the PCP and clinic level, all patients receiving lumbar spine imaging studies at those clinics will be part of the trial. The lumbar spine imaging studies that we plan to include in the trial are plain films, magnetic resonance (MR) imaging examinations and computerized tomography (CT). Table 2 lists the proposed CPT codes that we will flag for inclusion. We are currently not planning on including nuclear medicine studies (e.g. bone scans, both planar and SPECT) both because they are infrequently ordered by primary care clinicians as well as because there is inadequate benchmarking information.

Table 2: Lumbar Examinations to be Included in Pragmatic Trial

CPT Code	Examination Description
72080	THORACOLUMBAR SPINE, 2 views
72100	LUMBAR SPINE 2 VIEWS
72110	LUMBAR SPINE 3-4 VIEWS
72114	LUMBAR SPINE 5 VIEWS
72131	CT L SPINE W/O CONTRAST
72132	CT L SPINE W/ CONTRAST
72133	CT L SPINE W/O & W/ CONTRAST
72148	MRI LUMBAR SPINE W/O CONTRAST
72149	MRI LUMBAR SPINE W/ CONTRAST
72158	MRI L SPINE W/ & W/O CONTRAST

5.4 Data collection

We will collect all baseline and follow-up data from the electronic information systems which, depending on the site, will include both the electronic medical record (EMR) as well as administrative data systems.

Baseline Data Collection: We will include all patients receiving lumbar spine imaging studies (plain films, MR and CT) in the last quarter of Year 1 and the first quarter of Year 2 as a part of a baseline accrual period to establish baseline parameters for the primary care physicians in participating clinics. Since the randomization will occur at the clinic level, the baseline data will reflect clinic level ordering patterns of diagnostic and therapeutic interventions.

Follow-up Data Collection: We will capture EMR data on patients for a minimum of one and up to two years after the index imaging test. All patients will have a minimum of one-year follow-up. Eighty percent of patients will have two-year follow-up due to the staggered implementation of the

intervention. The six-month length of each patient accrual period is sufficiently long to account for temporal or system-level trends in the measured outcomes over the course of the study.

5.5 Aims for UH2 Phase

Our goal is to use the planning UH2 phase of the grant to accomplish the following: First, we will refine the epidemiological benchmarks that we will insert into the radiology report. Second, we will develop and test our site-specific deployment method for the cluster randomization. Third, we will develop a metric that reflects the intensity of interventions for back pain-related care and develop CMS-based standardized cost estimates associated with resource use intensity that can be applied uniformly among health systems. We will validate this metric using data from the health care systems electronic medical record. Fourth, we will develop and validate our methods for extracting outcome data from the electronic medical record. Fifth, once we have defined the above, we will obtain Institutional Review Board approval for the implementation phase of the study. We will also use this time to assemble subcontracts for administrative review at each site.

<u>Aim 1:</u> Refine the information to be included in the radiology report so that it is specific for imaging modality and patient age.

In our original implementation, we only inserted the epidemiological benchmarks into reports of lumbar spine MRs whereas in the current project we propose to insert the information into reports of MR, CT and plain films. Moreover, we used epidemiological data from a single study published by our group (Table 3).(8) Other groups have published similar data for MR as well as other modalities. (9, 10, 42-59) In addition to updating the epidemiological benchmarks and expanding them to other modalities, we will also gather data regarding age-specific rates for various imaging findings. While eventually we would envision a decision support tool that could recognize specific patient attributes, such as age or the presence of a particular finding, and insert customized benchmark data for that individual, such a system is beyond the scope of this project. Instead we plan to insert benchmarks that are simply stratified by age ranges. We will perform a systematic review of the literature so that we are inserting the most recent and complete epidemiological evidence into the radiology report.

Table 3: Age-specific Rates of Lumbar Spine Imaging Findings (from Jarvik et al, 2001)

	Age Group						
Imaging Finding	<45 yr (n = 31)	45–55 yr (n = 53)	55–65 yr (n = 35)	>65 yr (n = 29)	*t* Statistic*	2-sided *P*	Adjusted OR† (95% CI)
Disc degeneration	24 (77)	49 (93)	32 (91)	29 (100)	−3.35	<0.01	1.13 (1.04, 1.23)
Desiccation (moderate or severe)	20 (65)	42 (79)	32 (91)	29 (100)	−4.14	<0.01	1.12 (1.05, 1.20)
Loss of disc height	13 (42)	27 (51)	23 (66)	20 (69)	−2.56	0.01	1.06 (1.02, 1.10)
Bulge	14 (45)	34 (64)	23 (66)	24 (83)	−3.55	<0.01	1.08 (1.03, 1.13)
Protrusion	9 (29)	18 (34)	11 (31)	10 (35)	−0.01	0.99	1.01 (0.97, 1.05)
Extrusion	0 (0)	6 (11)	2 (6)	1 (3)	0.34	0.73	1.01 (0.92, 1.10)
Nerve root compromise	0 (0)	2 (4)	1 (3)	2 (7)	−0.90	0.37	1.08 (0.96, 1.21)
Annular tear	12 (39)	19 (36)	13 (37)	12 (41)	0.14	0.89	0.99 (0.96, 1.03)
Endplate changes	1 (3)	9 (17)	15 (43)	14 (48)	−5.00	<0.01	1.13 (1.07, 1.19)
Stenosis (moderate or severe)	2 (7)	3 (6)	4 (11)	6 (21)	−2.07	0.04	1.09 (1.02, 1.16)
Facet joint degeneration (moderate or severe)	0 (0)	4 (8)	12 (34)	11 (38)	−4.39	<0.01	1.14 (1.07, 1.21)
Spondylolisthesis	2 (6)	5 (9)	9 (26)	10 (35)	−3.33	<0.01	1.09 (1.03, 1.15)

* Independent samples *t* test.
† Adjusted odds ratio from logistic regression analysis: $y = \alpha + \beta_1\text{Age} + \beta_2\text{Gender} + \beta_3\text{Race} + \beta_4\text{Smoking} + \beta_5\text{Height} + \beta_6\text{Weight} + \beta_7\text{BMI} + \beta_8\text{Past pain}$, where y = imaging finding and $\exp(\beta_1)$ = adjusted odds ratio.

<u>Aim 2:</u> Develop site-specific deployment methods for the stepped wedge, cluster randomization scheme.

Figure 4: Comparison of Parallel, Crossover and Stepped Wedge Designs (from Hussey et al., 2007)

M.A. Hussey, J.P. Hughes / Contemporary Clinical Trials 28 (2007) 182–191 183

Parallel

Cluster	Time 1
1	1
2	1
3	0
4	0

Crossover

Cluster	Time 1	Time 2
1	1	0
2	1	0
3	0	1
4	0	1

Stepped Wedge

Cluster	Time 1	2	3	4	5
1	0	1	1	1	1
2	0	0	1	1	1
3	0	0	0	1	1
4	0	0	0	0	1

Treatment schedules for parallel, crossover, and stepped wedge designs. "0" represents control or existing treatment; "1" represents an intervention.

The unit of randomization for this project will be at the level of the clinic. The stepped wedge design is a one-way cluster, crossover design that temporally spaces the intervention and assures that each participating clinic will eventually receive the intervention. Figure 4, from Hussey et al, (41) compares the stepped design with parallel and crossover designs:

Our experience with the BOLD project and other multicenter studies informs us that the procedures and hurdles at each site will be different so that our approach for implementing the cluster randomization must be customized at least to some extent. In the UH2 phase of the project, we will work closely with the informatics groups at each health system to develop the schedule of clinics to be randomized to receive the insertion of epidemiological data into the relevant reports. A key component of deploying the intervention is that it be automated and not require a radiologist to actively insert benchmark statements into the report. We also plan to create a process for notifying sites of intervention deployment, being careful to minimize opportunities for internal or external sources of contamination.

<u>Aim 3:</u> Develop and validate a composite measure of spine intervention intensity that combines into a single metric the overall intensity of resource utilization for back pain care.

Relative value units (RVUs) are a measure of work effort associated with a particular medical service. Although there are potential drawbacks to RVUs, including overvaluing certain services relative to others, it is a widely used metric and one potential method for measuring the intensity of services provided (or resources utilized) for back pain treatment. During the planning year, our group would need to specifically identify services that would comprise the back care intensity metric.

Once defined, we could then attempt to validate the metric using the World Health Organization's stepped care approach to pain treatment, which amounts to an escalating ladder of treatment intensiveness.

<u>Aim 4:</u> Develop and validate electronic data methods and tools to capture the outcomes of interest (subsequent diagnostic testing, opioid prescriptions, spinal injections, spine surgeries).

While all of the health care systems that we propose to involve in our project have sophisticated and comprehensive electronic medical records systems, we know from experience that accessing the relevant data and transmitting it to the project's data coordinating center will require careful planning and individualized approaches for each health system. We have already successfully implemented methods for collecting some of this information from 2 of our proposed sites (Kaiser Northern California and Henry Ford Health System, Detroit, MI) and would refine and deploy these methods at two new sites, Group Health Cooperative in Seattle, WA and the Mayo Clinic Health System in Minnesota and Wisconsin.

5.6 Working Groups

The planning UH2 phase of the grant will be used to accomplish the following: First, we will refine the epidemiological benchmarks that we will insert into the radiology report. Second, we will develop and test our site-specific deployment method for the cluster randomization. Third, we will develop a metric that reflects the intensity of interventions for back pain-related care and develop Medicare-based standardized cost estimates associated with resource use intensity that can be applied uniformly among health systems. We will validate this metric using data from BOLD and Medicare. Fourth, we will develop and validate our methods for extracting outcome data from the electronic medical record.

The scope of work for the UH2 planning year has been outlined and assigned to one of four working groups that align with the aims described above.

Working Group 1 will focus on refining the information to insert into the radiological report. They will lead a critical review of the literature focusing on the age-specific prevalence of common findings seen on plain films, CT and MR in people without low back pain. We will use meta-analytic methods to combine the prevalence estimates from multiple sources, weighting by study quality and relevance to the LIRE population. We will summarize the epidemiological information so that it can be inserted into plain film, MR and CT reports of lumbar spine imaging. Dr. Jarvik will lead this working group and work closely with Dr. Kallmes at the Mayo Clinic Health System.

Working Group 1 progress: Members of this working group developed and pilot tested the intervention text and took several steps in this process.

1. Comprehensive literature review for relevant articles regarding radiologic findings
2. Two independent reviews and data extraction of findings from relevant articles. See Appendices 1 and 2 for the Abstraction form used and list of final articles contributing data to the prevalence rates included in the intervention text.
3. Data cleaning and compilation
4. Data analysis and modeling to determine a) which findings had sufficient data to report on and b) age cut points
7. Consult with the Program for Readability In Science & Medicine (PRISM) scientific writing group at Group Health.
8. Key informant interviews with two patient advisors for feedback on format and readability
9. On-line survey with over 20 patient advisors for feedback and comment

Participating patient representatives were provided with a sample radiology report and four different versions of the intervention macro. They were asked the following questions:

- What is your age?
- Have you ever sought medical care for low back pain?
- Which format option did you prefer?
- Why did you prefer that format option over the others?
- Based on this information, would you say disc degeneration is common or rare?
- What is clear about the information presented?
- What is confusing about the information presented?
- How would you use the information presented in the option that you preferred?
- If you received a radiology report with the information in the option that you preferred, would you feel less or more concerned about having the imaging finding(s) that are common in people without back pain? For example, would you feel less or more concerned about having a degenerated disk?

10. On-line survey with seven primary care physicians for feedback and comment. Participating providers were provided with a sample radiology report and three different versions of the intervention macro. They were asked the following questions:

- What proportion of your patient visits are related to low back pain?
- Which format option do you prefer?
- Why did you prefer that format option over the others?
- Based on this information, would you say disc degeneration is common or rare?
- What could be improved about your preferred option?
- If you received a radiology report with the information in the option that you preferred, would you be less or more concerned about a given patient's imaging finding? For example, would you feel less or more concerned about a patient having a degenerated disk?
- How would the information presented in the option that you preferred inform your clinical decision making?

The final text is a product of the efforts described above and will serve as the final intervention. Different wording is offered based on 1) Age (<40, 40-60, and >60 years of age) and 2) Modality (plain film, CT, MR).

Working Group 2 will focus on methods to practically deploy the stepped wedge cluster randomization scheme. This will require working closely with the informatics group at each of the four sites to determine the optimal method for inserting the intervention text into their reports. This might occur at the level of the radiology reporting software (RRS), the radiology information system (RIS) or the hospital information system (HIS). Sites must demonstrate the ability to selectively insert the intervention text only into reports where the clinic is randomly allocated to receive the intervention. Dr. Heagerty and Mr. Comstock will lead this working group out the Center for Biomedical Statistics (CBS) that is part of the Institute for Translational Health Sciences (ITHS), the University of Washington's Clinical and Translational Science Award (CTSA). Dr. Heagerty is Professor and Associate Chair of Biostatistics and the Director of the CBS.

Working Group 2 progress: A site-readiness tool has been developed to guide efforts for pilot testing the insertion of the intervention macro across the clinics at a given site (see Appendix 3 for an example of this pilot testing document and site checklist). At the conclusion of this pilot, the number and size of

participating clinics at each site will be verified as well a final determination regarding technical feasibility of clinic randomization and intervention text insertion.

Working Group 3 will develop and validate the composite measure of spine intervention intensity, likely to be based on units and types of services used. We will validate this metric using data from a variety of sources including the Back pain Outcomes using Longitudinal Data (BOLD) registry. This will be an on-going effort through much of the planning year as we review the literature and obtain expert opinion to optimize the factors comprising the composite measure, and validate the measure. Drs. Deyo and Bresnahan will co-lead this effort. Working Group 3 will also develop cost estimates to apply to overall spine intervention intensity and to individual services.

Working Group 3 progress: A comprehensive review of the literature was conducted searching for articles pertaining to RVU-related assessment (see Appendix 4 for a complete list of articles). Mapping of CPT codes to relative value units has been completed using the BOLD Registry data. A manuscript of this work is currently being drafted. Mapping of codes determined to be "spine-related" has been completed utilizing previous work performed by a colleague currently at Dartmouth, Brook Martin, PhD, MPH.

Working Group 4 will develop and validate the methods to extract the necessary data to passively measure outcomes from each site's EMR. The group will perform test data pulls from each site of the key variables identified by Working Group 3. Anonymized data will be transmitted to the Data Coordinating Center at the UW, housed at the CBS. UW would only receive limited data sets without protected health information. Dr. Nick Anderson will lead this group, working closely with informatics experts at each of the sites. Dr. Anderson was Associate Director of the Bioinformatics Core at the UW ITHS at the start of the project and has since taken a position with University of California- Davis but will remain involved in the project.

Working Group 4 progress: An overall plan has been developed to leveraging PopMedNet to virtually connect the various implementation sites to UW who is to serve as the DCC for this project in anticipation of the data exchange which will take place in the UH3 phase. For EHR extraction, we anticipate utilizing the Virtual Data Warehouse at the three HMORN sites as much as possible, developing customized programming pieces that are necessary beyond that to further capture all the necessary data elements. Mayo clinic does not use the VDW so all data extraction programming will have to be customized at that site.

The working group activities as well as general project coordination will utilize the web-based tool, Basecamp (www.BaseCamp.com) to streamline efforts. All faculty and staff at the DCC, as well as Site PI's and study personnel at each site, will be given access to Basecamp. In addition, each working group has a dedicated "project" within Basecamp that will be used to facilitate discussions among group members, organize files, keep notes, and centralize study-related documents.

In order to coordinate efforts at each of the individual sites and assure the UH2 milestones are met in a timely fashion, site milestones have been outlined and site PIs and their respective research and technical teams are working towards these goals.

1."Radiology buy-in"

Assignee: Site PI/ Jarvik

Objective: Facilitate consensus among Radiologists that they are willing to have the intervention used

Deliverable #1: Letter of support from Radiology chair (template provided)

Due Date: July 31, 2013

2.“IRB approval”

Assignee: Site PI/ James

Objective: Coordinate IRB review such that waiver of consent and HIPAA for patients and waiver of consent for physicians is in in place

Deliverable #2: Final IRB approval documentation

Due Date: Final approval of application August 31, 2013

3. “Randomization Pilot”

Assignee: Site PI/ Comstock

Objective: Demonstrate ability to insert template into radiology report on schedule, randomized by clinic

Deadline: July 31, 2013

4. “EMR data extraction”

Assignee: Site PI/ Anderson

IT resource identified who Installs and authenticates the PopMedNet Client

Deadline: July 31, 2013

Identify a programming resource and validate a "starter set" VDW query that has been mapped against the LIRE data set

Deadline: August 31, 2013

Table 4: Milestones for UH2 Planning Year Needed to Transition to UH3 Implementation			
**UH2 Phase Milestone**	**Timeline**	**Suitability for Assessing UH2 Success**	**Importance to UH3 Success**
**Aim 1**:Complete critical literature review to refine the information to be included in the radiology report so that it is specific for imaging modality and patient age. **Jarvik, Bresnahan, Deyo, Halabi, Kallmes, Turner, Luetmer, Avins**	3 months	Highly suitable: Necessary to develop text describing age-specific epidemiologic benchmarks for plain films, CT and MR.	Critically important: Development and refinement of the intervention text is necessary for the timely implementation of the intervention.
**Aim 2**: Develop site-specific deployment methods for the stepped wedge, cluster randomization scheme **Comstock, Anderson, Avins, Cherkin, Halabi, Heagerty, James, Jarvik, Pathak, Murphy, Ciarelli,** ***Needed**: KP technical resource* ***Needed:** GHC technical resource*	3 months	Highly suitable: Will need detailed implementation protocols prior to implementation of intervention.	Critically important: Sites must have proven ability to selectively implement intervention text in the radiologic reports generated for providers at a given primary care clinic.
**Aim 3**: Develop and validate a composite measure of spine intervention intensity that combines into a single metric the overall intensity of resource utilization for back pain care and develop cost estimates associated with units of resource used and intensity of use. **Bresnahan, Deyo, James, Jarvik**	6 months	Suitable: Definition of composite measure to be used as primary outcome and validation using existing BOLD data.	Important: The achievement is important but not critical. While other metrics could be used successfully, including single parameters, a composite measure enables a more comprehensive estimate of overall care received for back pain.
**Aim 4:**Develop and validate electronic data methods and tools to capture the outcomes of interest (subsequent diagnostic testing, opioid prescriptions, spinal injections, specialist visits, spine surgeries, etc.) **Anderson, Comstock, James, Jarvik, Turner**	6 months	Highly suitable: Data dictionary and protocol to query the electronic medical record (EMR) necessary	Critically Important: Feasibility must be demonstrated in order to passively collect outcomes using the EMR.
**Additional Goals:** Data Safety Monitoring Plan (DSMP) formulation and designation of safety officer **Heagerty, Comstock, James, Jarvik**	6 months	Highly suitable: Drafting of DSMP, submission to IRBs,funding agency, and designation of Safety Officer needed prior to study initiation	Critically Important: A safety officer will need to review and approve the study DSMP.
**Additional Goals:** Draft study protocol **James, Comstock, Jarvik**	6 months	Highly suitable: Draft the study protocol so it incorporates the decisions made for Aims 1-4	Critically Important: The study protocol will need to be reviewed by the IRBs before the study receives final approval.

**Additional Goals:** IRB approval<br>**<u>James</u>, Avins, Cherkin, Halabi, Jarvik, Kallmes, Project Managers from each site (Hamilton, Connelly, Wessman, Hawkes)**	9-12 months	Highly suitable: Conditional IRB approval at multiple sites always challenging	Critically Important: IRB approval is required before study procedures can be initiated.
**Additional Goals:** Establish subcontracts with sites<br>**<u>James</u>, Avins, Cherkin, Halabi, Jarvik, Kallmes, Post-award personnel at each site**	9-12 months	Draft and submit subcontracts for the UH3 phase at all sites	Critically Important: Subcontracting with sites is a required process.

5.7 Aims for UH3 Phase

<u>Aim 1:</u> To determine whether inserting a description of age-specific prevalence of imaging findings among asymptomatic subjects into lumbar spine imaging reports decreases back-related interventions during the subsequent year.

Aim 1a: To determine if inserting epidemiological evidence reduces RVUs attributable to spine interventions.

Hypothesis 1a: After primary care clinics are randomly assigned to receive the modified report, they will have a lower average overall RVU (technical and professional) per imaged-patient attributable to spine interventions than clinics that are not receiving the modified reports.

We will calculate an overall spine-related RVU for each patient in the study by summing all RVUs attributable to spine-interventions within one and two years after the date of return of the index image report (the plain film, MR or CT of the lumbar spine imaging study whose report either does or does not contain the epidemiological benchmark data). For each patient accrual period in Figure 3, we will calculate a total spine-related RVU per primary care provider who orders at least one lumbar imaging exam in Table 2. We will aggregate spine-related RVUs across the study-eligible patient panel. The calculated primary care provider RVU will serve as the primary outcome measure of this study. As noted, we will also apply standardized CMS-based costs to RVU calculations.

We will use generalized linear mixed models with jackknifed standard to model the change in post-intervention RVU from pre-intervention RVU. We will use random effects for the clinic and for the effect of intervention defined by the indicator of exposure to the LIRE intervention. We will use a three-month pre-intervention washout period where patients with index visits in this window will be excluded from the analysis. We will adjust the model for period of time (period 0 through period 5) as a fixed effect to adjust for general calendar trends in RVUs. We will also adjust the model for the type of image ordered at the initial index visit as a categorical variable (plain film, CT, MR).

Aim 1b: To determine if inserting epidemiological evidence decreases subsequent opioid prescriptions.

Hypothesis 1b: Clinics that are randomly assigned to receive the modified imaging reports will have a lower rate of subsequent opioid prescriptions than clinics whose patients do not receive modified reports.

Our pilot work suggested that including epidemiological evidence was associated with a nearly 3.5-fold reduction in opioid prescriptions. We feel that it is important to test this hypothesis given the growing recognition that the overuse of opioids is an important issue in the management of back pain patients.

We will calculate a binomial outcome of the number of patients (out of the eligible patient panel) with a prescription for opioids that occurred after the lumbar spine imaging report was finalized. We will examine this for each primary care provider across each of the six patient accrual periods. We will utilize a generalized linear mixed model to assess the impact of the LIRE intervention on subsequent written opioid prescription rates. Since some patients will already have an active prescription for opioids at the time of the index visit, we will also conduct a subgroup analysis by conducting analyses separately for those patients with and without an active prescription at baseline. As described earlier, we will convert all prescriptions into MEDs. This will allow us to examine temporal trends in not only prescriptions but also dose.

Aim 1c: To determine if inserting epidemiological evidence decreases subsequent cross-sectional imaging (magnetic resonance (MR) and computed tomography).

Hypothesis 1c: Clinics that are randomly assigned to receive the modified imaging reports will have a lower rate of subsequent cross-sectional imaging than clinics whose patients do not receive modified reports.

The number of patients who receive cross-sectional re-imaging within 1 and 2 years (out of the eligible patient panel) will be calculated as a binomial outcome measure for each primary care provider across each of the six patient accrual periods. We will again utilize a generalized linear mixed model to assess the impact of the LIRE intervention on subsequent rates of cross-sectional reimaging, including random effects for the baseline rate of cross-sectional re-imaging and for an indicator of exposure to the LIRE intervention. We will adjust the model for period of time (period 0 through period 5) as a fixed effect to adjust for general longitudinal trends in re-imaging rates. We will also adjust the model for the type of image ordered at the initial index visit as a categorical variable (plain film, CT, MR).

Aim 1d: To explore whether adding epidemiological evidence decreases overall costs of care for low back pain based on CMS reimbursement.

Hypothesis 1d: Clinics that are randomly assigned to receive the modified imaging reports will have lower back pain-related estimated payer costs than clinics whose patients do not receive modified reports.

We will apply CMS-based reimbursement amounts to RVU calculations in order to standardize unit cost estimation among our sites. We will use CPT and diagnostic codes to determine whether interventions are associated with back pain, and multiply the total back-treatment related RVUs at sites by the unit price payment amounts for respective RVU calculations.

Table 5: Sample of RVUs and CMS-based payment amounts for lumbar imaging (US$ 2012)

CPT	Diagnostic Imaging Exam	Hospital (facility)		Professional		
		Hosp RVU	Hosp payment	Pro wRVU	Pro tRVU	Pro payment
72100	2 view x-ray exam lower	0.6399	$48.22	0.22	0.34	$12.08

	spine					
72120	4 view x-ray exam lower spine	0.6399	$48.22	0.22	0.35	$12.47
72131	CT lumbar spine w/o dye	2.746	$206.54	1	1.44	$51.57
72132	CT lumbar spine w/dye	4.2918	$323.42	1.22	1.75	$62.58
72133	CT lumbar spine w/o & w/dye	4.7716	$359.59	1.27	1.82	$65.10
72148	MRI lumbar spine w/o dye	4.8333	$364.24	1.48	2.14	$76.44
72149	MRI lumbar spine w/dye	6.2248	$469.10	1.78	2.57	$91.88
72158	MRI lumbar spine w/o & w/dye	7.6273	$574.79	2.36	3.41	$121.79

Aim 2: To determine whether inserting age-specific prevalence of imaging findings in asymptomatic subjects has a differential effect on subsequent back-related interventions if inserted into lumbar spine magnetic resonance (MR) and computed tomography (CT) imaging reports compared with plain films.

Hypothesis 2: Inserting epidemiological information into plain film reports will result in a greater decrease in subsequent back-related interventions than similar information put into MR and CT reports.

Our pilot work examined only MR. However, given that plain films are generally obtained earlier in the course of back pain and more frequently than cross-sectional imaging, the potential impact of inserting epidemiological information into plain film reports is large. In each separate analysis of Aim 1 outcomes, we will add an indicator of imaging modality (plain film versus MR versus CT) and an imaging modality by treatment interaction term in the model. Primary inference will be on the interaction term, where we expect that patients with more advanced imaging will have a significantly greater reduction in subsequent interventions than those who receive a plain film image.

Aim 3: To determine whether the presence of certain imaging findings influence subsequent interventions

Hypothesis 3: Inserting epidemiological information will result in a greater decrease in subsequent interventions for patients without findings that are clearly clinically important compared with patients who have clinically important imaging findings.

Our work and others have shown that certain imaging findings are likely clinically more important than others (e.g. nerve root compression, moderate to severe central stenosis, disc extrusions). To address this hypothesis, we will use the returned result from radiology to categorize imaging findings into clinically important versus not clinically important. We have identified central canal stenosis, nerve root compression and disc extrusion (a type of herniation) as the clinically most important imaging findings. This is in contrast to findings that are less clinically important (disc bulge, disc narrowing, Modic change, annular fissure, etc).

In each separate analysis of Aim 1 outcomes, we will add an indicator of clinical importance and a clinical importance by treatment interaction term in the model. Similar to Aim 2, primary inference will

again be on the interaction term, where we expect that patients without these more important findings will have a significantly greater reduction in subsequent interventions compared to patients with important findings.

Table 6: Milestones for UH3 (Implementation Phase)- Years 2-5

Year of Project	UH3 Milestone	Comment
**Year 2**	Final testing of intervention deployment	To be completed before Wave 1 of randomization scheduled for April, 2014
	Intervention implemented at 40% of clinical sites	Planned staggered implementation using stepped wedge design will require close monitoring of progress.
	Algorithm finalized for electronic medical record extraction and tested at all sites	This will continue the work started as UH2 Milestone #4. Each site will require a customized algorithm- hence the need for site-specific development and testing
	Protocol paper submitted for publication	We will prepare a manuscript describing our study protocol and procedures.
**Year 3**	Randomized intervention implemented at 80% of clinical sites	Planned staggered implementation using stepped wedge design will require close monitoring of progress.
	Medical record extraction complete for 12mo outcomes on randomization waves 1-2	Data extraction ongoing for duration of project for 12 and 24mo time-points.
	Comparison of abstraction methods for radiology reports (natural language processing vs. Amazon Turk)	
**Year 4**	Intervention implementation completed	All clinical sites randomized to intervention by this time.
	Medical record extraction complete for 12mo outcomes on randomization waves 3-4 and 24mo outcomes on randomization waves 1-2	
	Abstraction of radiology reports through 12mo for waves 1-4 using preferred method from yr 3	
**Year 5**	Medical record extraction including imaging reports complete for 12mo outcomes on randomization wave 5 and 24 mo outcomes on randomization waves 3-4	

	Data analysis, manuscript writing & dissemination of results at national meetings	Manuscripts for 12mo outcomes and 24mo outcomes submitted for publication

Table 7: Timeline for the UH3 Phase (Years 2-5)

	YEAR 2 (2014)												YEAR 3 (2015)												YEAR 4 (2016)												YEAR 5 (2017)											
	J	F	M	A	M	J	J	A	S	O	N	D	J	F	M	A	M	J	J	A	S	O	N	D	J	F	M	A	M	J	J	A	S	O	N	D	J	F	M	A	M	J	J	A	S	O	N	D
**Randomization Wave***																																																
Wave #1																																																
Wave #2																																																
Wave#3																																																
Wave #4																																																
Wave #5																																																
**Validation datasets****																																																
Validation sets transferred from sites to DCC							✖						✖						✖						✖						✖						✖											
**Comprehensive datasets*****																																																
Comprehensive datasets transferred from sites to DCC										✖		✖						✖						✖						✖						✖												
**Data Quality Assessment**																																																
Quality Assessment on set #1										✖																																						
Quality Assessment between set #1 and #2												✖																																				
**Data Safety & Monitoring Reports**																																																
Reports every six monthes																			⊙						⊙						⊙						⊙						⊙					

*Insertion of Intervention text , randomized at clinic level

**Index file with CPT codes, LIRE IDs, radiology image reports verifying insertion of intervention text

***Electronic Medical Record and administrative/billing data for 12mo and 24mo outcomes

6. Statistical Considerations

For details regarding sample size and power calculations as well as all other statistical considerations, please refer to the LIRE Statistical Analysis Plan.

7. Human Subjects

Because the intervention will be administered at the clinic level, consent of either individual patients or primary caregivers is neither feasible nor warranted. Moreover, the intervention is relatively benign (the insertion of additional epidemiological information into the radiology report) and poses minimal risk to caregivers and patients. Because leadership at the Healthcare Systems making up the performance sites are enthusiastic about incorporating the epidemiological benchmarks into their reports and may well eventually adopt them regardless of the project, our study simply allows us to systematically study the effects of a well-controlled implementation of the insertion of the benchmark information. The randomization scheme defines when each clinic begins including the epidemiological information into the reports, with all sites eventually receiving the intervention of interest.

7.1 Human Subjects Involvement and Characteristics

Eligibility criteria: A patient will be eligible for inclusion in the study if they are at least 18 years old and referred by their primary care provider for plain films, CT or MR of the lumbar spine to evaluate low back or leg pain. We will access patient medical records 6 months prior to the index image and for two years after the index image in order to track patient outcomes before and after the intervention. Subjects will receive usual care, and neither their diagnostic evaluation nor their therapy will be

constrained by study considerations. We anticipate enrolling ~100,000, patients who underwent lumbar spine imaging examinations across four different health systems.

7.2 Research Data

Research data will consist of individual subjects' medical record data and information on clinics and providers. We will collect all data passively with automated data extractions. Data extracted from the medical record will include demographic data, variables related to imaging, pharmacy, procedures, hospitalizations, and other factors related to healthcare utilization. We will not collect patient reported outcomes unless they are part of the medical record.

We will collect demographic data on primary care providers and will code the data in such a way that an individual practitioner is not identifiable. We will use patient data to derive pre and post randomization rates of spine related interventions (diagnostic imaging, opioid prescriptions, spine related procedures, physical therapy etc.) among a provider's patient panel.

We will code with a unique study identification number, without reference to patient or provider identity. The code key will be kept secured at the recruitment site, separate from the data. Only the site researchers will have access to the code key (not the researchers at the DCC).

7.3 Potential risks

The research activities in this trial are very low risk. Perhaps the most important risk is a breach of confidentiality of clinical information.

Individual subjects will not be contacted or consented for this project. No patient reported outcomes are being collected and so no patient interviews will be performed. The intervention is being administered at the clinic level; therefore, consent of either individual patients or providers is neither feasible nor warranted. Moreover, the intervention is relatively benign (the insertion of epidemiological benchmark data into the radiology report) and poses virtually no risk to either providers or patients. We will not constrain the choice of tests or treatments offered to subjects.

The main risk associated with this project will be loss of confidentiality as medical record access will be necessary in order to assess the impact of the intervention. We will make extensive efforts to assure that records are kept in locked files and are not identifiable to anyone but the investigators. All PHI will be stored securely locally. Non-PHI data will be uploaded via a web-based system to the Data Coordinating Center at the Center for Biomedical Informatics and Biomedical Statistics at the University of Washington. Anonymized data will be stored on a server located at Biomedical Informatics, where no names or hospital numbers are included and only study numbers will be attached to the data files. Data will be kept on a server that requires a password for entry and in a locked office.

As the identities and clinical information gathered on patients will be guarded, so too, will the identities and data collected on clinic providers. All identifying information will be stored securely at the local recruitment sites. Only coded, limited data set will be transferred to the DCC such that an individual provider from a given clinic within a health system; cannot be identified.

7.4 Adequacy of Protection Against Risks

We anticipate that each site will work within their own health system to identify primary care clinics,

primary care providers, and operationalize the technical aspects regarding the intervention. The intervention itself is the addition of epidemiologic data relevant to the imaging modality and age range (in deciles) of a given patient for whom a radiologic image was ordered. This data will be automatically added to existing template radiology reports in the intervention group. The randomization schedule will be allocated at the clinic site rather than at the individual patient or provider level. Only group results will be reported.

Per Health and Human Services Policy for Protection of Human Research Subjects, Section 46.102.i: "Minimal risk means that the probability and magnitude of harm or discomfort anticipated in the research are not greater in and of themselves than those ordinarily encountered in daily life or during the performance of routine physical or psychological examinations or tests".

We will seek a waiver of consent from the IRB's at each of the participating health care systems since the risk to individuals is minimal, the intervention is relatively benign and consent of patients and providers is not practical.

7.5 Potential Benefits of the Proposed Research to the Subjects
We believe that the risks to subjects are minimal and that the relevant knowledge gains may be great. Individual subjects in this study are not likely to benefit immediately from this new knowledge, although it could influence their subsequent treatment, and may influence the treatment of others with a similar condition. Knowledge of benefit or lack thereof) will inform providers and patients in the future about the usefulness providing epidemiologic context to radiologic results in the management of low back pain.

7.6 Importance o the Knowledge to be Gained
This study will assess the impact epidemiologic data tailored to radiologic modality and age range of a given patient) has on treatment outcomes among those with low back pain in the primary care setting. Low back pain is prevalent, imaging is routinely used in its assessment and evaluation, and radiologic results can heavily inform providers' clinical decision making. Since the risks to research subjects are minor and there is the potential for improved patient management, this research should be pursued.

All implementation sites now have IRB approval in place. Mayo Clinic and Henry Ford each went through their own IRB approval process for minimal risk applications and received approval. Group Health Cooperative has agreed to the IRB of record for the study and UW and Kaiser's IRB's both ceded authority to them for monitoring the study moving forward.

8.0 Data and Safety Monitoring

We have drafted a data safety and monitoring plan (DSMP) and have designated two Safety Officers, Steven Atlas, MD and Constantine Gatsonis, PhD who has agreed to review study data at regular intervals for safety concerns.

References

1. Lidgren L. The bone and joint decade 2000-2010. Bull World Health Organ. 2003;81(9):629. PMCID: 2572548.
2. Initial National Priorities for Comparative Effectiveness Research. Washington DC: Institute of Medicine; 2009 June 30, 2009 Contract No.: Document Number|.
3. Schappert SM, Rechtsteiner EA. Ambulatory medical care utilization estimates for 2006. Natl Health Stat Report. 2008(8):1-29.
4. Owens DK, Qaseem A, Chou R, Shekelle P. High-value, cost-conscious health care: concepts for clinicians to evaluate the benefits, harms, and costs of medical interventions. Ann Intern Med.154(3):174-80.
5. Chou R, Qaseem A, Owens DK, Shekelle P. Diagnostic imaging for low back pain: advice for high-value health care from the American College of Physicians. Ann Intern Med.154(3):181-9.
6. Foundation ABoIM, Physicians ACo. Choosing Wisely Campaign. 2012 [updated 2012; cited]; Available from: choosingwisely.org/.
7. Martin BI, Deyo RA, Mirza SK, Turner JA, Comstock BA, Hollingworth W, et al. Expenditures and health status among adults with back and neck problems. JAMA. 2008;299(6):656-64.
8. Jarvik JJ, Hollingworth W, Heagerty P, Haynor DR, Deyo RA. The Longitudinal Assessment of Imaging and Disability of the Back (LAIDBack) Study: baseline data. Spine. 2001;26(10):1158-66.
9. Boden SD, Davis DO, Dina TS, Patronas NJ, Wiesel SW. Abnormal magnetic-resonance scans of the lumbar spine in asymptomatic subjects. A prospective investigation. J Bone Joint Surg [Am]. 1990;72(3):403-8.
10. Jensen MC, Brant-Zawadzki MN, Obuchowski N, Modic MT, Malkasian D, Ross JS. Magnetic resonance imaging of the lumbar spine in people without back pain [see comments]. N Engl J Med. 1994;331(2):69-73.
11. Jarvik JG, Hollingworth W, Martin B, Emerson SS, Gray DT, Overman S, et al. Rapid magnetic resonance imaging vs radiographs for patients with low back pain: a randomized controlled trial. JAMA. 2003;289(21):2810-8.
12. Lurie JD, Birkmeyer NJ, Weinstein JN. Rates of advanced spinal imaging and spine surgery. Spine (Phila Pa 1976). 2003;28(6):616-20.
13. Aberle DR, Adams AM, Berg CD, Black WC, Clapp JD, Fagerstrom RM, et al. Reduced lung-cancer mortality with low-dose computed tomographic screening. N Engl J Med. 2011;365(5):395-409.
14. Johnson PT, Horton KM, Megibow AJ, Jeffrey RB, Fishman EK. Common incidental findings on MDCT: survey of radiologist recommendations for patient management. J Am Coll Radiol. 2011;8(11):762-7.
15. Iwabuchi Y, Hanamure Y, Ueno K, Fukuda K, Furuta S. Clinical significance of asymptomatic sinus abnormalities on magnetic resonance imaging. Arch Otolaryngol Head Neck Surg. 1997;123(6):602-4.
16. Havas TE, Motbey JA, Gullane PJ. Prevalence of incidental abnormalities on computed tomographic scans of the paranasal sinuses. Arch Otolaryngol Head Neck Surg. 1988;114(8):856-9.
17. Roland M, van Tulder M. Should radiologists change the way they report plain radiography of the spine? Lancet. 1998;352(9123):229-30.

18. Modic MT, Obuchowski NA, Ross JS, Brant-Zawadzki MN, Grooff PN, Mazanec DJ, et al. Acute low back pain and radiculopathy: MR imaging findings and their prognostic role and effect on outcome. Radiology. 2005;237(2):597-604.
19. Kendrick D, Fielding K, Bentley E, Kerslake R, Miller P, Pringle M. Radiography of the lumbar spine in primary care patients with low back pain: randomised controlled trial. BMJ. 2001;322(7283):400-5. PMCID: 26570.
20. Kerry S, Hilton S, Dundas D, Rink E, Oakeshott P. Radiography for low back pain: a randomised controlled trial and observational study in primary care. Br J Gen Pract. 2002;52(479):469-74. PMCID: 1314322.
21. Deyo RA, Diehl AK, Rosenthal M. Reducing roentgenography use. Can patient expectations be altered? Arch Intern Med. 1987;147(1):141-5.
22. Gilbert FJ, Grant AM, Gillan MG, Vale LD, Campbell MK, Scott NW, et al. Low back pain: influence of early MR imaging or CT on treatment and outcome--multicenter randomized trial. Radiology. 2004;231(2):343-51.
23. Ash LM, Modic MT, Obuchowski NA, Ross JS, Brant-Zawadzki MN, Grooff PN. Effects of diagnostic information, per se, on patient outcomes in acute radiculopathy and low back pain. AJNR Am J Neuroradiol. 2008;29(6):1098-103.
24. Chou R, Qaseem A, Owens DK, Shekelle P. Diagnostic imaging for low back pain: advice for high-value health care from the American College of Physicians. Ann Intern Med. 2011;154(3):181-9.
25. Deyo RA, Mirza SK, Turner JA, Martin BI. Overtreating chronic back pain: time to back off? J Am Board Fam Med. 2009;22(1):62-8. PMCID: 2729142.
26. Friedly J, Chan L, Deyo R. Increases in lumbosacral injections in the Medicare population: 1994 to 2001. Spine (Phila Pa 1976). 2007;32(16):1754-60.
27. Luo X, Pietrobon R, Hey L. Patterns and trends in opioid use among individuals with back pain in the United States. Spine (Phila Pa 1976). 2004;29(8):884-90; discussion 91.
28. Deshpande A, Furlan A, Mailis-Gagnon A, Atlas S, Turk D. Opioids for chronic low-back pain. Cochrane Database Syst Rev. 2007(3):CD004959.
29. Airaksinen O, Brox JI, Cedraschi C, Hildebrandt J, Klaber-Moffett J, Kovacs F, et al. Chapter 4. European guidelines for the management of chronic nonspecific low back pain. Eur Spine J. 2006;15 Suppl 2:S192-300.
30. Deyo RA, Gray DT, Kreuter W, Mirza S, Martin BI. United States trends in lumbar fusion surgery for degenerative conditions. Spine (Phila Pa 1976). 2005;30(12):1441-5; discussion 6-7.
31. Mirza SK, Deyo RA. Systematic review of randomized trials comparing lumbar fusion surgery to nonoperative care for treatment of chronic back pain. Spine (Phila Pa 1976). 2007;32(7):816-23.
32. Deyo RA, Mirza SK, Martin BI, Kreuter W, Goodman DC, Jarvik JG. Trends, major medical complications, and charges associated with surgery for lumbar spinal stenosis in older adults. JAMA. 2010;303(13):1259-65. PMCID: 2885954.
33. Martin BI, Turner JA, Mirza SK, Lee MJ, Comstock BA, Deyo RA. Trends in health care expenditures, utilization, and health status among US adults with spine problems, 1997-2006. Spine (Phila Pa 1976). 2009;34(19):2077-84.
34. Bartleson JD. Evidence for and against the use of opioid analgesics for chronic nonmalignant low back pain: a review. Pain Med. 2002;3(3):260-71.
35. Paulozzi LJ, Ryan GW. Opioid analgesics and rates of fatal drug poisoning in the United States. Am J Prev Med. 2006;31(6):506-11.

36. McCullough BJ, Johnson GR, Martin BI, Jarvik JG. Lumbar MR imaging and reporting epidemiology: do epidemiologic data in reports affect clinical management? Radiology. 2012;262(3):941-6. PMCID: 3285226.
37. Scholich SL, Hallner D, Wittenberg RH, Hasenbring MI, Rusu AC. The relationship between pain, disability, quality of life and cognitive-behavioural factors in chronic back pain. Disabil Rehabil.
38. Bick D, Dimmock D. Whole exome and whole genome sequencing. Curr Opin Pediatr.23(6):594-600.
39. Majewski J, Schwartzentruber J, Lalonde E, Montpetit A, Jabado N. What can exome sequencing do for you? J Med Genet.48(9):580-9.
40. Brown CA, Lilford RJ. The stepped wedge trial design: a systematic review. BMC Med Res Methodol. 2006;6:54. PMCID: 1636652.
41. Hussey MA, Hughes JP. Design and analysis of stepped wedge cluster randomized trials. Contemp Clin Trials. 2007;28(2):182-91.
42. Coventry MB, Ghormley RK, Kernohan JW. Intervertebral disc; its microscopic anatomy and pathology; anatomy, development, and physiology. J Bone Joint Surg. 1945;27:105.
43. McRae DL. Asymptomatic intervertebral disc protrusions. Acta Radiologica. 1956;46:9-27.
44. Hitselberger WE, Witten RM. Abnormal myelograms in asymptomatic patients. J Neurosurg. 1968;28(3):204-6.
45. Wiesel S, Tsourmas N, Feffer H, Citrin C, Patronas N. A study of computer-assisted tomography. I. The incidence of positive CAT scans in an asymptomatic group of patients. Spine. 1984;9:549-51.
46. Powell MC, Wilson M, Szypryt P, Symonds EM, Worthington BS. Prevalence of lumbar disc degeneration observed by magnetic resonance in symptomless women. Lancet. 1986;2(8520):1366-7.
47. Weinreb JC, Wolbarsht LB, Cohen JM, Brown CE, Maravilla KR. Prevalence of lumbosacral intervertebral disk abnormalities on MR images in pregnant and asymptomatic nonpregnant women. Radiology. 1989;170(1 Pt 1):125-8.
48. Szypryt EP, Twining P, Mulholland RC, Worthington BS. The prevalence of disc degeneration associated with neural arch defects of the lumbar spine assessed by magnetic resonance imaging. Spine. 1989;14(9):977-81.
49. Tertti MO, Salminen JJ, Paajanen HE, Terho PH, Kormano MJ. Low-back pain and disk degeneration in children: a case-control MR imaging study. Radiology. 1991;180(2):503-7.
50. Bartolozzi C, Caramella D, Zampa V, Dal Pozzo G, Tinacci E, Balducci F. [The incidence of disk changes in volleyball players. The magnetic resonance findings]. Radiol Med (Torino). 1991;82(6):757-60.
51. Buirski G, Silberstein M. The symptomatic lumbar disc in patients with low-back pain. Magnetic resonance imaging appearances in both a symptomatic and control population. Spine. 1993;18(13):1808-11.
52. Parkkola R, Rytokoski U, Kormano M. Magnetic resonance imaging of the discs and trunk muscles in patients with chronic low back pain and healthy control subjects. Spine. 1993;18(7):830-6.
53. Boos N, Rieder R, Schade V, Spratt KF, Semmer N, Aebi M. 1995 Volvo Award in clinical sciences. The diagnostic accuracy of magnetic resonance imaging, work perception, and psychosocial factors in identifying symptomatic disc herniations. Spine. 1995;20(24):2613-25.
54. Salminen JJ, Erkintalo M, Laine M, Pentti J. Low back pain in the young. A prospective three-year follow-up study of subjects with and without low back pain. Spine. 1995;20(19):2101-7; discussion 8.

55. Burns JW, Loecker TH, Fischer JR, Jr., Bauer DH. Prevalence and significance of spinal disc abnormalities in an asymptomatic acceleration subject panel. Aviat Space Environ Med. 1996;67(9):849-53.
56. Paajanen H, Erkintalo M, Parkkola R, Salminen J, Kormano M. Age-dependent correlation of low-back pain and lumbar disc regeneration. Arch Orthop Trauma Surg. 1997;116(1-2):106-7.
57. Savage RA, Whitehouse GH, Roberts N. The relationship between the magnetic resonance imaging appearance of the lumbar spine and low back pain, age and occupation in males. Eur Spine J. 1997;6(2):106-14.
58. Stadnik TW, Lee RR, Coen HL, Neirynck EC, Buisseret TS, Osteaux MJ. Annular tears and disk herniation: prevalence and contrast enhancement on MR images in the absence of low back pain or sciatica. Radiology. 1998;206(1):49-55.
59. Weishaupt D, Zanetti M, Hodler J, Boos N. MR imaging of the lumbar spine: prevalence of intervertebral disk extrusion and sequestration, nerve root compression, end plate abnormalities, and osteoarthritis of the facet joints in asymptomatic volunteers. Radiology. 1998;209(3):661-6.
60. Rao VM, Levin DC, Parker L, Frangos AJ, Sunshine JH. Trends in utilization rates of the various imaging modalities in emergency departments: nationwide Medicare data from 2000 to 2008. J Am Coll Radiol. 2011;8(10):706-9.
61. Picano E. Sustainability of medical imaging. BMJ. 2004;328:578-80.
62. Fineberg H. Computerized cranial tomography: effect on diagnostic and therapeutic plans. JAMA. 1977;38:224-7.
63. Fryback D, Thornbury J. The efficacy of diagnostic imaging. Med Decis Making. 1991;11:88-94.
64. Sox HJ, Margulies I, Sox C. Psychologically mediated effects of diagnostic tests. Ann Intern Med. 1981;95:680-5.
65. Franklin GM, Stover BD, Turner JA, Fulton-Kehoe D, Wickizer TM. Early opioid prescription and subsequent disability among workers with back injuries: the Disability Risk Identification Study Cohort. Spine (Phila Pa 1976). 2008;33(2):199-204.
66. Franklin GM, Mai J, Turner J, Sullivan M, Wickizer T, Fulton-Kehoe D. Bending the prescription opioid dosing and mortality curves: impact of the Washington State opioid dosing guideline. Am J Ind Med.55(4):325-31.
67. Kallmes DF, Comstock BA, Heagerty PJ, Turner JA, Wilson DJ, Diamond TH, et al. A randomized trial of vertebroplasty for osteoporotic spinal fractures. N Engl J Med. 2009;361(6):569-79.
68. Jarvik JG, Comstock BA, Kliot M, Turner JA, Chan L, Heagerty PJ, et al. Surgery versus non-surgical therapy for carpal tunnel syndrome: a randomised parallel-group trial. Lancet. 2009;374(9695):1074-81.
69. Wei LJ, Lachin JM. Properties of the urn randomization in clinical trials. Control Clin Trials. 1988;9(4):345-64.
70. Health WK. Drug facts and compaarisons; 2004 Contract No.: Document Number|.
71. Efron B, Stein C. The jackknife estimate of variance. Annals of Statistics. 1981;9(3):586-96.
72. Briggs A, Claxton K, Sculpher M. Decision modeling for health economic evaluation. Oxford: Oxford University Press; 2008.

Appendices

Appendix A: Article Extraction form (Working Group 1)

Appendix B: Literature Search and Articles used in Intervention text (Working Group 1)

Appendix C: Pilot Implementation Site Checklist (Working Group 2)

Appendix D: Literature Search for RVU-based Assessment (Working Group 3)

LIRE Article Abstraction Form

Abstractor Name:
Lead author: Journal: Year:
1. Review Article __☐__yes (do not abstract prevalence data) __☐__no
2. Clearly asymptomatic LBP population: ☐no (do not abstract) ☐yes ☐ambiguous
3. Article not relevant for other reason: ☐yes (do not abstract) ☐no

Prevalence of Imaging Findings in People Without Low Back Pain (include point estimate and if available CI)							
Finding	Overall Mean Age<br><br>range	Age range	Age range	Age range	Age range	Age range	Age range
# subjects without LBP							
Disc degeneration							
Disc signal loss							
Disc height loss							
Disc bulge							
Disc protrusion							
Annular fissure							
Modic 1 change							
Modic 2 change							
Facet degeneration (any)							
Facet degeneration (mod-sev)							
Other 1							
Other 2							

Comments (e.g. population characteristics, potential problems using data):

LIRE literature search strategy for Intervention Text

1. The LIRE WG1 team worked with University of Washington librarians to develop our search strategy and list of search terms.

2. Using PubMed, we used the following terms for our initial WG1 literature search:

("Morbidity"[Mesh] OR ("epidemiology"[Subheading] OR "epidemiology"[All Fields] OR "prevalence"[All Fields] OR "prevalence"[MeSH Terms]) OR "Epidemiology"[Mesh] OR "epidemiology"[Subheading] OR "Epidemiologic Factors"[Mesh] OR "Incidental Findings"[Mesh] OR incidental[All Fields] OR "Asymptomatic Diseases"[Mesh] OR asymptomatic[All Fields] OR "Unnecessary Procedures"[MeSH Terms]) AND ("Lumbar Vertebrae"[Mesh] OR "Low Back Pain"[Mesh] OR "Intervertebral Disc Displacement"[Mesh] OR "Sciatica"[Mesh] OR "Spinal Stenosis"[Mesh] OR "Synovial Cyst"[Mesh] OR "Scoliosis"[Mesh] OR "Spondylolysis"[Mesh] OR "Spinal Osteochondrosis"[Mesh] OR "disc degeneration"[All Fields] OR "disc height loss"[All Fields] OR "disc bulge"[All Fields] OR "disc protrusion"[All Fields] OR annular[All Fields] OR anular[All Fields] OR "high intensity zone"[All Fields] OR anulus[All Fields] OR annulus[All Fields] OR listhesis[All Fields] OR (disc[All Fields] AND ("desiccation"[MeSH Terms] OR "desiccation"[All Fields] OR "dessication"[All Fields])) OR "disc dehydration"[All Fields] OR (modic[All Fields] AND endplate[All Fields] AND ("Change"[Journal] OR "change"[All Fields])) OR "nerve root displacement"[All Fields] OR "nerve root compression"[All Fields] OR "disc sequestration"[All Fields] OR "intravertebral herniations"[All Fields] OR (intravertebral[All Fields] AND ("hernia"[MeSH Terms] OR "hernia"[All Fields])) OR (intradiscal[All Fields] AND ("hernia"[MeSH Terms] OR "hernia"[All Fields])) OR (intradiscal[All Fields] AND ("hernia"[MeSH Terms] OR "hernia"[All Fields] OR "herniation"[All Fields])) OR "intradural herniation"[All Fields] OR (intravertebral[All Fields] AND ("hernia"[MeSH Terms] OR "hernia"[All Fields])) OR (intravertebral[All Fields] AND ("hernia"[MeSH Terms] OR "hernia"[All Fields] OR "herniation"[All Fields])) OR "prolapsed disc"[All Fields] OR "radial fissure"[All Fields]) AND ("Magnetic Resonance Imaging"[Mesh] OR "Tomography, X-Ray Computed"[Mesh] OR "Radiography"[Mesh])

3. Using PubMed advance search tool, the above search criteria generates N=2957 identified articles. LIRE WG1 used the following reviewers and inclusion criteria to evaluate the abstracts.

Start	End	reviewer 1	reviewer 2
1	330	avins	bresnahan
331	660	bresnahan	chen
661	990	chen	deyo
991	1320	deyo	halabi
1321	1650	halabi	jarvik
1651	1980	jarvik	kallmes
1981	2310	kallmes	luetmer
2311	2640	luetmer	turner
2641	2957	turner	avins

Inclusion criteria for articles:

1. article included subjects without low back pain (LBP)

2. listed prevalence of imaging finding in patients without LBP

3. subjects were >=18 (exclude series that were strictly peds)

4. subjects were human and alive (no cadaver or animal studies)

5. imaging study prevalence data was for either MR, CT or plain film

6. prevalence for at least one of the following was included :

4. spinal stenosis,
5. disc bulge,
6. disc protrusion,
7. disc extrusion,
8. disc herniation,
9. disc degeneration,
10. disc dessication (or dehydration),
11. disc height loss,
12. nerve root involvement (contact, displacement or compression),
13. anular fissure (or anular tear or HIZ),
14. spondylolysis,
15. spondylolisthesis,
16. modic change,
17. Schmorl’s node,
18. synovial cyst,
19. osteochondrosis

References for LIRE Intervention Text

1. Boden SD, Davis DO, Dina TS, Patronas NJ, Wiesel SW. Abnormal magnetic-resonance scans of the lumbar spine in asymptomatic subjects. A prospective investigation. J Bone Joint Surg Am. 1990 Mar;72(3):403-8.

2. Boden SD, Riew KD, Yamaguchi K, Branch TP, Schellinger D, Wiesel SW. Orientation of the lumbar facet joints: association with degenerative disc disease. J Bone Joint Surg Am. 1996 Mar;78(3):403-11.

3. Boos N, Rieder R, Schade V, Spratt KF, Semmer N, Aebi M. 1995 Volvo Award in clinical sciences. The diagnostic accuracy of magnetic resonance imaging, work perception, and psychosocial factors in identifying symptomatic disc herniations. Spine (Phila Pa 1976). 1995 Dec 15;20(24):2613-25.

4. Capel A. et al. Magnetic resonance study of lumbar disks in female dancers. Am J Sports Med. 2009 Jun;37(6):1208-13. Epub 2009 May 5.

5. Carragee E, Alamin T, Cheng I, Franklin T, van den Haak E, Hurwitz E. Are first-time episodes of serious LBP associated with new MRI findings? Spine J. 2006 Nov-Dec;6(6):624-35. Epub 2006 Oct 11.

6. Danielson B et al. Axially loaded magnetic resonance image of the lumbar spine in asymptomatic individuals. Spine (Phila Pa 1976). 2001 Dec 1;26(23):2601-6.

7. Dora C et al. The significance of spinal canal dimensions in discriminating symptomatic from asymptomatic disc herniations. Eur Spine J. 2002 Dec;11(6):575-81. Epub 2002 Aug 28.

8. Edmondston et al. MRI evaluation of lumbar spine flexion and extension in asymptomatic individuals. Manual Therapy (2000) 5(3), 158-164

9. Feng T, Zhao P, Liang G. Clinical significance on protruded nucleus pulposus: a comparative study of 44 patients with lumbar intervertebral disc protrusion and 73 asymptomatic control in tridimentional computed tomography. Zhongguo Zhong Xi Yi Jie He Za Zhi [Chinese Journal of Integrated Traditional and Western Medicine]. 2000 May;20(5):347-9.

10. Greenberg JO, Schnell RG. Magnetic resonance imaging of the lumbar spine in asymptomatic adults. Cooperative study--American Society of Neuroimaging. J Neuroimaging. 1991 Feb;1(1):2-7.

11. Hamanishi C, Kawabata T, Yosii T, Tanaka S. Schmorl's nodes on magnetic resonance imaging. Their incidence and clinical relevance. Spine (Phila Pa 1976). 1994 Feb 15;19(4):450-3.

12. Healy JF, Healy BB, Wong WH, Olson EM. Cervical and lumbar MRI in asymptomatic older male lifelong athletes: frequency of degenerative findings. J Comput Assist Tomogr. 1996 Jan-Feb;20(1):107-12.

13. Jarvik JJ, Hollingworth W, Heagerty P, Haynor DR, Deyo RA. The Longitudinal Assessment of Imaging and Disability of the Back (LAIDBack) Study: baseline data. Spine (Phila Pa 1976). 2001 May 15;26(10):1158-66.

14. Jensen MC, Brant-Zawadzki MN, Obuchowski N, Modic MT, Malkasian D, Ross JS. Magnetic resonance imaging of the lumbar spine in people without back pain. N Engl J Med. 1994 Jul 14;331(2):69-73.

15. Kalichman, L, Li L, Hunter DJ, Been E. Association between computed tomography-evaluated lumbar lordosis and features of spinal degeneration, evaluated in supine position. Spine J. 2010 Apr;11(4); 308-15.

16. Kanayama M, Togawa D, Takahashi C, Terai T, Hashimoto T. Cross-sectional magnetic resonance imaging study of lumbar disc degeneration in 200 healthy individuals. J Neurosurg Spine. 2009 Oct;11(4):501-7.

17. Karakida O, Ueda H, Ueda M, Miyasaka T. Diurnal T2 value changes in the lumbar intervertebral discs. Clin Radiol. 2003 May;58(5):389-92.

18. Kjaer P, Leboeuf-Yde C, Korsholm L, Sorensen JS, Bendix T. Magnetic resonance imaging and low back pain in adults: a diagnostic imaging study of 40-year-old men and women. Spine (Phila Pa 1976). 2005 May 15;30(10):1173-80.

19. Ranson et al. Magnetic resonance imaging of the lumbar spine in asymptomatic professional fast bowlers in cricket. J Bone Joint Surg [Br] 2005;87-B:1111-16.

20. Savage RA, Whitehouse GH, Roberts N. The relationship between the magnetic resonance imaging appearance of the lumbar spine and low back pain, age and occupation in males. Eur Spine J. 1997;6(2):106-14.

21. Silcox DH 3rd, Horton WC, Silverstein AM. MRI of lumbar intervertebral discs. Diurnal variations in signal intensities. Spine (Phila Pa 1976). 1995 Apr 1;20(7):807-11; discussion 811-2.

22. Stadnik TW, Lee RR, Coen HL, Neirynck EC, Buisseret TS, Osteaux MJ. Annular tears and disk herniation: prevalence and contrast enhancement on MR images

in the absence of low back pain or sciatica. Radiology. 1998 Jan;206(1):49-55.

23. Szypryt EP, Twining P, Mulholland RC, Worthington BS. The prevalence of disc degeneration associated with neural arch defects of the lumbar spine assessed by magnetic resonance imaging. Spine (Phila Pa 1976). 1989 Sep;14(9):977-81.

24. Takatalo J et al. Association of modic changes, Schmorl's nodes, spondylolytic defects, high-intensity zone lesions, disc herniations, and radial tears with low back symptom severity among young Finnish adults. Spine. 2012 June 15;37(14)1231-9.

25. Weinreb JC, Wolbarsht LB, Cohen JM, Brown CE, Maravilla KR. Prevalence of lumbosacral intervertebral disk abnormalities on MR images in pregnant and asymptomatic nonpregnant women. Radiology. 1989 Jan;170(1 Pt 1):125-8.

26. Weishaupt D, Zanetti M, Hodler J, Boos N. MR imaging of the lumbar spine: prevalence of intervertebral disk extrusion and sequestration, nerve root compression, end plate abnormalities, and osteoarthritis of the facet joints in asymptomatic volunteers. Radiology. 1998 Dec;209(3):661

27. Zobel BB et al. A Magnetic resonance imaging quantification of early lumbar intervertebral disc degeneration in healthy your adults. Spine. 2012 June 15;37(14)1224-30.

LIRE Project
Intervention Pilot Testing Plan April - May 2013

1. LIRE study design overview
LIRE is a cluster randomized trial to study the impact of inserting a text description of age-specific prevalence of imaging findings among asymptomatic subjects, into lumbar spine imaging reports. We aim to study subsequent back-related interventions (narcotic prescriptions, subsequent imaging, injections, surgeries, etc) over the following 1 and 2 years. We are using a stepped wedge design and will randomize each clinic to begin implementing the intervention text at one of 5 pre-specified dates:

April 1, 2014
October 1, 2014
April 1, 2015
October 1, 2015
April 1, 2016

At clinics randomized to receive the intervention, the text will be inserted into the radiology report whenever one of the following CPT codes is generated: 72100, 72110, 72114, 72131, 72132, 72133, 72148, 72149, 72158, 72080 for patients 18 and older. Once the intervention is implemented at a given clinic, it will remain "on" indefinitely unless the study stopped early for reasons of safety or the health system wants to turn it "off" after the study period (April 2018) although this is not anticipated.

2. Pilot test implementation

The goal of this pilot implementation is to verify clinic eligibility for the LIRE study and to demonstrate successful insertion of the LIRE text into radiology reports. We are capturing data in Table 2 of this document that will be included in the UH2 progress report to NIH in our request for funding to transition to the UH3 phase. (So, this is important…!)

Your approach towards the pilot implementation needs to include the following:

- Assess clinic eligibility: for each clinic identified in Table 2, verify the questions in Table 1 and mark in Table 2 whether the site meets or does not meet the inclusion criteria or if you are unsure. Please provide comments if necessary.
- Proof of implementation of intervention text in Section 3 (Options 1 or 2): using dummy records, successfully demonstrate that for an eligible CPT code (and patient age $\geq$ 18) the text has been inserted into the radiology report. An example printout of the text would suffice to show this.
- Complete pilot implementation before **May 31, 2013**. This is a hard deadline as the transition report must be written and submitted to the NIH soon after this.
- Since we do not have IRB approval, this test needs to be conducted in a test environment or by using a dummy case.

Table 1. LIRE clinic inclusion criteria

**Intervention implementation inclusion criteria to verify in pilot test**
Required Inclusion Criteria
1. Can the intervention text be delivered based upon a specific CPT code (**Xray**: 72100, 72110, 72114, 72080; **CT:** 72131, 72132, 72133; **MR**: 72148, 72149, 72158)?
2. Can modality-specific (Xray, CT, MR) intervention text be inserted?
3. Can the intervention text be delivered based upon patient age (patients 18 and older)?
4. Can the intervention text be delivered to clinics on a scheduled basis at the 5 pre-specified dates listed above?
5. Through an electronic medical record or radiology information system data pull, can you verify that the text was inserted into a patient's record with an eligible imaging CPT code?
Must meet one of the following two criteria:
6.1 Can age range-specific (Section 3, Option 1) text be displayed in the radiology report depending on patient age?
6.2 Can tabular information by age (Section 3, Option 2) be displayed in the radiology report?

3. Intervention Pilot Testing Text

One of the following options would be inserted specific to imaging modality indicated by CPT code.

Option 1: Age-specific intervention text

"**Comment**: The following findings are so common in people without low back pain that while we report their presence, they must be interpreted with caution and in the context of the clinical situation (Reference – Jarvik et al, Spine 2001)
Findings: (prevalence in patients age *XX-YY* without low back pain), Disk degeneration (decreased T2 signal, height loss, bulge) (91%), Disk T2 – signal loss (83%), Disk height loss (56%), Disk bulge (64%), Disk protrusion (32%), Annular fissure (38%)"

Option 2: Age-tabulated intervention text

The following MRI findings are so common in people without low back pain that while we report their presence, they must be interpreted with caution and in the context of the clinical situation.

	Disk Degeneration	Disk T2 Signal Loss	Disk Height Loss	Disk Bulge	Disk Protrusion	Annular Fissure
21-30	A_1%	A_2%	A_3%	A_4%	A_5%	A_6%
31-40	B_1%	B_2%	B_3%	B_4%	B_5%	B_6%
41-50	C_1%	C_2%	C_3%	C_4%	C_5%	C_6%
51-60	D_1%	D_2%	D_3%	D_4%	D_5%	D_6%
61-70	E_1%	E_2%	E_3%	E_4%	E_5%	E_6%
71-80	F_1%	F_2%	F_3%	F_4%	F_5%	F_6%
81-90	G_1%	G_2%	G_3%	G_4%	G_5%	G_6%
$\geq$91	H_1%	H_2%	H_3%	H_4%	H_5%	H_6%

4. Group Health clinics identified for the LIRE project. Please verify that the clinic meets the inclusions criteria listed in Table 1.

Table 2. Site eligibility evaluation.

#	Group Health Clinic Name	#PCPs	Meets Inclusion Criteria 1 - 5	Meets Inclusion Criteria 6.1 or 6.2		Reasons for Failure
1	Bellevue Medical Center	12	☐	☐	☐	
2	Burien Medical Center	15	☐	☐	☐	
3	Capitol Hill Campus	32	☐	☐	☐	
4	Downtown Seattle Medical Center	7	☐	☐	☐	
5	Everett Medical Center	20	☐	☐	☐	
6	Factoria Medical Center	12	☐	☐	☐	
7	Federal Way Medical Center	14	☐	☐	☐	
8	Kent Medical Center	7	☐	☐	☐	
9	Spokane-Lidgerwood Medical Center	13	☐	☐	☐	
10	Northgate Medical Center	31	☐	☐	☐	
11	Northshore Medical Center	8	☐	☐	☐	
12	Olympia Medical Center	41	☐	☐	☐	
13	Port Orchard Medical Center	18	☐	☐	☐	
14	Poulsbo Medical Center	8	☐	☐	☐	
15	Puyallup Medical Center	11	☐	☐	☐	
16	Rainier Medical Center	8	☐	☐	☐	
17	Redmond Medical Center	10	☐	☐	☐	
18	Renton Medical Center	13	☐	☐	☐	
19	Spokane-Riverfront Medical Center	17	☐	☐	☐	
20	Silverdale Medical Center	16	☐	☐	☐	
21	Spokane-South Hill Medical Center	5	☐	☐	☐	
22	Tacoma Medical Center	10	☐	☐	☐	
23	Tacoma South Medical Center	15	☐	☐	☐	
24	Spokane-Veradale Medical Center	8	☐	☐	☐	
25	Lynnwood Medical Center	15	☐	☐	☐	

Additional comments from pilot test implementation

<comments here>

LIRE Working Group 3
Appendix of Relevant Articles Using RVU-based Assessment

1. Adelgais KM, Grossman DC, Langer SG, Mann FA. Use of helical computed tomography for imaging the pediatric cervical spine. *Academic emergency medicine : official journal of the Society for Academic Emergency Medicine.* Mar 2004;11(3):228-236.
2. Allen B, Jr., Donovan WD, McGinty G, et al. Professional component payment reductions for diagnostic imaging examinations when more than one service is rendered by the same provider in the same session: an analysis of relevant payment policy. *Journal of the American College of Radiology : JACR.* Sep 2011;8(9):610-616.
3. Appleton P, Chacko A, Rodriguez EK. Financial implications of nonoperative fracture care at an academic trauma center. *Journal of orthopaedic trauma.* Nov 2012;26(11): 617-619.
4. Archila AM, Jarecke AJ, Damiani TN, Boorady J. Tri-service optometry productivity findings and benchmark recommendation. *Military medicine.* Apr 2007;172(4):418-420.
5. Arenson RL, Lu Y, Elliott SC, Jovais C, Avrin DE. Measuring the academic radiologist's clinical productivity: applying RVU adjustment factors. *Academic radiology.* Jun 2001;8(6):533-540.
6. Barnes SL, Robinson BR, Richards JT, et al. The devil is in the details: maximizing revenue for daily trauma care. *Surgery.* Oct 2008;144(4):670-675; discussion 675-676.
7. Becker ER, Hall K. Physician services in an academic neurology department: using the resource-based relative-value scale to examine physician activities. *Journal of health care finance.* Summer 2001;27(4):79-91.
8. Becker ER, Mauldin PD, Culler SD, Kosinski AS, Weintraub WS, King SB. Applying the resource-based relative value scale to the Emory angioplasty versus surgery trial. *The American journal of cardiology.* Mar 15 2000;85(6):685-691.
9. Begaz T, Decker MC, Treat R, Tews M. No relationship between measures of clinical efficiency and teaching effectiveness for emergency medicine faculty. *Emergency medicine journal : EMJ.* Jan 2011;28(1):37-39.
10. Bender JR, Johnson AJ, Schenk TW. The economic value of automotive occupational health services: business metrics for performance management. *Journal of occupational and environmental medicine / American College of Occupational and Environmental Medicine.* Feb 2008;50(2):138-145.
11. Bergersen L, Gauvreau K, McElhinney D, et al. Capture of Complexity of Specialty Care in Pediatric Cardiology by Work RVU Measures. *Pediatrics.* Feb 2013;131(2):258-267.
12. Bernard AM, Hayward RA, Rosevear J, Chun H, McMahon LF. Comparing the hospitalizations of transfer and non-transfer patients in an academic medical center. *Academic medicine : journal of the Association of American Medical Colleges.* Mar 1996;71(3):262-266.
13. Bittner JGt, Kang R, Stern PJ. Management of flexor tendon sheath ganglions: a cost analysis. *The Journal of hand surgery.* Jul 2002;27(4):586-590.
14. Bree RL, Kazerooni EA, Katz SJ. Effect of mandatory radiology consultation on inpatient imaging use. A randomized controlled trial. *JAMA : the journal of the American Medical Association.* Nov 20 1996;276(19):1595-1598.
15. Brennan DF, Silvestri S, Sun JY, Papa L. Progression of emergency medicine resident productivity. *Academic emergency medicine : official journal of the Society for Academic Emergency Medicine.* Sep 2007;14(9):790-794.
16. Bronstein JM, Adams EK. Rural-urban differences in health risks, resource use and expenditures within three state medicaid programs: implications for medicaid managed care. *The Journal of rural health : official journal of the American Rural Health Association and the National Rural Health Care Association.* Winter 2002;18(1):38-48.
17. Buntin MJ, Escarce JJ, Goldman D, Kan H, Laugesen MJ, Shekelle P. Increased Medicare expenditures for physicians' services: what are the causes? *Inquiry : a journal of medical care organization, provision and financing.* Spring 2004;41(1):83-94.

18. Chaudhry R, Kottke TE, Naessens JM, et al. Busy physicians and preventive services for adults. *Mayo Clinic proceedings. Mayo Clinic.* Feb 2000;75(2):156-162.

19. Cheriff AD, Kapur AG, Qiu M, Cole CL. Physician productivity and the ambulatory EHR in a large academic multi-specialty physician group. *International journal of medical informatics.* Jul 2010;79(7):492-500.

20. Chin MH, Zhang JX, Merrell K. Specialty differences in the care of older patients with diabetes. *Medical care.* Feb 2000;38(2):131-140.

21. Chung KC, Walters MR, Greenfield ML, Chernew ME. Endoscopic versus open carpal tunnel release: a cost-effectiveness analysis. *Plastic and reconstructive surgery.* Sep 1998;102(4):1089-1099.

22. Conoley PM, Vernon SW, Burtram SG. Growth profiles for radiology in the United States, 1973-1989: commentary based upon analysis of relative value units. *European journal of radiology.* Oct 1992;15(3):264-269.

23. Conoley PM, Vernon SW, Burtram SG. Productivity of radiologists in the United States by imaging technique: a 16-year analysis based upon relative value units. *European journal of radiology.* Oct 1992;15(3):258-263.

24. Conrad D, Fishman P, Grembowski D, et al. Access intervention in an integrated, prepaid group practice: effects on primary care physician productivity. *Health services research.* Oct 2008;43(5 Pt 2):1888-1905.

25. Cook SP. Candidate's Thesis: Laryngotracheal separation in neurologically impaired children: long-term results. *The Laryngoscope.* Feb 2009;119(2):390-395.

26. Cullen EJ, Jr., Lawless ST, Hertzog JH, et al. A model of determining a fair market value for teaching residents: who profits? *Pediatrics.* Jul 2003;112(1 Pt 1):40-48.

27. Davenport DL, Ferraris VA, Hosokawa P, Henderson WG, Khuri SF, Mentzer RM, Jr. Multivariable predictors of postoperative cardiac adverse events after general and vascular surgery: results from the patient safety in surgery study. *Journal of the American College of Surgeons.* Jun 2007;204(6):1199-1210.

28. Davis KA, Cabbad NC, Schuster KM, et al. Trauma team oversight improves efficiency of care and augments clinical and economic outcomes. *The Journal of trauma.* Dec 2008;65(6):1236-1242; discussion 1242-1234.

29. Davis PL. Assessing the potential versus the actual earnings of academic radiologists: effects of unequal duty service assignments. *Academic radiology.* Aug 2001;8(8): 782-791.

30. Dobrez D, Sasso AL, Holl J, Shalowitz M, Leon S, Budetti P. Estimating the cost of developmental and behavioral screening of preschool children in general pediatric practice. *Pediatrics.* Oct 2001;108(4):913-922.

31. Dorr DA, Wilcox A, McConnell KJ, Burns L, Brunker CP. Productivity enhancement for primary care providers using multicondition care management. *The American journal of managed care.* Jan 2007;13(1):22-28.

32. Dudas RA, Monroe D, McColligan Borger M. Community pediatric hospitalists providing care in the emergency department: an analysis of physician productivity and financial performance. *Pediatric emergency care.* Nov 2011;27(11):1099-1103.

33. Duszak R, Jr., Harris AB. Percutaneous abscess drainage: use of related radiology services and associated economic impact on a radiology practice. *Journal of vascular and interventional radiology : JVIR.* May 2003;14(5):597-601.

34. Fahy BN, Bold RJ, Schneider PD, Khatri V, Goodnight JE, Jr. Cost-benefit analysis of biopsy methods for suspicious mammographic lesions; discussion 994-5. *Archives of surgery (Chicago, Ill. : 1960).* Sep 2001;136(9):990-994.

35. Fairchild DG, McLoughlin KS, Gharib S, et al. Productivity, quality, and patient satisfaction: comparison of part-time and full-time primary care physicians. *Journal of general internal medicine.* Oct 2001;16(10):663-667.

36. Fleming NS, Becker ER, Culler S, Cheng D, McCorkle R, Ballard DJ. Financial performance of primary care physician practices prior to electronic health record implementation. *Proceedings (Baylor University. Medical Center).* Apr 2009;22(2):

112-118.
37. Grace C, Alston WK, Ramundo M, Polish L, Kirkpatrick B, Huston C. The complexity, relative value, and financial worth of curbside consultations in an academic infectious diseases unit. *Clinical infectious diseases : an official publication of the Infectious Diseases Society of America.* Sep 15 2010;51(6):651-655.
38. Green PH, Neugut AI, Naiyer AJ, Edwards ZC, Gabinelle S, Chinburapa V. Economic benefits of increased diagnosis of celiac disease in a national managed care population in the United States. *Journal of insurance medicine (New York, N.Y.).* 2008;40(3-4): 218-228.
39. Griffin TC, Hutter JJ, Johnson KK, Moscow JA. A survey of clinical productivity and current procedural terminology (CPT) coding patterns of pediatric hematologist/ oncologists. *Pediatric blood & cancer.* Aug 2004;43(2):140-147.
40. Henke PK, Kubus J, Englesbe MJ, Harbaugh C, Campbell DA. A statewide consortium of surgical care: a longitudinal investigation of vascular operative procedures at 16 hospitals. *Surgery.* Oct 2010;148(4):883-889; discussion 889-892.
41. Henley MB, Mann FA, Holt S, Marotta J. Trends in case-mix-adjusted use of radiology resources at an urban level 1 trauma center. *AJR. American journal of roentgenology.* Apr 2001;176(4):851-854.
42. Hillman BJ, Olson GT, Griffith PE, et al. Physicians' utilization and charges for outpatient diagnostic imaging in a Medicare population. *JAMA : the journal of the American Medical Association.* Oct 21 1992;268(15):2050-2054.
43. Johnson RG, Arozullah AM, Neumayer L, Henderson WG, Hosokawa P, Khuri SF. Multivariable predictors of postoperative respiratory failure after general and vascular surgery: results from the patient safety in surgery study. *Journal of the American College of Surgeons.* Jun 2007;204(6):1188-1198.
44. Jordan SW, Mioton LM, Smetona J, et al. Resident Involvement Affects Plastic Surgery Outcomes: An Analysis of 10,356 Patients From the NSQIP Database. *Plastic and reconstructive surgery.* Dec 17 2012.
45. Katz SJ, McMahon LF, Manning WG. Comparing the use of diagnostic tests in Canadian and US hospitals. *Medical care.* Feb 1996;34(2):117-125.
46. Khandelwal CM, Meyers MO, Yeh JJ, et al. Relative value unit impact of complex skin closures to academic surgical melanoma practices. *American journal of surgery.* Sep 2012;204(3):327-331.
47. Khorasani R, Goel PK, Ma'luf NM, Fox LA, Seltzer SE, Bates DW. Trends in the use of radiology with inpatients: what has changed in a decade? *AJR. American journal of roentgenology.* Apr 1998;170(4):859-861.
48. Kravet SJ, Jones H, Howell EE, Wright SM. Pilot study comparing patients' valuation of health-care services with Medicare's relative value units. *Health expectations : an international journal of public participation in health care and health policy.* Dec 2008;11(4):391-399.
49. Laditka SB, Mastanduno MP, Laditka JN. Health care use of individuals with diabetes in an employer-based insurance population. *Archives of internal medicine.* May 28 2001;161(10):1301-1308.
50. Latimer EA, Verrilli D, Welch WP. Utilization of physician services at the end of life: differences between the United States and Canada. *Inquiry : a journal of medical care organization, provision and financing.* Spring 1999;36(1):90-100.
51. Levin DC, Rao VM, Parker L, Frangos AJ, Sunshine JH. Recent trends in utilization of cardiovascular imaging: how important are they for radiology? *Journal of the American College of Radiology : JACR.* Sep 2005;2(9):736-739.
52. Lind BK, Gerkovich MM, Cherkin DC, Deyo RA, Sherman KJ, Lafferty WE. Effect of Risk Adjustment Method on Comparisons of Health Care Utilization Between Complementary and Alternative Medicine Users and Nonusers. *Journal of alternative and complementary medicine (New York, N.Y.).* Oct 4 2012.
53. Lu Y, Zhao S, Chu PW, Arenson RL. An update survey of academic radiologists' clinical

productivity. *Journal of the American College of Radiology : JACR.* Jul 2008;5(7): 817-826.
54. Lynch F. The use of an interactive computerized daily schedule in a busy interventional radiology practice increases efficiency. *Journal of the American College of Radiology : JACR.* Dec 2004;1(12):965-971.
55. Maitino AJ, Levin DC, Parker L, Rao VM, Sunshine JH. Practice patterns of radiologists and nonradiologists in utilization of noninvasive diagnostic imaging among the Medicare population 1993-1999. *Radiology.* Sep 2003;228(3):795-801.
56. Maxwell S, Zuckerman S, Berenson RA. Use of physicians' services under Medicare's resource-based payments. *The New England journal of medicine.* May 3 2007;356(18): 1853-1861.
57. Mortenson MM, Ho HS, Bold RJ. An analysis of cost and clinical outcome in palliation for advanced pancreatic cancer. *American journal of surgery.* Sep 2005;190(3):406-411.
58. Noll RE, Jr., Tonnessen BH, Kim J, Money SR, Sternbergh WC, 3rd. Long-term postplacement cost comparison of AneuRx and Zenith endografts. *Annals of vascular surgery.* Nov 2008;22(6):710-715.
59. Noll RE, Jr., Tonnessen BH, Mannava K, Money SR, Sternbergh WC, 3rd. Long-term postplacement cost after endovascular aneurysm repair. *Journal of vascular surgery.* Jul 2007;46(1):9-15; discussion 15.
60. Phibbs CS, Bhandari A, Yu W, Barnett PG. Estimating the costs of VA ambulatory care. *Medical care research and review : MCRR.* Sep 2003;60(3 Suppl):54S-73S.
61. Privette AR, Shackford SR, Osler T, Ratliff J, Sartorelli K, Hebert JC. Implementation of resident work hour restrictions is associated with a reduction in mortality and provider-related complications on the surgical service: a concurrent analysis of 14,610 patients. *Annals of surgery.* Aug 2009;250(2):316-321.
62. Raja AS, Mortele KJ, Hanson R, Sodickson AD, Zane R, Khorasani R. Abdominal imaging utilization in the emergency department: trends over two decades. *International journal of emergency medicine.* 2011;4:19.
63. Rubinsky AD, Sun H, Blough DK, et al. AUDIT-C alcohol screening results and postoperative inpatient health care use. *Journal of the American College of Surgeons.* Mar 2012;214(3):296-305 e291.
64. Shine D, Jessen L, Bajaj J, Pencak D, Panush R. Actual and potential effects of medical resident coverage on reimbursement for inpatient visits by attending physicians. *Journal of general internal medicine.* Jun 2002;17(6):428-434.
65. Showstack J, Lin F, Learman LA, et al. Randomized trial of medical treatment versus hysterectomy for abnormal uterine bleeding: resource use in the Medicine or Surgery (Ms) trial. *American journal of obstetrics and gynecology.* Feb 2006;194(2):332-338.
66. Siegel JE, Clancy CM. Relative value in healthcare: cost-effectiveness of interventions. *Journal of nursing care quality.* Apr-Jun 2006;21(2):99-103.
67. Solomon A, Martino S. Relative value units: practical productivity measurement. *Radiology management.* Winter 1991;13(1):33-37.
68. Soremekun OA, Noble VE, Liteplo AS, Brown DF, Zane RD. Financial impact of emergency department ultrasound. *Academic emergency medicine : official journal of the Society for Academic Emergency Medicine.* Jul 2009;16(7):674-680.
69. Talley JD, Mauldin PD, Becker ER, Stikovac M, Leesar MA. Cost and therapeutic modification of intracoronary ultrasound-assisted coronary angioplasty. *The American journal of cardiology.* Jun 15 1996;77(15):1278-1282.
70. Weintraub WS, Culler SD, Kosinski A, et al. Economics, health-related quality of life, and cost-effectiveness methods for the TACTICS (Treat Angina With Aggrastat [tirofiban]] and Determine Cost of Therapy with Invasive or Conservative Strategy)-TIMI 18 trial. *The American journal of cardiology.* Feb 1 1999;83(3):317-322.
71. Welch WP, Verrilli D, Katz SJ, Latimer E. A detailed comparison of physician services for the elderly in the United States and Canada. *JAMA : the journal of the American Medical Association.* May 8 1996;275(18):1410-1416.

72. Wiersema MJ, Mergener K. Current procedural terminology, Resource-based Relative Value Scale, and the Center for Medicare and Medicaid Services: overview. *Gastrointestinal endoscopy clinics of North America.* Oct 2006;16(4):775-787.

73. Williams TR. A geologic survey of the medicare RBRVS system. *Journal of the American College of Radiology : JACR.* Mar 2004;1(3):192-198.

74. Zuber TJ, Purvis JR. Coding and reimbursement of primary care debridement and excision procedures. *The Journal of family practice.* Dec 1992;35(6):663-672.

75. Zwolak RM. Resource-Based Relative Value Scale (RBRVS), coding, and Medicare reimbursement. *Seminars in vascular surgery.* Jun 1997;10(2):119-127.

Research Aims

Aim 1: To determine whether inserting a description of age-specific prevalence of imaging findings among asymptomatic subjects into lumbar spine imaging reports decreases back-related interventions (imaging, injections, surgeries, etc.) over the subsequent year.

> **Aim 1a**: To determine if inserting epidemiological evidence reduces Relative Value Units (RVUs) attributable to spine interventions (imaging, injections, specialist referrals, surgeries, etc.).
>
> **Aim 1b**: To determine if inserting epidemiological data decreases opioid prescriptions.
>
> **Aim 1c**: To determine if inserting epidemiological evidence decreases subsequent cross-sectional imaging (magnetic resonance (MR) and computed tomography (CT)).
>
> **Aim 1d:** To explore whether adding epidemiological evidence decreases overall costs of care for low back pain based on CMS reimbursement.

Aim 2: To determine whether inserting age-specific prevalence of imaging findings in asymptomatic subjects has a differential effect on subsequent back-related interventions if inserted into lumbar spine MR and CT imaging reports compared with plain films.

Aim 3: To determine if specific imaging findings influence subsequent interventions.

Intervention: This trial will study two groups of patients within providers, each will have had a lumbar imaging CPT code. Patients and providers at intervention clinics will receive additional prevalence summary data of incidental findings as a part of their radiology report. Control patients and providers will receive the standard imaging report without the LIRE text.

Design: Using a stepped wedge cluster randomized design[1], we will randomly assign all predetermined clinics at each site to receive the intervention at one of five fixed time-points. Interventions will roll out every six months at the start of the second quarter of UH3 Year 2 according to the schedule shown in Figure 1.

Figure 1: Proposed Randomization Schedule

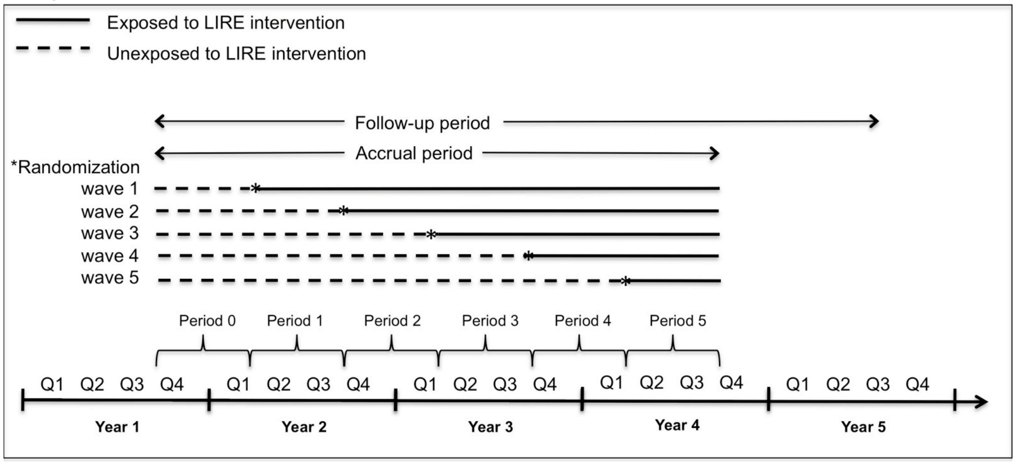

During the UH2 project phase, we obtained a current and accurate enumeration of PCPs within clinics. Within each recruitment site, we sorted clinics by number of primary care providers into tertiles (e.g. small, medium, large clinics). From each tertile we will randomly select clinics using urn-based randomization (without replacement) stratified by site and clinic size such that clinics of small, medium, and large size are equally represented in each randomization wave. Table 1

displays the site-specific strata definitions and size. In total, we will randomize 110 clinics with 1,824 PCPs as units of observation within those clinics. Note that we have chosen to use site-specific definitions for the size of the clinic with the goal of having balance of clinic size within each site. In addition, by balancing randomization on size we will be sure to have comparable time on control and intervention for each clinic size strata.

In the original project application, we assumed 128 clinics and 1,898 PCPs would participate in the LIRE project. After input from the Collaboratory Biostatistics Core, we excluded all clinics with a single PCP (n=18) from the primary study and statistical analysis and will only include clinics with 2 or more PCPs.

Table 1. Within-site stratified randomization schedule of clinics by number of PCPs.

Recruitment Site	Units of Randomization (# of PCPs)	PCP strata size boundaries (# clinics)		
		Small	Medium	Large
Group Health Cooperative	25 (370)	5 to 10 (9)	11 to 15 (8)	16 to 41 (8)
Henry Ford Health System	26 (230)	3 to 6 (9)	7 to 9 (9)	10 to 24 (8)
Kaiser Permanent N. CA	20 (865)	17 to 29 (7)	33 to 39 (5)	43 to 106 (8)
Mayo Clinic Health System	39 (359)	2 to 4 (15)	5 to 9 (12)	11 to 34 (12)
**Total**	**110 (1,824)**			

Primary Outcome: We have devoted substantial effort towards developing and refining the primary outcome measure: a summary back-specific relative value unit (RVU). The back-specific RVU is a composite measure of spine intervention intensity that combines the overall intensity of resource utilization for back pain care into a single metric.

To develop the composite RVU measure, we used data from our large cohort of patients with back pain who comprise the Back pain Outcomes using Longitudinal Data (BOLD) Project, Agency for Healthcare Research and Quality (AHRQ)-funded study. During our work with the BOLD Project we developed algorithms to abstract electronic medical record (EMR) data across three health systems (two of which overlap with LIRE): Kaiser Northern California, Henry Ford Health System and Harvard Vanguard/Harvard Pilgrim. For the 5,239 BOLD cohort participants, we obtained extensive EMR data on pharmacy records, healthcare utilization (CPT codes), diagnoses and provider visits (ICD-9 codes), and inpatient hospitalization data.

Using the Medicare Physician Fee Schedule (http://www.cms.gov/) we generated and tested a mapping algorithm to assign more than 10,000 unique CPT codes to RVUs. A sample of RVUs from the 2012 CMS file is shown in Table 2. Using the BOLD cohort EMR data, we developed and tested an algorithm for aggregating individual RVUs across procedures over a time interval for a given patient, as well as across primary care providers or clinics.

Table 2. Example spine-related CPT codes and associated RVUs.

CPT Code	Description	RVU
72100	X-ray exam of lower spine - 2 views	1.07
97001	PT Evaluation	2.18
99214	Detailed office visit	2.26
99284	Emergency department visit - high moderate intensity	3.37
64483	Epidural injection for lumbar spinal stenosis	3.37
72131	CT lumbar spine w/o dye	6.27
72148	MRI Lumbar Spine w/o Contrast	11.31
63047	Removal of spinal lamina	32.89
22804	Fusion of the spine	71.60

To obtain a spine-related summary RVU from CPT and ICD-9 codes, we used an existing algorithm

developed by a colleague at Dartmouth College.[2-4] Aggregating across CPT codes identified by this algorithm yields the back-specific RVU.

We are currently preparing a manuscript describing this development work as well as a manuscript that directly influences and informs our LIRE UH3 proposal. Using BOLD cohort data, we identified a subset of patients who have had an early lumbar image (MRI/CT or plain film) following an office visit for back pain. Our BOLD cohort manuscript (in preparation) compares the one-year cumulative RVU of early-imaged patients to carefully matched BOLD cohort controls who did not have an early lumbar image. Preliminary results indicate a substantial downstream increase in healthcare utilization for patients who received an early image compared to propensity score matched controls. Patients who underwent a lumbar MRI or CT had a mean one-year RVU of 150 +/- 410, versus 120 +/- 450 for those who had an early plain film, versus 43 +/- 120 for carefully matched controls. Mapping the relative increases of utilization of nearly 80 and 110 RVUs for the plain film and advanced imaging modalities to the example codes shown in Table 2, we see that imaged patients undergo substantially more procedures. Our expectation for the UH3 project is that the insertion of normative prevalence data into lumbar imaging reports will reduce subsequent inappropriate healthcare utilization.

<u>Secondary Outcomes:</u> In addition to back-specific RVU, important secondary outcomes will be obtained and derived using electronic medical record data pulls and include: an indicator of opioid prescriptions written within 30 and 90 days after the index image (Aim 1b); subsequent cross-sectional re-imaging within 90 days and 12 months (Aim 1c); and medical costs (Aim 1d). In the BOLD project, we developed mapping algorithms based upon the United States Food and Drug Administration National Drug Codes (NDC)[5] that generate an indicator of whether or not an individual pharmacy record is an opioid analgesic. Similarly, we have enumerated and categorized a listing of CPT codes that indicate cross-sectional lumbar imaging (CT, or MRI).

<u>General Analytic Strategy</u>: To evaluate the effectiveness of inserting epidemiologic evidence into an imaging report we will use longitudinal regression methods such as linear mixed effects models (LMMs) or generalized linear mixed models (GLMMs) for all primary and secondary outcome measures. Mixed models provide an efficient method for analysis of longitudinal or multilevel data and will be the basis of our primary analysis approach. However, correct model specification is required to ensure valid results when using LMMs or GLMMs and we will therefore use robust standard errors for our primary analysis. Therefore, we are effectively adopting a “working” correlation structure through the specification of flexible multilevel models (LMM or GLMM) but will rely on non-parametrically valid robust standard errors for inference where we cluster on the clinic. Secondary analysis will directly use generalized estimating equations (GEE) adopting simple exchangeable correlation models at the clinic level to determine whether conclusions appear sensitive to model specification.

In each analysis we will also consider a ‘washout period’ in the three months prior to the intervention being activated at a clinic, as determined by the randomization schedule. The rationale for a washout period is to reduce or eliminate within-provider cross-contamination of patient outcomes and utilization in the transition period between control and intervention. Including a washout period reduces the risk of having a patient initially treated in the control time period return to their primary care provider for subsequent care after the primary care provider has been exposed to the intervention through other patients. This reduces the potential bias due to within-provider cross-contamination of outcomes on the estimated intervention effect.

<u>Primary Analysis</u>: The primary longitudinal model for back pain specific RVUs will use a time-varying intervention status indicator $Status_{kt}$ (0 = control, 1 = intervention, for clinic k at time t). Use of the time-dependent intervention status indicator permits both within-clinic contrasts that inform intervention effects (post- versus pre-intervention) as well as contrasts across clinics with different intervention statuses within each time period. The specific regression model will adopt a functional form given below, with fixed effects for time (linear), age (18-39, 40-59, 60+, using two dummy variables), imaging modality type (plain film, CT, MRI using two dummy variables), and clinic size (small, medium, large, using two dummy variables), and site (Group Health Cooperative, Henry Ford, Kaiser Permanente, Mayo Clinic, using three dummy variables) in addition to random

effects for provider, clinic, and intervention status:

$Y_{ijk} = \beta_0 + \beta_1 \cdot Time_t + \beta_2^T \cdot Age_{ijk} + \beta_3^T \cdot Modality_{ijk} + \beta_4^T \cdot Size_k + \beta_5^T \cdot Site_k + \lambda_0 \cdot Status_{kt} +$ *mean model*
$b_{k,0} + b_{k,1} \cdot Status_{kt} +$ *clinic random effects*
$a_{jk,0} + e_{ijk}$ *provider random effects and errors*

We will collect the outcome measure Y_{ijk} on patient i ($i = 1,2,\ldots,n_j$) under primary care provider j ($j = 1,2,\ldots,n_k$) enrolled in time period t ($t = 0,1,2,\ldots ,5$) in order to evaluate the overall effect of the intervention at the level of the clinic k ($k = 1,2,\ldots,110$). Note that we will collect a single outcome measure for each subject recording the total utilization (RVU) over the 12 months after the index imaging event. Given that the random effects structure may contain additional elements (see below) we will use a robust standard error to test the null hypothesis that $\lambda_0 = 0$. For example, using SAS PROC MIXED we can use the "empirical" option in order to obtain robust standard errors. Alternatively, use of the jackknife (at the clinic level) provides a robust standard error estimate (if using R and *lmer*) that is simple to compute.

Figure 2: Hypothetical example of study data showing random clinic intercepts and intervention effects. Each line shows the expected profile for a specific clinic. Here clinic 1 initiates intervention at quarter=2 while clinic 2 initiates at quarter=4.

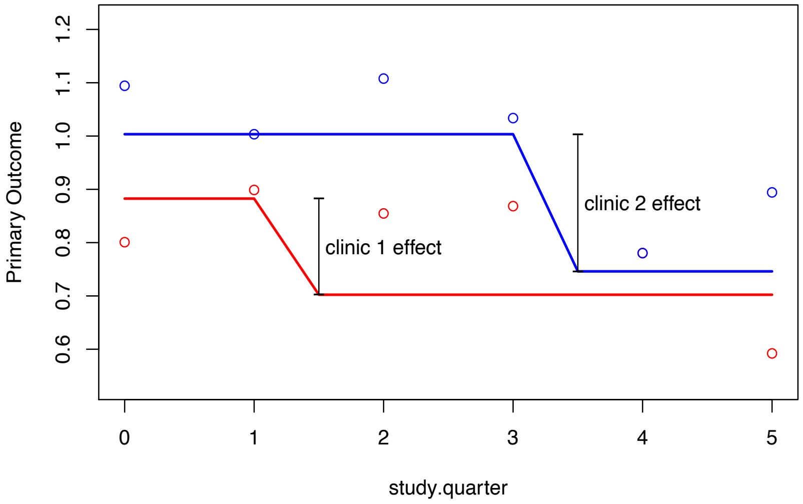

Key Model Parameters: The primary parameter of interest is λ_0, which represents the average effect of the intervention adjusting for temporal trends ($Time_t$), clinic characteristics ($Site_k$, $Size_k$), and individual covariates (Age_{ijk}, $Modality_{ijk}$). In order to interpret the random effects structure we focus on clinic level means removing covariate effects where we have: adjusted mean at clinic k for times prior to intervention = $\beta_0 + b_{k,0}$; and the adjusted mean at clinic k for times after start of the intervention = $\beta_0 + b_{k,0} + \lambda_0 + b_{k,1}$. For clinic-specific means we average over both providers ($a_{jk,0}$) and patients (e_{ijk}). Using this representation we interpret β_0 as the pre-intervention adjusted overall mean outcome averaging across all clinics, and $b_{k,0}$ is the difference between that adjusted overall mean and the pre-intervention (baseline) mean for clinic k. The variance, var($b_{k,0}$), is a measure of the variation in the baseline mean outcome across clinics. The change in the adjusted mean outcome for clinic k is given by: (post-intervention adjusted mean) – (pre-intervention adjusted mean) = $(\beta_0 + b_{k,0} + \lambda_0 + b_{k,1}) - (\beta_0 + b_{k,0}) = \lambda_0 + b_{k,1}$. Here λ_0 represents the average intervention effect across all clinics and $b_{k,1}$ represents the difference between that average intervention effect and the intervention effect for clinic k. The variance, var($b_{k,1}$), is a measure of the variation in the change associated with intervention across clinics, or a measure of the heterogeneity of the intervention effect.

Our primary regression model acknowledges the fundamental multilevel structure of individual-level data collected in health care systems with patients nested within providers, and providers nested within clinics. Although, the basic intervention contrast is the pre-post change associated with the initiation of intervention for each clinic, we do not propose using clinic-level summary measures for inference since the weighting of both patients and providers is not simple when heterogeneity of cluster sizes exists (e.g. PCPs per clinic, and patients per PCP). A proper multilevel model allows for optimal weighting based on the estimated variance components

(e.g. Gauss-Markov) and yields both an efficient summary of the overall intervention effect, as well an estimate of the variability in the magnitude of effect across clinics. However, we will not rely on the covariance model being correct for statistical inference and will use a robust (empirical) standard error. With greater than 100 total clusters (clinics) we expect valid inference and proper test size and do not anticipate needing to perform any correction such as the jackknife[7] (recommended when the number of clusters is small).

In our analysis we effectively assume that individual patients are nested within a single provider. However, in practice a patient may change providers during the follow-up year over which the primary outcome is captured. However, our basic mixed model covariance structure will simply use the assigned primary provider at the index imaging time. Therefore, we do not rely on model-based standard errors since the covariance structure may not match the true within-clinic covariance structure. We will use robust standard errors clustering at the clinic level, and therefore our analysis is valid even if there are changes in patient provider leading to an incorrectly specified covariance structure. Robust standard errors remain valid when a covariance model is not correctly specified. Furthermore, key secondary analysis of the primary outcome will directly use GEE and only cluster at the clinic level and provider level linkages are not used (nor needed) for simple GEE analysis.

Secondary Analyses of Primary Outcome: We will conduct additional secondary analyses that evaluate the sensitivity of the multilevel model to the assumed basic random effects structure. We have included in the primary model multilevel random intercepts and a random effect for the clinic-level intervention. However, we will expand the random effects structure to also permit random slopes on time for both clinics and providers. Given the relatively short duration of follow-up with only six (6) total measurement times we do not expect strong heterogeneity across providers or clinics in cluster-specific temporal trends. Figure 2 shows an example of hypothetical data series for two clinics (assuming aggregation of providers to a clinic summary) and illustrates both the staggering of the crossover time and the potential to observe clinic-specific intervention effects. This figure also illustrates the fact that separating random effects of time (linear) from random effects of intervention would be difficult since time and intervention status are correlated give the unidirectional crossover from control to intervention. In addition, we will use GEE as a covariance model robust inference method and therefore can produce valid point estimates and confidence intervals without relying on correct covariance specification. Details of model choice and comparison of alternative models for longitudinal cluster level crossover trials is presented in French and Heagerty (2008) and comparison of alternative approaches is recommended.

Models for time and intervention effect: Our primary analysis adopts a linear adjustment for calendar time in order to remove any large-scale temporal trends that may bias estimates of intervention effects. However, our basic regression model assumes a common (adjusted) mean for all times after the initiation of intervention. In practice there may be a delay in the impact of intervention so alternative models will be considered that incorporate a delayed and/or gradual effect of intervention. For example, the basic coding of the time-dependent variable $Status_{kt}$ takes the value 0 pre-intervention and the value 1 post-intervention. Delay in the impact of intervention can be accommodated using alternatives such as: 0 pre-intervention; 0.5 for quarter 1 after intervention; and 1 for all other post-intervention quarters. Such a modified model would allow full impact of the intervention to require two quarters of exposure. We will conduct secondary analyses to explore alternative models for the accumulation or delay of the intervention effect.

Secondary Outcome Analysis: We will also analyze the impact of intervention on the rate of opioid prescription using Generalized Linear Mixed Models (GLMMs). For Aim 1b let $Y_{ijk}=1$ if opioids were prescribed within a given timeframe (e.g. 30 days or 90 days) to patient i $(i= 1,2,\ldots,n_j)$ seen by primary care provider j $(j=1,2,\ldots,n_k)$ within clinic k $(k = 1,2,\ldots,110)$. Analysis for this outcome will use a logistic mixed model given as:

$$\begin{aligned} logit(p_{ijk}) = {} & \beta_0 + \beta_1 \cdot Time_t + \beta_2^T \cdot Age_{ijk} + \beta_3^T \cdot Modality_{ijk} + \beta_4^T \cdot Size_k + \beta_5^T \cdot Site_k + \lambda_0 \cdot Status_{kt} + && \textit{mean model} \\ & b_{k,0} + b_{k,1} \cdot Status_{kt} + && \textit{clinic random effects} \\ & a_{jk,0} && \textit{provider random effects} \end{aligned}$$

where p_{ijk} denotes the probability that Y_{ijk} =1. Our secondary outcome analysis parallels the primary and will be based on a natural multilevel mixed model, with additional robust secondary analysis provided by GEE. For Aim 1c we will use Y_{ijk} =1 if CT or MR imaging occurs within a specified timeframe (e.g. 90 days or 12 months) after the index imaging event.

Medical costs (Aim 1d): Spine-related costs of care will be estimated using two approaches. First, we will use the spine-related RVU calculated in Aim 1a and estimate clinical-level, spine-intervention expenditures using the annual Medicare-determined payment amount per RVU (e.g., CY2013 = $34.023 per RVU). (reference: http://www.cms.gov/Outreach-and-Education/Medicare-Learning-Network-LN/MLNProducts/downloads/medcrephysfeeschedfctsht.pdf)

Second, as a proxy for costs of spine care, we will use a standard set of reimbursement amounts, i.e., CMS-based payments, and estimate clinic-level spine-related aggregate expenditures by applying CPT-based payment amounts to specific spine-intervention events (e.g., imaging, office visits, procedures, other). We will present monthly and annual means, medians, and ranges of clinic-level cost estimates, prior to and subsequent to implementing the epidemiological intervention. We will assess the level of right-skewness in the expenditure estimates and use t-tests to compare arithmetic means for clinic-level expenditures. In the case of considerable skewness, we will test for differences in logarithmically transformed mean clinic-level expenditures (before and after implementing intervention). We will also describe categories of prescriptions ordered, when available in the electronic medical records for a health system, and estimate costs for prescribed spine-related medications

Analysis for Aim 2: The hypothesis of Aim 2 is that there will be a differential effect of the intervention according to the imaging modality used. In order to test this hypothesis we will analyze patient-level data according to the appropriate LMM or GLMM given above, but including the interactions between $Modality_{ijk}$ indicators (modeled using two indicator variables coding CT and MR, with plain film as the reference) and $Status_{kt}$. A test of the interaction terms (2 degree of freedom Wald test) will be used to test the null hypothesis that the effect of the intervention does not vary according the imaging modality.

Analysis for Aim 3: The hypothesis of Aim 3 is that there will be a differential effect of the intervention according to the results that are found in the imaging report. We will use an additional variable, $ImageFinding_{ijk}$, that takes the value 1 if a significant image finding is present, and 0 otherwise (see detail regarding variable specification in protocol). We will test the null hypothesis that the interaction between $ImageFinding_{ijk}$ and $Status_{kt}$ is zero using a Wald test.

Power Calculations: Our UH2 efforts with respect to sample size and statistical power focused on two key items. First, an important aim of our UH2 Working Group 2 was to obtain an accurate clinic and provider count for each health system. We will now randomize n=110 clinics (1,824 PCPs), which is slightly lower than the n=128 clinics (1,898 PCPs) assumed in our initial project application. However, the majority of the clinics that were dropped were those with only one PCP and therefore would not have contributed much information to the analysis. Second, in our Working Group 3 we sought to develop and characterize a composite RVU summary to be used as the primary outcome measure in this study. In our UH2 project application we discussed statistical power in the context of an important secondary outcome measure, a reduction in subsequent opioid prescription rates. We now present statistical power for the primary outcome measure using data from the BOLD Registry to inform key design parameter estimates.

To our knowledge, off-the-shelf calculators do not exist that would adequately characterize statistical power for a stepped wedge cluster randomized trial with a varying number of sampling units between clusters. We therefore utilized simulation methods to generate and analyze data that closely mimics the design characteristics we anticipate for this study. With a simulation approach, we were able to include estimates of both patient and clinic-level variability and implement the proposed primary analysis methods: random intercept linear mixed effects models for RVU outcomes; and generalized linear mixed models for opioid prescription rates. All simulations

were conducted using R (version 3.0.1) with the *lmer* and *glmmPQL* functions implementing mixed model estimation.

Power for Primary Outcome: In the BOLD Registry we identified 639 patients in the Kaiser Permanente and Henry Ford health systems that had a qualifying lumbar image within 6 weeks of a PCP visit, the majority (74%) of which occurred within 7 days. As one would expect with a measure of health care utilization intensity, patient-level RVUs are positively skewed. In our simulations and in the future analysis of study data, we therefore utilize an approximately normalizing transformation of log(RVU + 1) but will make interpretations regarding effect size back on the original RVU scale.

Figure 2: Statistical power for spine-related RVU

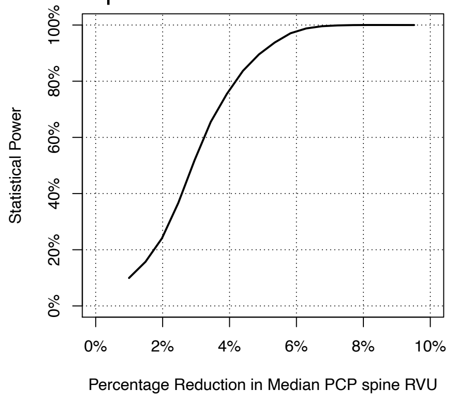

With log-transformed BOLD Registry RVU data, we fit a linear mixed effects model adjusting for image type (advanced vs. plain film) and study recruitment site and estimated the variance components for clinic (0.026) and the residual error term (1.230). The observed intra-class correlation coefficient (ICC) across clinics was 0.013 (95% CI: 0.000 to 0.046). In this subset of BOLD data, the number of PCPs with multiple patients was too few to inform the PCP-level variance component and it is therefore conservatively included in the error term variance for power simulations.

The numbers of clinics and primary care providers were considered fixed for each simulation and we assumed that each provider would provide data for all study time periods. For a range of potential RVU effect sizes, we generated 1,000 simulated data sets and performed mixed model estimation with each data set. In Figure 2, we show statistical power for the primary outcome measure of PCP spine-related RVU under the proposed study design. The study has greater than 90% power to detect reductions in the median spine-related RVU of 5.0% or larger. For a patient receiving a lumbar CT, a 5% reduction in spine RVU translates into one fewer additional lumbar CT scan on average compared to a patient unexposed to the LIRE intervention.

Power for Secondary Outcome: Using the updated clinic and provider listing, we repeated the UH2 power analyses for a reduction in subsequent opioid prescriptions. We again used an average baseline opioid prescription rate of 22%, suggested from the pilot manuscript[9] to anchor the effect size of percent reduction in the baseline rate. A clinic-specific baseline opioid prescription rate was drawn from a Beta(α=6, β=20) distribution; we used this rate to draw a baseline opioid prescription rate random effect for each primary care provider using a clinic-specific log-normal distribution. We generated 1,000 simulated data sets using the effect size of a 7.5% reduction in the opioid reported in our UH2 application and evaluated each using a GLMM assuming random intercepts. With 110 clinics randomized, the study remains well powered (88.9% power) to detect a reduction in the rate of opioid prescriptions of 7.5% or larger (e.g. 22% down to 20.4%).

Statistical Analysis Plan References

1. Hussey MA, Hughes JP. Design and analysis of stepped wedge cluster randomized trials. *Contemp Clin Trials* 2007;28:182-91.
2. Martin B, Mirza SK, Lurie JD, et al. Validation of an administrative coding algorithm to identify back-related degenerative diagnoses. *International Society for the Study of the Lumbar Spine (ISSLS)*. Scottsdale, AZ, 2013.
3. Martin BI, Gerkovich MM, Deyo RA, et al. The association of complementary and alternative medicine use and health care expenditures for back and neck problems. *Med Care* 2012;50:1029-36.
4. Martin BI, Mirza SK, Franklin GM, et al. Hospital and surgeon variation in complications and repeat surgery following incident lumbar fusion for common degenerative diagnoses. *Health Serv Res* 2013;48:1-25.
5. http://www.fda.gov/drugs/informationondrugs/ucm142438.htm, accessed September 13, 2013.

6. Diggle PJ, Heagerty PJ, Liang KY, Zeger SL. Analysis of Longitudinal Data. Second Edition ed: Oxford University Press; 2002.
7. Efron B, Stein C. The jackknife estimate of variance. *Annals of Statistics*. 1981;9(3):586-96.
8. French B, Heagerty PJ. Analysis of longitudinal data to evaluate a policy change. *Statistics in Medicine* 2008; 27(24):5005-25.
9. McCullough BJ, Johnson GR, Martin BI, Jarvik JG. Lumbar MR imaging and reporting epidemiology: do epidemiologic data in reports affect clinical management? Radiology. 2012;262(3):941-6. PMCID: 3285226.
