# Supplementary material for: The Effect of Including Benchmark Prevalence Data of Common Imaging Findings in Spine Image Reports on Health Care Utilization Among Adults Undergoing Spine Imaging: A Stepped-Wedge Randomized Clinical Trial
Source: JAMA Netw Open. 2020 Sep 4;3(9):e2015713. doi: 10.1001/jamanetworkopen.2020.15713 (PMC7489827; doi:10.1001/jamanetworkopen.2020.15713)
Supplement: Supplement 3. — Data Sharing Statement [file jamanetwopen-e2015713-s003.pdf]

Data Sharing Statement

Jarvik. The Effect of Including Benchmark Prevalence Data of Common Imaging Findings in Spine Image Reports on Health Care Utilization Among Adults Undergoing Spine Imaging. *JAMA Netw Open*. Published September 04, 2020. 10.1001/jamanetworkopen.2020.15713

Data
Data available: Yes
Data types: Deidentified participant data, Data dictionary
How to access data: We plan to make de-identified data available through a private archive hosted by the Resource Core of the University of Washington Clinical Learning, Evidence And Research (CLEAR) Center for Musculoskeletal Disorders https://theclearcenter.org/about/resource-core/
When available: With publication

Supporting Documents
Document types: None

Additional Information
Who can access the data: We will make de-identified, clinic level data available to interested parties in XML, SAS XPORT, and R formats through a web-based request form hosted on a study-specific secured web page. We will also make available appropriate data dictionaries. Prior to sharing de-identified data beyond study investigators, we will require that requestors submit a Data Use Agreement (DUA), a proposal describing for what purpose they will use the data, and a data analysis plan. The DUA will stipulate that others will not use the data in an attempt to identify individual primary care providers, patients or clinics. CLEAR Center Resource Core Director and Associate Director will review requests for scientific soundness. Representatives of the LIRE data collection sites will also have the opportunity to review and approve requests. Costs of proposal review and data preparation will be borne by the requester.
Types of analyses: CLEAR Center Resource Core Director and Associate Director will review requests for scientific soundness. Representatives of the LIRE data collection sites will also have the opportunity to review and approve requests.
Mechanisms of data availability: Costs of proposal review and data preparation will be borne by the requester.
